# Hunter-gatherer sea voyages extended to remotest Mediterranean islands

**DOI:** 10.1038/s41586-025-08780-y

**Published:** 2025-04-09

**Authors:** Eleanor M. L. Scerri, James Blinkhorn, Huw S. Groucutt, Mathew Stewart, Ian Candy, Ethel Allué, Aitor Burguet-Coca, Andrés Currás, W. Christopher Carleton, Susanne Lindauer, Robert Spengler, Kseniia Boxleitner, Gillian Asciak, Margherita Colucci, Ritienne Gauci, Amy Hatton, Johanna Kutowsky, Andreas Maier, Mario Mata-González, Nicolette Mifsud, Khady Niang, Patrick Roberts, Joshua de Giorgio, Rochelle Xerri, Nicholas C. Vella

**Affiliations:** 1 Human Palaeosystems Group, Max Planck Institute of Geoanthropology, Jena, Germany; 2 Department of Classics and Archaeology, Faculty of Arts, University of Malta, Msida, Malta; 3 Institute of Prehistoric Archaeology, University of Cologne, Cologne, Germany; 4 Department of Archaeology, Classics and Egyptology, University of Liverpool, Liverpool, UK; 5 Australian Research Centre for Human Evolution, Griffith University, Brisbane, Queensland Australia; 6 Department of Geography, Royal Holloway University of London, Egham, UK; 7 Institut Català de Paleoecologia Humana i Evolució Social (IPHES-CERCA), Tarragona, Spain; 8 Departament d’Història i Història de l’Art, Universitat Rovira i Virgili (URV), Tarragona, Spain; 9 Department of Archaeological Sciences, Faculty of Archaeology, Leiden University, Leiden, The Netherlands; 10 Department of Archaeology, Max Planck Institute of Geoanthropology, Jena, Germany; 11 Curt-Engelhorn-Centre Archaeometry, Mannheim, Germany; 12 Domestication and Anthropogenic Evolution Research Group, Max Planck Institute of Geoanthropology, Jena, Germany; 13 Superintendence of Cultural Heritage, Valletta, Malta; 14 Evolutionary Ecology Group, Department of Zoology, University of Cambridge, Cambridge, UK; 15 Department of Geography, Faculty of Arts, University of Malta, Msida, Malta; 16 Department of Structural Changes of the Technosphere, Max Planck Institute of Geoanthropology, Jena, Germany; 17 Institute for Archaeological Sciences, University of Tübingen, Tübingen, Germany; 18 Département d’Histoire, Université Cheikh Anta Diop de Dakar, Dakar, Senegal; 19 isoTROPIC Research Group, Max Planck Institute of Geoanthropology, Jena, Germany; 20 Department of Archaeology, University of York, York, UK; 21 National Museum of Natural History, Heritage Malta, Mdina, Malta

**Keywords:** Archaeology, Palaeoecology

## Abstract

The Maltese archipelago is a small island chain that is among the most remote in the Mediterranean. Humans were not thought to have reached and inhabited such small and isolated islands until the regional shift to Neolithic lifeways, around 7.5 thousand years ago (ka)^1^. In the standard view, the limited resources and ecological vulnerabilities of small islands, coupled with the technological challenges of long-distance seafaring, meant that hunter-gatherers were either unable or unwilling to make these journeys^2–4^. Here we describe chronological, archaeological, faunal and botanical data that support the presence of Holocene hunter-gatherers on the Maltese islands. At this time, Malta’s geographical configuration and sea levels approximated those of the present day, necessitating seafaring distances of around 100 km from Sicily, the closest landmass. Occupations began at around 8.5 ka and are likely to have lasted until around 7.5 ka. These hunter-gatherers exploited land animals, but were also able to take advantage of marine resources and avifauna, helping to sustain these groups on a small island. Our discoveries document the longest yet-known hunter-gatherer sea crossings in the Mediterranean, raising the possibility of unknown, precocious connections across the wider region.

## Abstract (web summary)

Archaeological discoveries from Malta suggest that humans were present on the Maltese islands from around 8,500 years ago, providing evidence that Mesolithic hunter-gatherers made sea crossings as long as 100 km.

## Main

**Fig. 1:**
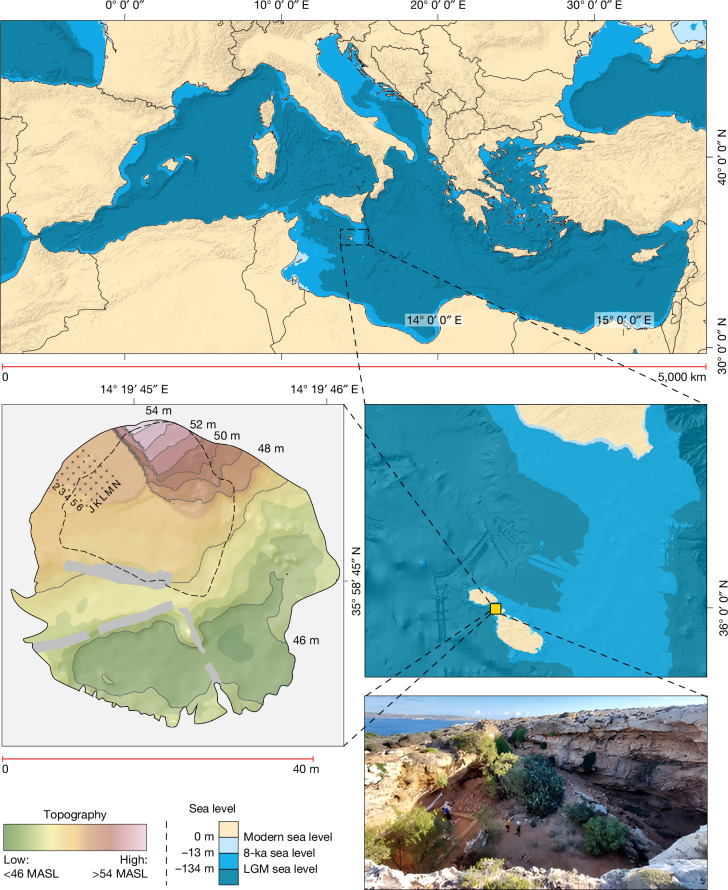
Maps and image of Latnija. Top, the position of Malta in the Mediterranean. Bottom left, digital elevation model of Latnija, showing the current dripline in dashed lines. Bottom right, the site, showing the sea channel and Gozo in the background, with past sea levels based on a previous study^49^. LGM, Last Glacial Maximum; MASL, metres above sea level. The edge of Trench 4 is denoted by the hessian sacks. Data from refs. ^50,51^ and created using ArcMap 10.5.

The emergence of long-distance seafaring varies considerably around the globe, with an early appearance in Southeast Asia and Sahul seemingly not replicated until later in other regions, such as the islands off the African coast^5–9^. With a sea crossing of around 100 km from Sicily, and around three times as far to the Maghreb, the Maltese Archipelago is among the most remote groups of islands in the Mediterranean, the world’s largest inland sea (Fig. 1). Sea-level rise rapidly submerged the low-lying, now around 95 m deep, hypothesized land bridge from Malta to Sicily around 13 ka. Over the next few thousand years, both Sicily and the Maltese islands reached their current configurations, with Malta now having a combined landmass of just 316 km^2^ (ref. ^10^). Like other small Mediterranean islands, and particularly given its semi-arid climate, Malta was inferred to have been too small and remote to support human populations before the adoption of farming and more advanced seafaring technology (see Supplementary Information 1 for discussion). The general consensus has been that hunter-gatherers only journeyed to Mediterranean islands that were large, and/or easy to reach, such as through chains of connecting islands, proximity to the mainland or favourable currents^1,2,11^ (Supplementary Information 1).

**Extended Data Fig. 1:**
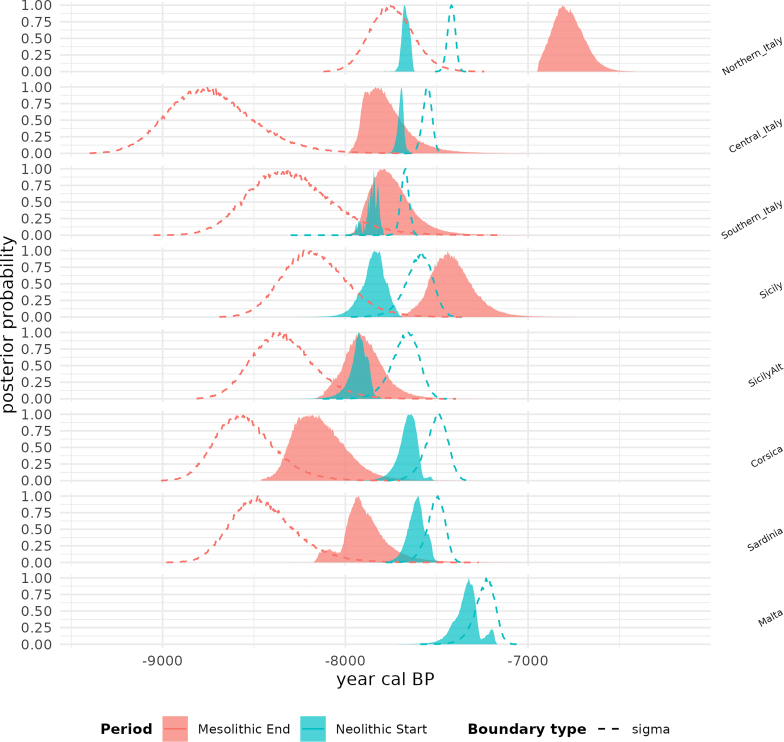
Regional OxCal phase model of the Mesolithic-to-Neolithic transition. OxCal phase modelling of the estimated start and end dates for the Mesolithic and Neolithic phases in and around Malta^125^ using the IntCal20 terrestrial calibration curve^55^. Results indicate a general geographical cline in the spread of the Neolithic in mainland Italy from north to south, to Sicily, Sardinia, and Corsica, and then finally to Malta. See the OxCal script in https://github.com/wccarleton/mesoneomalta for specifics. As the legend indicates, dashed lines represent the ‘sigma’ type boundary whereas filled areas represent the ‘uniform’ type.

**Extended Data Fig. 2:**
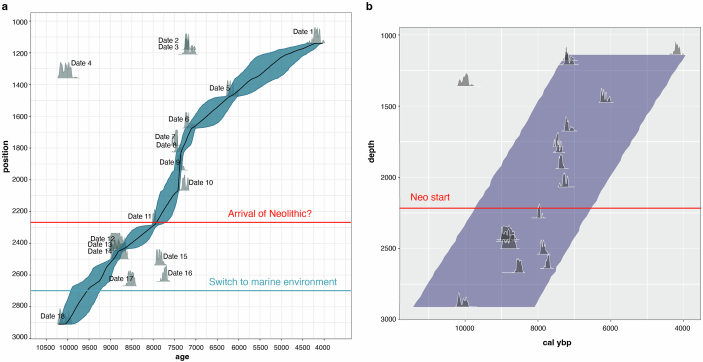
Bayesian evaluation of the earliest Maltese Neolithic. **a**, Plotted radiocarbon dates available for the sequence from the Salina Deep record in Malta, along with the pollen sequence reported by Farrell and colleagues^48^, using the Bchron R package^52^, one of the methods used by Hunt and colleagues^47^. Results show many potentially intrusive samples used to date the sediments and very few sequences of dates in strict stratigraphic order. **b**, Our Bayesian regression model to relate depth to age in the Salina Deep core using the IntCal20 calibration curve^55^ to calibrate the dates. The results suggest that the first Neolithic evidence in the Salina Deep record has a date with a broad error range of around two thousand years. Depth shown = cm.

**Extended Data Fig. 3:**
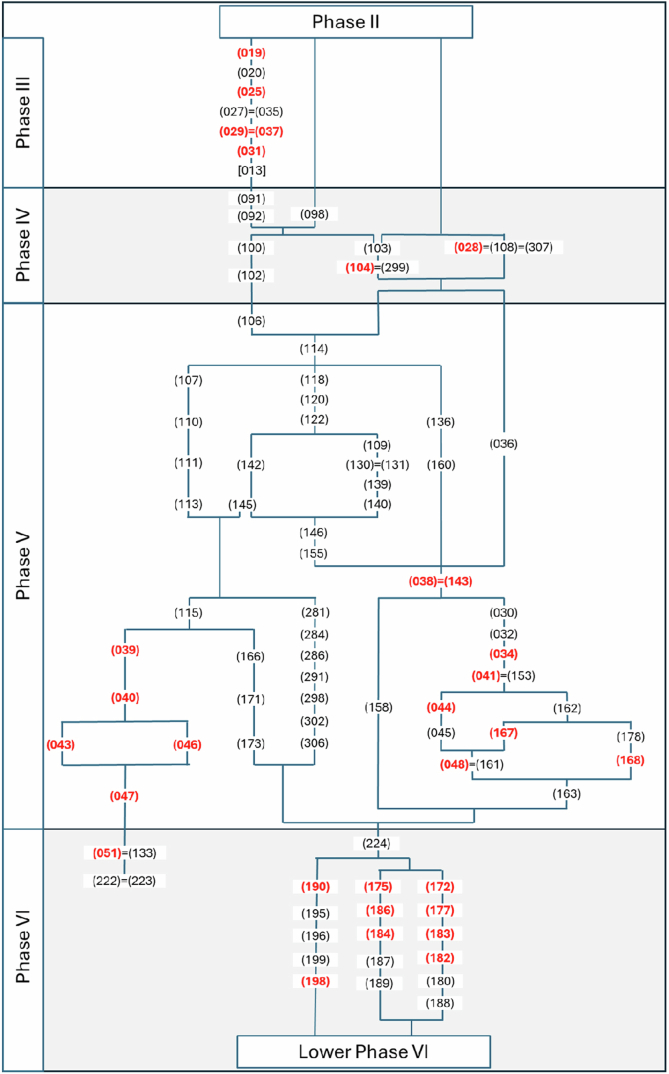
Harris matrix of the Latnija excavation contexts organized by phase. Harris matrix of the Latnija excavation organized by phase illustrating the stratigraphic relationship between excavation contexts, with numbers shown in red indicating deposits containg dated material.

Previous research has supported this view, with the evidence suggesting that the first people to reach Malta were Neolithic farmers, associated with impressed ware pottery, stemming from the Sicilian ‘Stentinello’ phase of the Neolithic^12–14^. These farmers were assumed to have introduced crops and domesticated and commensal fauna into a pristine island ecosystem^14^. The directly dated and secure evidence for the start of the Neolithic in Malta indicates an age of around 7.4 ka (ref. ^15^), which is consistent with the regional chronology of the spread of the Neolithic from southern Italy^16^. It is also consistent with our own chronological model (Methods, Supplementary Information 1 and Extended Data Fig. 1) based on an extensive database of radiocarbon dates with good contextual information, indicating that the earliest Neolithic in southern Italy and Sicily dates to around 7.9–7.5 ka, and later in Malta at around 7.4–7.1 ka. Although occasional claims for an earlier Neolithic in both Sicily and Malta have been suggested, they are problematic because of radiocarbon dates and age models with high levels of uncertainty, in addition to being inconsistent with the regional chronology mentioned above (Supplementary Information 1 and Extended Data Figs. 2 and 3). Although claims of a far earlier Pleistocene human presence on Malta have also been made^17,18^, they have so far failed to stand up to scrutiny on morphological and chronological grounds (Supplementary Information 1).

Here we provide decisive evidence for a pre-Neolithic human presence on the Maltese islands, in the form of a previously unknown Mesolithic phase characterized by the presence of Holocene hunter-gatherers. This discovery casts new light on the age and extent of Mesolithic sea crossings in the Mediterranean, and on hunter-gatherer interactions with endemic island fauna. Joint investigations led by the Max Planck Institute of Geoanthropology and the University of Malta have unravelled a deep archaeological sequence at the site of Latnija (Lat-nee-yuh). The site is located in a large doline in the Mellieħa area of northern Malta (Fig. 1), in the vicinity of several fresh water sources and close to a coastline that has both sandy beaches and rocky shorelines^19^. Detailed excavations between 2021 and 2023 revealed early-to-mid-Holocene-aged sediments that contain stone tools, hearths, ash-tips and a range of wild flora and fauna, including marine gastropods, fish and marine mammals. These findings reveal the cultural and ecological context of the final stages of the Mesolithic, before the Neolithic transition in the region. Crucially, they also reveal the longest sea crossing yet documented in the Mediterranean by hunter-gatherers, highlighting the considerable seafaring abilities of late European hunter-gatherers. Even in the subsequent Neolithic, there are only occasional indications of such long sea crossings in the Mediterranean^20^. Our findings upend the established notions that small and remote islands were beyond reach in the Mesolithic world.

**Fig. 2:**
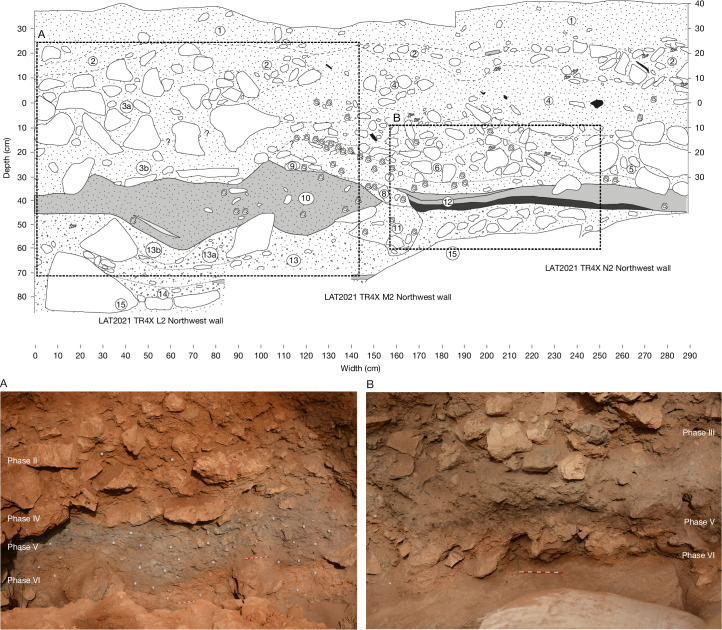
Stratigraphic section of the northwest wall. Top, illustration of the key stratigraphic sequence (numbered Beds are described in Supplementary Information 2) highlighting a thick bed of ash (A; bottom left), and a hearth deposit or combustion structure (B; bottom right), with combustion residue (ash on top), thermal impact zone and a natural substrate (Supplementary Information 3), at the base of the Mesolithic Horizon. Note also the *Phorcus turbinatus* tip line, starting in the mid-right of box A.

We excavated a 5 × 5-m trench (designated Trench 4) beneath an overhang on the north-western edge of the doline, in the lee of the prevailing northwesterly wind (Methods). We divided the trench plan into an alphanumeric grid of 1-m^2^ squares (J–N, 2–6; Fig. 1) and recorded the position of all artefacts and bones larger than 20 mm in three dimensions using a total station. We describe the excavated sequence in six phases (labelled Phase I–VI from top to bottom), combining distinct differences in depositional processes (Supplementary Information 2) and material culture. The base of our excavated sequence (Phase VI; Fig. 2, Beds 15–13) comprises a naturally formed fine-grained cave sediment, pale orange to pink in colour (dominated by fine sands and silts), on top of sloping boulders. The character of the deposits in Phase VI is in stark contrast to that of the deposits that overlie them in Phases V–III, in which the presence of ash, fauna and shell-rich sediments presents conspicuous evidence for anthropic activity, which we refer to as the Mesolithic Horizon (Fig. 2).

The earliest Mesolithic deposit in Phase V (Fig. 2, Beds 12–10) is marked by discrete hearth features, overlain by a bed of grey ash-rich sediment of varying thickness, a rich faunal assemblage and stone tools. The lowest hearth, from N2 (Phase V; Fig. 2 (B)), comprises two superimposed differentiated layers, as described from other sites and identified through experimental archaeology^21,22^ (Supplementary Information 2 and 3 and Fig. 2). This includes a heterogeneous light-grey ashy combustion residue of varying thickness (typically 6–12 cm) overlying a homogeneous brown-to-black thermally impacted sediment, which are distinct from the underlying Phase VI substrate (Fig. 2). Fourier transform infrared spectroscopy (FTIR) analyses confirmed that combustion residues are composed mainly of pyrogenic calcite (ash), with some thermally altered clay, which might have been introduced between burning episodes; more limited pyrogenic alteration is evident in the thermal impact zone (Supplementary Information 3). Analyses showed that higher concentrations of phytoliths were present in the same samples in which ash has been documented, compared with other parts of the combustion structure (thermal impact) and control samples (Supplementary Information 3). This indicates that the phytoliths (described below) reached the site as a result of an anthropic contribution in the form of fuel or related to use of the combustion structure. Micromorphological analysis of the sediments directly below the hearth feature in N2 show evidence for enhanced reddening and enrichment in iron oxides relative to the natural cave floor sediments (Methods and Supplementary Information 2). This is consistent with this combustion feature being in situ. In L2 and M2, micromorphology and detailed sediment analysis indicate a more complex relationship between deposits that are rich in combustion products and the sediments that underlie them; some show evidence for in situ burning, whereas others indicate erosion or cutting into the underlying sediments and the localized remobilization and redistribution of ash-rich materials (Supplementary Information 2).

The onset of episodes of cave-wall collapse is observed at the top of this ash-rich deposit, marked by a clast-dominant layer closer to the cave wall, grading to finer sediments beyond the dripline, also containing fauna and artefacts (Phase IV; Fig. 2, Bed 3b). A subcircular pit (Phase III; Fig. 2, Beds 9–5) has been dug through this layer, truncating the top of Phase V deposits; this pit contains discrete dumps of marine shells and ashes (Fig. 2 and Supplementary Information 2), as well as stone tools. The Mesolithic Horizon is sealed by more conspicuous episodes of cave-wall collapse, including both clast- and matrix-dominated cave sedimentation that contains artefacts attesting to later prehistoric, historic (Phase II; Fig. 2, Beds 4–1) and modern (Phase I) occupations.

## Chronology

**Fig. 3:**
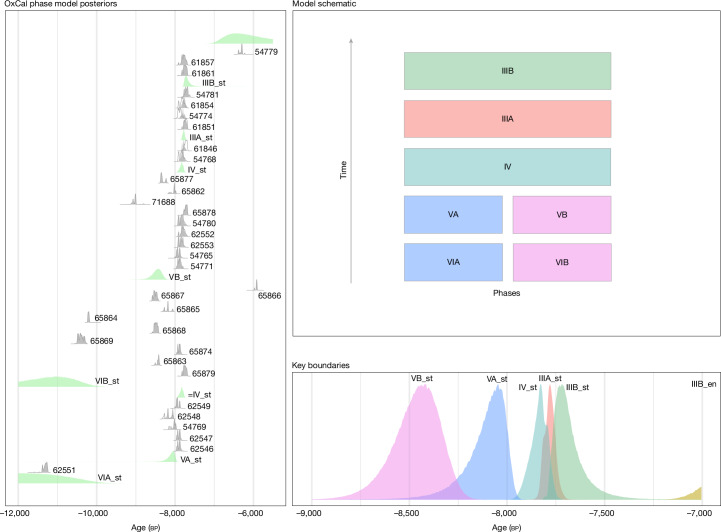
Chronological model. Model (OxCal 4.4; IntCal20) shows the phase boundaries in the Mesolithic Horizon, Phases III–V. The model indicates that the mean start date of the Mesolithic Horizon is 8.5 ka. Laboratory codes are included in the left box.

We selected samples for chronometric dating to constrain the age range of key sedimentary deposits, the boundaries of major sediment phases and the shells of edible marine gastropods (*Phorcus turbinatus*; *n* = 49) accumulated by humans. A total of 32 dates (obtained using accelerator mass spectrometry) on charcoal were used to constrain the different phases at the site. One additional date was also recovered on bone, whereas insufficient collagen meant that all other attempts to date bone failed (Supplementary Information 4). These dates were then calibrated to estimate the boundaries between depositional phases with a Bayesian phase model (Fig. 3, Methods and Supplementary Information 4). The results show that occupation of the site began by around 8.5 ka, well before the earliest-known dates for the arrival of Neolithic farmers in Malta and the wider region—attesting to the presence of Mesolithic hunter-gatherers. The end of the Mesolithic is more difficult to determine without precise dates for the beginning of the Neolithic at the site, but it seems to end with the arrival or establishment of the first farmers (Supplementary Information 4). The *P. turbinatus* shells were corrected for the marine reservoir effect (MRE) and calibrated ages were calculated (Methods and Supplementary Information 4). The *P. turbinatus* shells range from around 8.6 ka to 7.5 ka, supporting the charcoal age model. Crucially, the limited variability of these shell ages supports the intact stratigraphic character of the Mesolithic Horizon, a feature particularly visible in the conspicuous tip lines in Phase III (Fig. 2 and Supplementary Information 2). Overall, the consistent chronological data and highly resolved stratigraphy support the integrity and well-dated character of the Latnija sequence.

## Stone tools

A total of 64 lithics (knapped stone tools) were recovered from the Mesolithic Horizon (Phase V–III) deposits (Supplementary Information 5). Except for one chert artefact, all stone tools were made of limestone, much of which was clearly procured in the form of beach cobbles or pebbles, with the remainder sourced from terrestrial outcrops. This contrasts with younger, Neolithic, assemblages from Malta, which are made from chert (both local and imported) and small amounts of imported obsidian^14,23,24^. Cores, blades and bladelets and retouched tools are rare in the Latnija Mesolithic assemblage, which is instead focused on simple flakes produced by hard hammer percussion. The main reduction products were squat and often cortical flakes, with generally unidirectional dorsal scar patterns. In contrast to penecontemporaneous assemblages from Sicily and other adjacent areas, which generally exhibit complex technologies and geometric forms (for example, trapezes), the lithic material from Latnija most resembles relatively expedient Mesolithic lithic technology from Sardinia^25^ (Supplementary Information 1 and 5). The simple character of the Latnija lithic assemblage might reflect the poor quality of the limestone used and expediency, but could also reflect other factors, including demographic aspects, such as small population size and isolation.

## Faunal remains

**Fig. 4:**
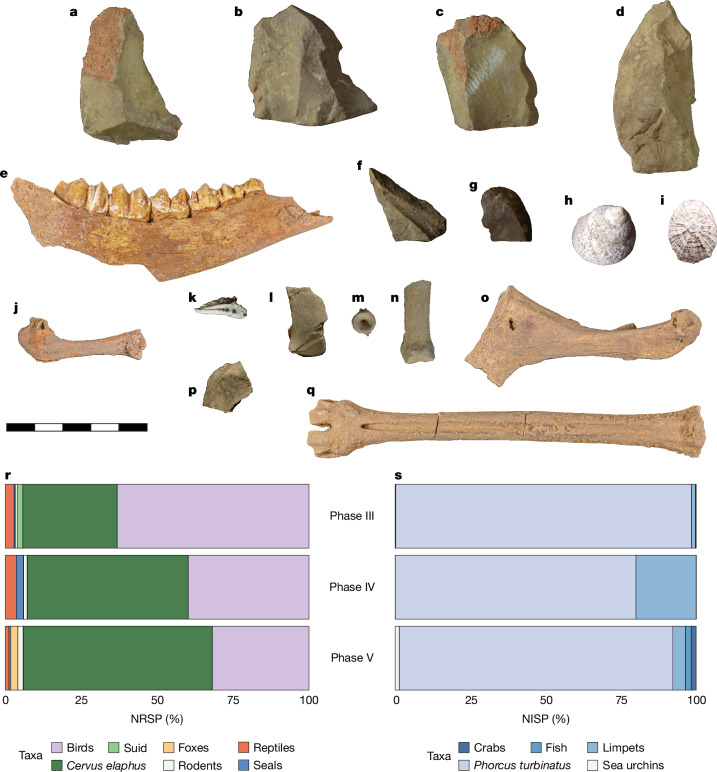
Fauna and lithics from the Mesolithic Horizon. **a**–**q**, Selected fauna and lithics from the Mesolithic Horizon. All the fauna are wild. Limestone flakes (**a**–**d**,**f**,**g**), red deer left mandible (**e**), and metatarsal (**q**), *Phorcus turbinatus* (**h**), *Patella* sp. (**i**), crab claw (**k**), turtle or tortoise carapace (**l**,**p**), fish vertebra (**m**), seal proximal phalanx (**n**), bird humerus (**j**) and coracoid (**o**). Scale bar is 50 mm and applies to all. **r**, Percentage of the number of reported specimens (NRSP) of piece-plotted bone. This includes terrestrial animals and marine mammals. **s**, Percentage of the number of identified specimens (NISP) of fish and marine invertebrates from squares L2 and N2 recovered during wet sieving and flotation.

A total of 955 piece-plotted specimens (larger than 20 mm) from the Mesolithic Horizon were recorded during the 2021 and 2022 seasons, in addition to many smaller fragments recovered during sieving and flotation (Supplementary Information 6). The fauna is all wild, and overall is dominated by red deer (*Cervus elaphus*), birds and marine gastropods (*P. turbinatus* in particular, but also limpets), with the latter so far comprising some 10,000 shells (Fig. 4 and Supplementary Information 6). Small numbers of reptiles (for example, turtles and tortoises), fish (for example, groupers), crustaceans (crabs), echinoderms (sea urchins) and marine mammals (seals) were also found (Fig. 4). In line with the extensive evidence for anthropic combustion, around 25% of taphonomically studied faunal remains, including those of red deer, birds and tortoises, as well as the marine gastropods, had evidence of burning or charring (Supplementary Information 6). Although a detailed taphonomic analysis is ongoing, other traces of anthropogenic activity can also be observed, including probable percussion notches and green fracturing.

The use of marine resources, including not only small gastropods and crustaceans, but also large marine mammals, matches well with subsistence behaviours observed at other Mesolithic sites in the Mediterranean^26–28^. Notably, studies of Neolithic and younger sites in Malta have uncovered little evidence for marine resource exploitation—and archaeological and isotopic studies suggest that people had diets that were focused mostly on terrestrial resources, including livestock and wild and domesticated plants^15,29^. The Mesolithic deposits at Latnija therefore represent a unique level of marine resource engagement in Malta and a substantially different diet to that of later, farming communities.

## Environmental reconstruction

Archaeobotanical analyses were further used to understand the environmental context of the Mesolithic Horizon. Grasses were abundant and are represented by many different phytolith morphologies (Supplementary Information 3). Most of the grasses correspond to C_3_ types, although phytolith morphologies ascribed to C_4_ plants are also present. Pollen analysis of two samples from Phase V provides evidence of an open shrub vegetation consisting of *Erica multiflora* and *Euphorbia melitensis*, with patches of *Pistacia lentiscus* shrub communities occupying areas in which higher moisture levels were present and soil development occurred.

Macrobotanical samples were recovered from the systematic flotation of sediments (Methods). Seeds of a few small, wild herbaceous plants were identified, including a small-seeded grass (Poaceae), small-seeded legumes and seeds of a member of the Chenopodioideae, as well as *Mercurialis* cf. *annua* and *Vaccaria hispanica*. All of these plants grow wild on Malta today and might have been introduced to the site either by the burning of brush or through natural processes, such as the activity of rodents or birds, and inadvertently burned with cave sediments. Complementing the phytolith and macrobotanical data, the charcoal analyses reflect a shrubby vegetation adapted to the island environment, and characterized by an open scrubland dominated by *Pistacia* cf. *lentiscus*, *Juniperus* and *Tetraclinis* among other shrubs, similar to the present day. These data together indicate the presence of vegetative communities typical of the Early to Middle Holocene in the Central Mediterranean region, which have been linked to the onset of more humid climate conditions^30–32^. These observations were further complemented by isotopic analyses of ungulate and rodent teeth from the site (Methods and Supplementary Information 3), which indicate a fairly stable mixture of dry C_3_ grassland, scrubland and woodland. In terms of plant use, the presence of the spheroid echinate phytolith morphotype in the hearth is noteworthy. This probably corresponds to the indigenous *Chamaerops humilis* (Mediterranean fan palm)^33^. *Chamaerops humilis* and other palms have a wide range of uses, ranging from textiles to construction materials and food, among others. However, the greater presence of these morphotypes in the samples related to the combustion residue seems to indicate that they were also used as fuel. Anthracological analyses revealed that the most common fuel was *Pistacia* cf. *lentiscus* wood, which still grows in the vicinity of the site today. Wild seeds of grasses, and a few other low-growing herbaceous plants, were recovered in a carbonized state, either representing the burning of vegetation around the site or the construction of a hearth on top of seed-laden sediments.

## Discussion

The evidence from Latnija confirms a Mesolithic occupation of the Maltese islands spanning from around 8.5 ka to 7.5 ka, which differs markedly from younger, agro-pastoral societies in technology, raw materials, diet and subsistence practices. The earliest Mesolithic arrivals on what we presume were dugout canoes, date to a time when Malta had almost reached its current configuration, which today has a minimum straight-line distance of around 85 km to Sicily^34,35^. However, sea surface currents and prevailing winds, as well as the use of landmarks, stars and other wayfinding practices, mean that the distances traversed by hunter-gatherers to Malta could have been considerably longer, and a crossing of about 100 km has been proposed for the Neolithic^36–39^ (Supplementary Information 1). In particular, any crossing from Sicily to Malta would have had to contend with the ocean current dynamics in the Malta Channel^40^. Experimental voyages on a replica of an Early Neolithic dug-out canoe from La Marmotta (Italy) suggest that crossings of 50 km could be accomplished at a speed of about 4 km h^−1^ (just over 2 knots)^41^, implying an outward summer sea journey that would have necessitated all daylight hours and an additional 8 h of darkness. In the summer, the drift caused by a southeasterly current that goes up to as much as 2 knots would have extended this outward journey even further^42^. In antiquity, as well as more historic periods, these conditions seem to have led sailboats to prioritize ports along the Gulf of Gela as a point of departure from Sicily, rather than the closest point to Malta^43^. These findings therefore provide evidence of long-distance, open-water sea journeys that were far longer than any previously documented in the Mediterranean, before the Neolithic and Bronze Age, when developments such as the invention of the sail occurred^1^. Such inter-island crossings fall into the category of ‘difficult routes’; evidence from elsewhere suggests that canoers would avoid the dangers of voyaging at night altogether^44^.

The motivation for these long sea crossings remains ambiguous. It might be that movement to Malta was driven by the availability of (perhaps seasonal) subsistence resources, catalysed by the slightly improved climate of the Early Holocene. It is also possible that the Maltese Mesolithic reflects social rather than environmental factors; namely, the potential regional demographic shockwaves through hunter-gatherer societies associated with the transition to the Neolithic in the Mediterranean (Supplementary Information 1).

The story of Mesolithic Malta is part of a set of broader demographic and behavioural changes in the dynamic epoch of the final hunter-gatherer societies of the Mediterranean. These are both important in their own right, and also set the cultural and ecological scene for the transition to the Neolithic. The ability of Mesolithic hunter-gatherers to reach small and remote Mediterranean islands forces a re-evaluation of the capabilities and strategies of the last hunter-gatherers of the region. It also shows that Neolithic arrivals did not enter a pristine insular landscape on Malta, but rather an ecosystem that had been shaped by humans for centuries. Finally, the presence of Mesolithic hunter-gatherers on Malta raises the possibility of other long-distance connections. For example, the technological similarities between contemporary Mesolithic and Epipaleolithic communities on the African and European sides of the Mediterranean have been noted^45–47^. The combination of several islands, and their proximity to indented mainland shorelines, has also suggested that the south-central Mediterranean and eastern Maghreb could have been a hub for early maritime activity in the region^48^. The evidence we present for early long-distance seafaring from the heart of the Mediterranean adds another layer to this emerging narrative.

## Methods

### Overview

**Extended Data Fig. 4:**
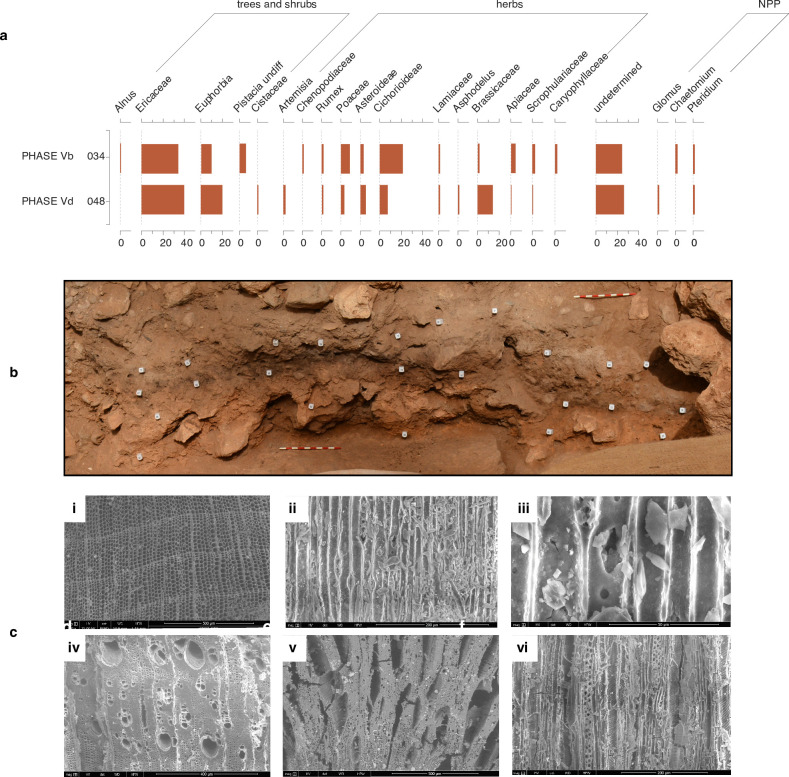
Results of archaeobotanical analyses. **a**, Pollen diagram from Latnija archaeological site, with percentage values of pollen remains identified from contexts (034) and (048). **b**, The deep Mesolithic hearth from Phase V, square N2, with sample locations for phytolith and FTIR studies. The internal structure of the hearth can be observed, from top to bottom, combustion residue, thermal impact and natural substrate (control). **c**, ESEM images of *Pistacia* cf. *lentiscus* and *Juniperus* sp. charcoal remains showing wood anatomical characters: (i) *Juniperus* sp. charcoal fragment tangential section; (ii) *Juniperus* sp. charcoal fragment tangential section showing rays and tracheids; (iii) *Juniperus* sp. charcoal fragment tangential section showing a detail of tracheid pits; (iv) *Pistacia* cf. *lentiscus* charcoal fragment transverse section showing ring porous distribution and vessel clusters; (v) *Pistacia* cf. *lentiscus* charcoal fragment tangential section showing spiral thickenings and biseriated rays; (vi) *Pistacia* sp. charcoal fragment transverse section showing cracks and vitrification altering the wood cell structure.

**Extended Data Fig. 5:**
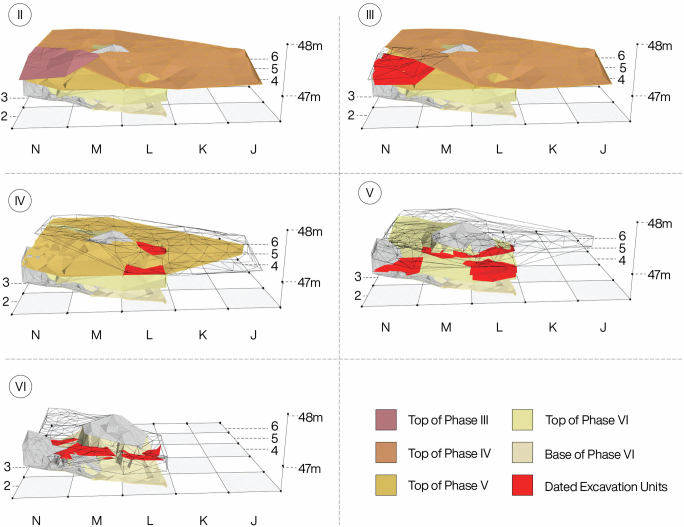
Chronological sample locations. Oblique view illustrating the location of dated contexts (red) with respect to sediment phase boundaries spanning grid squares J–N, showing the distribution of sediments at the base of Phase II**;** the location of dated contexts from Phase III, with the upper boundary of Phase III deposits shown as a wireframe; the location of dated contexts from Phase IV, with the upper boundary of Phase IV deposits shown as a wireframe; the location of dated contexts from Phase V, with the upper boundary of Phase V deposits shown as a wireframe; and the location of dated contexts from Phase VI, with the upper boundary of Phase VI deposits shown as a wireframe.

**Extended Data Fig. 6:**
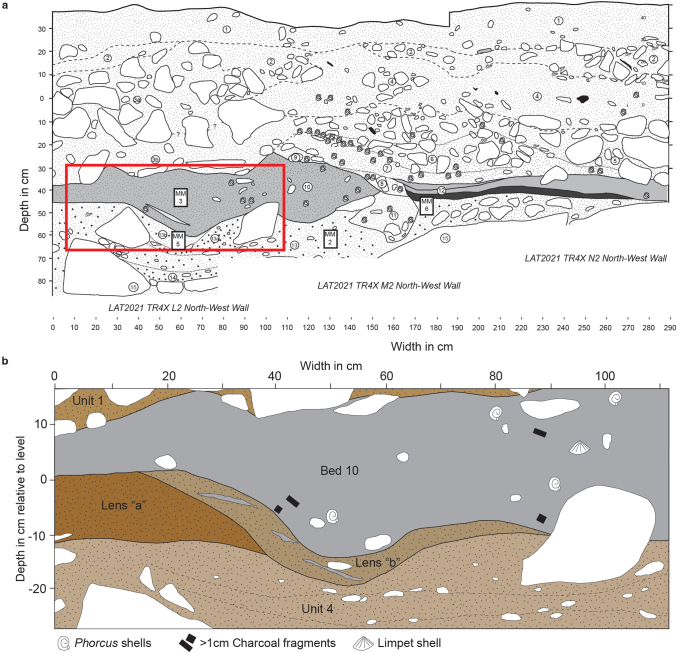
Section drawing. **a**, Section drawing of the Latnija exposure with the detailed section shown in Fig. 3 highlighted (recorded September 2022). **b**, Detailed record of the contact between the upper part of Unit 4 and the lower contact of Bed 10 (for location of the recorded section see Fig. 1). The section was recorded in September 2024, note that some of the large clasts that were recorded in the September 2022 section (**a**) had been removed from the section by September 2024.

**Extended Data Fig. 7:**
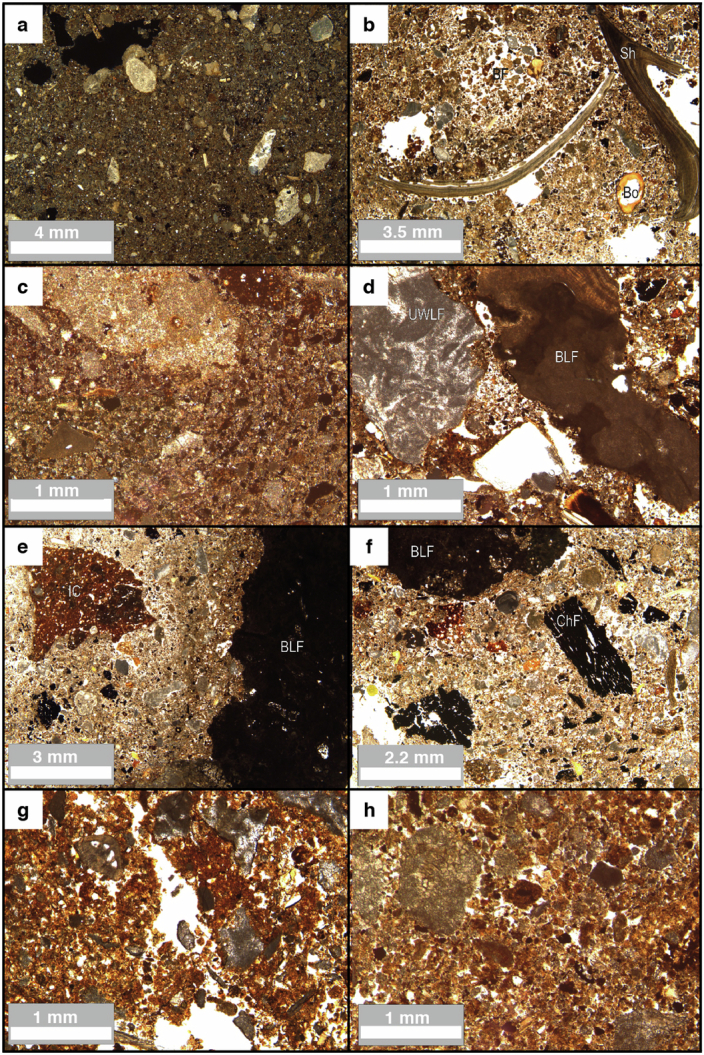
Photomicrographs of the Latnija sediments. **a**, Overview of the sediments of Unit 1. The sediments are dominated by limestone derived clasts and fine-grained material. The clear grains are fine-sand/silt sized grains of quartz. **b**, Biological material within Unit 1 (Sh – shell, Bo – Bone, BF – Burrow fill). **c**, Overview of the sediments of Unit 2 (Bed 10). The high birefringence colours show the dominance of limestone material in both the coarse and fine component. **d**, Limestone clasts within Bed 10 showing unweathered limestone fragments (UWLF) and blackened, burnt limestone fragments (BLF). **e**, Burnt limestone fragment (BLF) next to an iron oxide enriched intraclast (IC) of reworked sediment. **f**, Charcoal Fragment (ChF) and Burnt limestone fragment (BLF) in Bed 10. **g**, Iron enriched sediments of Unit 1 from directly below the contact with Bed 12. **h**, Pelleted microfabric of lens b below Bed 10.

**Extended Data Fig. 8:**
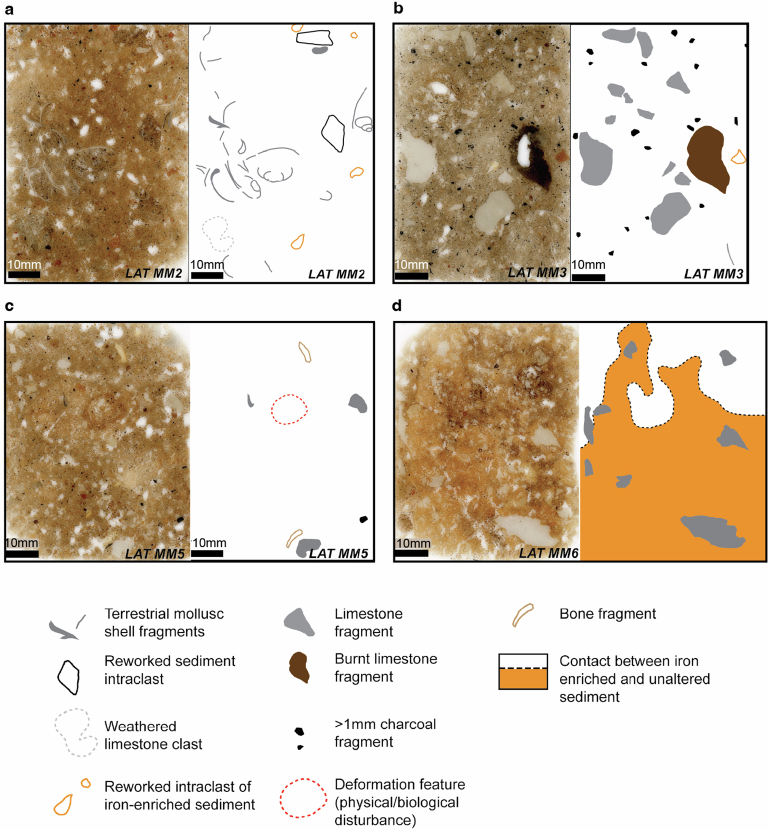
High-resolution scans of micromorphological thin sections from the Latnija sequence. See Extended Data Fig. 6 and sediment section (Supplementary Information 2) for the location of thin-section samples. **a**, MM2 is taken from the unaltered sediments at the base of the sequence. This sample presents the key characteristics of patterns of sedimentation prior to the Mesolithic occupation. These can be summarized as consisting of: 1) rare limestones clasts (frequently showing evidence for in situ decay), 2) intraclasts of reworked sediment and 3) fragments of terrestrial mollusc shell. Limited materials that are indicative of burning or burning products are present. **b**, MM3 is taken from the ash-rich sediments of the sequence, a factor that can be seen in the colour difference between the brown matrix of MM2 and the grey matrix of MM3. MM3 is rich in >1 mm sized charcoal fragments and limestone clasts, some of which show evidence for a strong degree of burning. **c**, MM5 is taken from the contact between the ash-rich sediment (MM3) and the underlying cave floor sediment (MM2). The sediment of MM5 is, consequently, characterized by a mixture of both sediment types. Charcoal fragments are abundant (but only rarely >1 mm) but the matrix overall is more typical of MM2. Bones fragments are present as are circular features that are characteristics of deformation which could be either biological or physical in origin. The sediments have formed in association with cutting into pre-existing sediments prior to the deposition of the ash-rich sediments. **d**, MM6 is taken from below darkened sediment believed to be in situ burning. The sediments of MM6 are more reddened than any other sampled sediments and occur directly below sediments the colour of which are more typical of the unaltered cave sediments of MM2. This unit is interpreted as being thermally altered as a direct result of in situ burning directly above these deposits.

**Extended Data Fig. 9:**
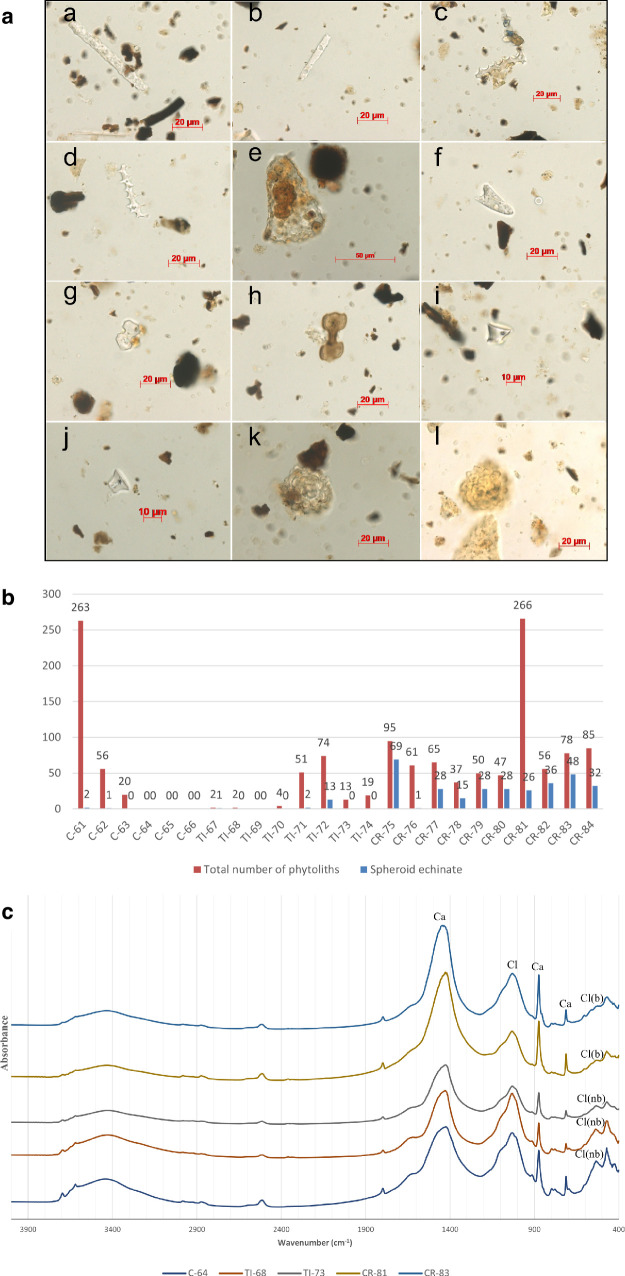
Phytolith study. **a**, Phytoliths image identified in the Phase V deep hearth. a,b, Elongate entire from grass leaf; c,d, Elongate dentate from grass inflorescence; e, Bulliform flabellate from grass leaf; f, Acute bulbosus from grasses leaf; g,h, GSSCP Bilobate from grasses-C4; i–j, GSSCP Rondel from grasses-C3; k,l, Spheroid echinate from Palms. **b**, Different dynamics of phytoliths identified in the layers that make up the hearth in relation to the number of phytoliths identified as spheroid echinate in each sample. Key: C, Control; TI, Thermal Impact; CR, Combustion Residue. **c**, Infrared spectra of sediments from some representative samples. Key: C, Control; TI, Thermal Impact; CR, Combustion Residue; Ca, Calcite; Cl, Clay; Qz, quartz; b, thermally altered clay; nb, not thermally altered clay.

**Extended Data Fig. 10:**
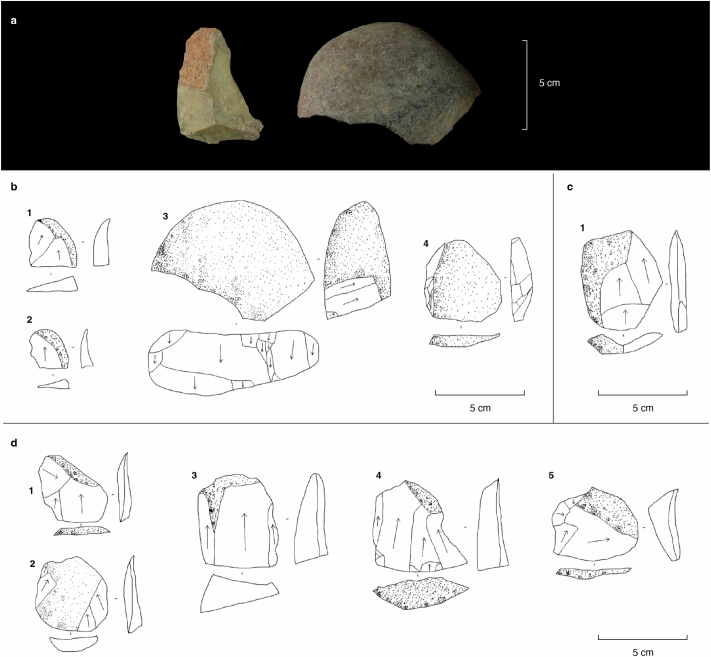
Lithics from the Maltese Mesolithic. **a**, Photographs of lithics from Phase III (left) and Phase V (right) showing terrestrial and coastal raw material forms. **b**–**d**, Illustrations of lithics from Phases V (**b**), IV (**c**) and III (**d**). All are flakes except b3 (core) and b4 (retouched flake). All limestone.

**Extended Data Fig. 11:**
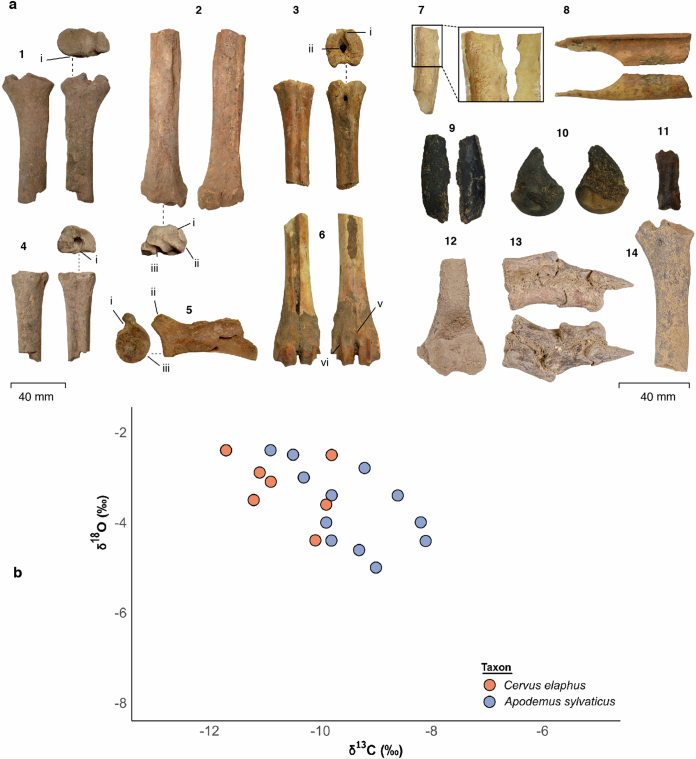
Taxonomic and isotopic analyses of faunal remains. Red deer remains, taphonomic modifications, and stable isotope results. **a**, Remains of red deer including a proximal radius (1), distal radius (b), proximal metatarsal (3), proximal metacarpal (4), scapula (5), and a distal metatarsal (6). Examples of taphonomic modifications including a midshaft fragment with a green fracture and a double notch with corresponding negative flake scars (7), a midshaft fragment with a green fracture (8), examples of charred bone (9–11), and examples of bone covered in adhering matrix (12–14). **b**, Results of the stable carbon δ^13^C and δ^18^O analysis by taxa. Roman numerals refer to ref. ^75^. Left scale bar applies to A1–6 and right scale bar to A7–14.

Our multidisciplinary study combines archaeobotany (Supplementary Table 1), chronological modelling (Supplementary Tables 2–6), isotopes (Supplementary Table 7), contextual research and broad regional chronological modelling (Supplementary Tables 8 and 9) with anthracology and phytoliths (Supplementary Tables 10–13), lithics (Supplementary Table 14) and the study of faunal remains (Supplementary Tables 15 and 16). The methods used are described below, with further contextual information in the Supplementary Information and Extended Data Figs. 1–11.

### Excavation and sedimentology

Here, we describe the excavation of a 5 × 5-m trench, designated Trench 4, at Latnija between 2021 and 2023, expanding on a 1 ×1-m test trench excavated in 2019. We set up an alphanumeric grid system in the doline to label each individual 1 ×1-m square, aligned in orientation with the 2019 test trench and with the nearby cave wall, with letters running on a SW–NE axis and numbers increasing on a NW–SE axis. The 2019 test excavation targeted square M2, with the expanded Trench 4 spanning squares J–N and 2–6, located at the northern edge of the doline spanning the dripline (Fig. 1).

Excavation was performed using a single-context recording methodology to resolve between discrete sediment units, with arbitrary subdivisions within a single deposit as 5–10-cm spits where necessary to aid control of find recovery and sediment sampling. Features of post-depositional disturbance, such as animal burrows, were readily differentiated from undisturbed sediments owing to their mixed character and friable texture and the presence of sediment voids, and were excavated in their entirety and excluded from our analyses. Finer-scale post-depositional disturbance occurs as limited fine rooting and is restricted to the uppermost deposits. The natural deposition of clasts from the shelter wall presents an alternate form of potential post-depositional disturbance that might have led to localized soft-sediment deformation. The three-dimensional position of all artefacts larger than 20 mm, bones larger than 20 mm and charcoal, and the geometry of excavation context boundaries, were recorded using a total station. Bulk sediment sampling retained a minimum of 60 l per context (predominantly in the uppermost deposits) up to 100% sampling of sediments, which were processed by bucket flotation using 250-µm mesh for macrobotanical recovery, followed by wet sieving through 5-mm screens for artefact recovery; sediments that were not retained for flotation and wet sieving were dry sieved through 5-mm screens. Additional sediment samples were recovered from each context for ancillary analyses.

So far, we have identified 309 discrete sedimentary contexts, reaching a maximum depth of 1.48 m from the surface. We have grouped contexts into six phases (Phases I to VI) on the basis of major changes in sediment colour, texture, composition and structure, alongside patterns evident in material culture. The stratigraphic matrix for the Mesolithic Horizon and immediately underlying deposits is presented in Extended Data Fig. 3.

Micromorphological samples for thin-section production were collected by cutting in situ, orientated blocks of sediment into Kubiena tins (90 mm × 70 mm × 50 mm). The location of Kubiena samples was dictated by the architecture of the sediment sequence and representative sediment deposits, and the contacts between deposits were targeted. The laboratory samples were air-dried for two weeks and placed in labelled plastic pots. The samples were immersed in a mixture of clear casting resin (four parts) to acetone (one part). To accelerate curing, a catalyst of methylethylketone peroxide was added (3 ml catalyst to 2,000 ml resin). The resin mixture was poured around the side of the sample to allow the larger pore spaces to be filled from adhesion and cohesion, and then completely immersed in the resin. The samples were impregnated under a stepped-vacuum regime to a maximum vacuum pressure of −25 in Hg for eight hours. The samples were left to cure for around six weeks until the resin was hardened, followed by a final cure at 65 °C for 15 h. The blocks were removed from the sample frame, split along their long axes and one surface polished on fixed diamond abrasives with successively finer grades (70 µm, 45 µm and 20 µm). The polished sample was stuck to a labelled slide using an epoxy resin that cures overnight. The slide and sample were cut down to around 1 mm and then excess sample was removed using a Jones and Shipman surface grinder. The sample was hand-polished to finish off the surface before coverslipping the sample again by bonding with an epoxy resin. Analysis of the thin sections was performed on a Leica M205C petrological stereo zoom microscope and image capture was done using the Image Pro-Express software.

### Archaeobotanical methods

Studies of plants in the Mesolithic Horizon at Latnija were performed in the form of pollen analyses, anthracology, hearth phytolith analyses and macrobotanical identifications from remains recovered through flotation. These analyses were performed to reconstruct the vegetation of the site, determine whether any domesticated plants were present, investigate the use of different fuels at the site and unravel mineral composition to identify combustion structures.

For pollen analysis, we collected sedimentary samples to perform palynological analyses focused on the reconstruction of past vegetation at and near Latnija. Sampling was performed in Phase V contexts (034) and (048), both of which are characterized by the presence of thick ash and combustion residue deposits. This approach was adopted to correlate the palaeobotanical remains preserved in the sediment with human activities during Phase V, which is characterized by the oldest Mesolithic.

Samples were treated following pollen concentration techniques^52^. This included sediment deflocculation with sodium pyrophosphate, Lycopodium tablets with known content to calculate palynomorph concentration values^53^ and 7-µm nylon sieve to discard clay-sized particles. Carbonates were removed with 10% HCl and concentrated at 2,500 rpm for three minutes. Heavy liquid separation using sodium metatungstate with a specific gravity of 2.0 and centrifugation at 1,500 rpm for 20 min was done to separate organic and mineral fractions. After recovering the upper supernatant fraction, this step was repeated to increase the concentration. The remaining fraction was treated with cold 40% HF for one night to eliminate remaining silicates. The residue was washed in 98% ethanol, glycerol was added and the remaining ethanol was evaporated. The solution was kept in glycerol, mounted on slides and identified at 400× magnification under a light-transmitted microscope by referring to established literature^54,55^. Pollen counts were done up to 250 identifiable grains. A pollen diagram (Extended Data Fig. 4a) indicating values for each taxon as percentages of the total pollen sum was plotted with the help of C2 software^56^.

For anthracological analyses, bucket flotation was used to recover charcoal and other carbonized archaeobotanical remains from the sediments, all of which were collected. Charcoal was also handpicked to provide a larger number to select for dating purposes and anthracology.

A total of 165 charcoal fragments were observed under reflected light microscopy (Motic PANTHERA) with dark and bright fields and ×50, ×100, ×200 and ×500 magnifications. Images were taken with an environmental scanning electron microscope (FEI Quanta 600) coating charcoal with gold. Each charcoal piece was manually fragmented into the three wood anatomy sections (transverse, tangential and radial). Observing the three anatomy sections allowed us to identify taxonomic characters. Different wood anatomy atlases and a comparative collection at the Catalan Institute of Human Paleoecology and Social Evolution were used to support the identifications^57,58^. The assemblage is characterized by a number of indeterminable fragments related to wood anatomy alterations (cracks and vitrification) and/or size of the fragments.

To study the pyroarchaeological record of the Mesolithic Horizon at Latnija, we combined the study of phytoliths and the mineralogical composition of sediments by FTIR. We analysed 24 samples that were collected during the 2022 fieldwork from a large combustion structure identified in square N2 at the base of Phase V (Fig. 2). Sampling was performed on the basis of visual identification of the internal structure of the hearth, distinguishing between samples coming from the possible combustion residue (*n* = 10), samples coming from the thermal impact zone (*n* = 8) and control samples from below the hearth (*n* = 6).

Phytoliths were extracted following the fast extraction method^59^. Phytolith quantification and identification was done using a Zeiss Axioscope transmitted light microscope at ×200 and ×400 magnifications. Phytolith morphological identification followed the standard literature and modern plant reference collections^33,60–62^. We followed the terminology of the International Code for Phytolith Nomenclature (ICPN 2.0) for phytolith descriptions^63^.

The mineral composition of the samples was identified using a Jasco FT/IR-6700 spectrometer. Infrared spectra were collected in the 4,000–400 cm^−1^ wavelength range at a resolution of 4 cm^−1^ using the conventional KBr pellets method. The spectra were interpreted using the position of the main peaks described on reference collections^64^. Thermally altered clay was identified on the basis of specific absorption peaks in the clay spectrum^65^, and the presence of anthropogenic or geogenic calcite was determined following previous studies^66,67^.

The archaeobotanical samples from Latnija’s Mesolithic Horizon were recovered from the 2021 and 2022 excavation seasons. Although we engaged in a 100% sediment collection strategy, after flotation, not all samples from these phases contained plant macrofossils. The assemblage suitable for study consists of 28 samples in total—19 from the 2021 field season and 9 from the 2022 season. Each sample was processed in the field using a basic bucket flotation method, as described previously^68,69^. The samples were then sent to the Max Planck Institute of Geoanthropology in Jena, Germany, for analysis. Once in the laboratory, samples were passed through nested U.S. Geological sieves to ease sorting. Material smaller than 0.50 mm was not sorted. Carbonized wood fragments larger than 2 mm were counted, although wood identification was done as a separate analysis and is reported above. Seeds and seed fragments were separated from all sieved contexts, and charred seeds were systematically collected. The identified taxa are presented in Supplementary Table 1.

### Radiocarbon dating methods

Except for the bone samples, radiocarbon dating was performed at the Curt-Engelhorn-Centre Archaeometry (CEZA) in Mannheim, Germany. Samples included charcoal, seeds and marine shells. Bone samples were analysed at the University of Georgia Centre for Applied Isotope Studies (CAIS). We used a multistep chronological study to clearly constrain the Mesolithic Horizon at Latnija. First, we constructed a chronological framework for the site, which involved 31 charcoal samples and the one bone (Supplementary Tables 2 and 3). Charcoal samples were selected from contexts directly underlying the Mesolithic Horizon to help constrain the onset of Mesolithic occupation, excluding samples from burrows that appear at the interface of major divides in sediment depositional processes (Phases VI–V) (see Extended Data Fig. 5 for illustrated sample locations). In addition, charcoal samples were selected from contexts throughout the Mesolithic Horizon (Phases V–III), including direct sampling from hearths that appear at the base of Phase V (Fig. 2, Supplementary Information 2 and Extended Data Fig. 6). The model was divided into the major phases recorded during the excavation (Supplementary Information 2).

To obtain independent verification of the integrity of the age model, we also targeted marine gastropods (*P. turbinatus* in particular) because they formed clear in situ tip lines identified in Phase III. Forty-nine samples of *P. turbinatus* were dated for this purpose. The number of samples was chosen to reflect the fact that: (i) marine calibration is more complex than terrestrial calibration, thus a larger sample size was required to account for the natural spread in the data; and (ii) these shells are a direct measure of human presence, because they have been imported to the site by people.

Charcoal samples were prepared using a standard ABA pretreatment. This covers an acid step with diluted hydrochloric acid to remove calcite and lime attached to the sample. A base step with diluted sodium hydroxite follows to remove soluble humic acids. As the base attracts fresh CO_2_, another acid step finalizes the pretreatment and removes any modern contamination. The samples are then combusted in an elemental analyser (MicroCube, Elementar) and the CO_2_ is collected and graphitized to elemental carbon. The carbon is pressed into a target and measured in a MICADAS mass spectrometer^70^.

The shell samples only undergo a treatment with diluted acid to remove adjacent carbon contamination from limestone or calcite. For shell samples, the CO_2_ is extracted using phosphoric acid in an autosampler before graphitization, and measurements are the same as for the charcoal samples described in a previous study^71^.

The bone sample was cleaned by wire brush and washed using an ultrasonic bath. After cleaning, the sample was then reacted under vacuum with 1 M HCl to dissolve the bone mineral and release CO_2_ from bioapatite. The residue was filtered, rinsed with deionized water and, under slightly acid conditions (pH 3), heated at 80 °C for six hours to dissolve collagen and leave humic substances in the precipitate. The collagen solution was then filtered to isolate pure collagen and dried out. The dried collagen was combusted at 575 °C in evacuated and sealed Pyrex ampoules in the present CuO. The resulting CO_2_ was cryogenically purified from the other reaction products and catalytically converted to graphite. Graphite ^14^C/^13^C ratios were measured using the CAIS 0.5 MeV accelerator mass spectrometer. The sample ratios were compared with the ratio measured from the Oxalic Acid I (NBS SRM 4990). The uncalibrated dates were then given in radiocarbon years before 1950 (years bp), using the ^14^C half-life of 5,568 years. The error is quoted as one standard deviation and reflects both statistical and experimental errors. The date has been corrected for isotope fractionation. As with other terrestrial radiocarbon dates in this study, calibration was performed with OxCal 4.4 using IntCal20 and as part of a phase model for the site. Modelled and unmodelled calibrated dates and model diagnostics are presented in Supplementary Tables 2 and 3.

To correct for the MRE, we compared the ages of *P. turbinatus* shells with the ages of charcoal from the same stratigraphic contexts. The reservoir effect Δ*R* was modelled in OxCal 4.4. with the latest datasets of IntCal20 for the charcoal samples and Marine20 for the shells. It was modelled using a phase model and choosing a wide restriction for Δ*R*. Samples marked by OxCal as outliers are presented in the table but are not included in the next modelling step if the model cannot deal with them leading to an *A* of less than 60%. These outliers might reflect processes such as bioturbation. The results of the MRE calculations are shown in Supplementary Table 4, and the corrected dates for each *P. turbinatus* age are shown in Supplementary Table 5 (see also Supplementary Information 4).

### Radiocarbon modelling methods

Models involving radiocarbon dates were used to address the key question of whether there is evidence of occupation in the Latnija cave excavation sequence that securely relates to human activity predating the available evidence for Neolithic habitation elsewhere on Malta and in the surrounding Mediterranean archaeological record. This was done by: (1) establishing the age of the Mesolithic deposits at Latnija; (2) determining when the wider regional Mesolithic-to-Neolithic transition is most likely to have occurred; and (3) determining whether there is evidence for an early Neolithic occupation of Malta in in a sediment core extracted from Salina Bay in northeast Malta, while accounting for the high-energy depositional environment and chronological uncertainty associated with radiocarbon dates used to produce associated age–depth models. Each of the analyses was conducted in R and is fully replicable, with scripts, data and outputs contained in a GitHub repository along with further replication instructions (https://github.com/wccarleton/mesoneomalta).

First, we used a standard archaeological phase model to determine start and end boundaries for major depositional phases identified at Latnija. For this model, the excavation team constructed a general Harris matrix relating different contexts to major phases of sediment deposition and artefact accumulation. Thirty-three radiocarbon samples—charcoal from short-lived local shrubs and one bone—recovered from these units were then dated and the dates were placed into an OxCal phase model to estimate phase boundary distributions. All phase boundaries were of the ‘sigma’ type. This boundary allows the tails of the distribution of events (dates) making up abutting phases to overlap. The flexibility reflects the sedimentary fuzziness inherent in the physical boundaries between depositional units. Following previously published guidance^72^, we included a general outlier model along with the phases, allowing for the model to identify potential outliers (events with extreme dates relative to both their phases and the structure of the model as a whole). The modelling identified no significant outliers among the radiocarbon-dated samples given the boundaries we used, as indicated by the posterior probabilities associated with the outlier model that indicate the probability that a given sample is an outlier in the model context (all were 8% or less; most were 4%; Supplementary Table 6).

Next, we used a cleaned regional database of radiocarbon dates associated with securely identified Mesolithic and Neolithic sites or site components from Italy, Sicily, Corsica, Sardinia and Malta. We divided the dates by region and cultural association. Then, we used a simple OxCal phase model to estimate when the Mesolithic phase ended and the Neolithic phase began in each of the regions (details in Supplementary Information 4). We used both ‘sigma’ and ‘uniform’ boundaries to model the end of the Mesolithic and the start of the Neolithic. The former allows overlap between the phases, while the latter does not, and given available data both perspectives may be valuable. By reporting both we therefore parameterise the most likely range of scenarios.

Finally, we re-examined the published age–depth model for the Salina Deep sediment core. The core was argued to contain evidence for an early Neolithic in Malta, because it contains findings such as the pollen of domesticated cereals, which was estimated to date to around 8 ka on the basis of an age–depth model. However, the age–depth model used (Bchron), like many sophisticated sedimentation models, assumes monotonicity in the age–depth relationship, which we argue does not apply in the Salina Deep case. Although monotonicity is typically a good working assumption in low-energy depositional environments without evidence of disturbance, Salina Bay in the past and present is a high-energy littoral and fluvial environment that is subject to frequent storms. The core itself contains evidence of marine ingression and many of the radiocarbon dates indicate substantial sediment redeposition, with very old dates near the surface and segments showing a wide radiocarbon temporal spread. Together, this evidence suggests that monotonicity is a poor assumption for Salina Deep and, consequently, that the published age–depth model is overly (unduly) precise because it cannot account for the wide variance in radiocarbon sample dates for many of the core’s segments. To account for this, and produce a model that is more representative of the empirical temporal variance, we used a linear Bayesian regression to model the age–depth relationship. The model recognizes a general relationship in the available age–depth observations indicating a trend toward older dates correlated with depth. However, it also does not assume strict monotonicity, instead focusing on the broad age–depth relationship. We used a custom distribution (based on standard radiocarbon-date calibration) to add a measurement uncertainty component to the model, representing radiocarbon dating and calibration uncertainties. We also used Bayesian imputation to model dates with full posterior uncertainty for a sequence of undated sediment depths (see Supplementary Information 1 for further details).

### Zooarchaeological methods

During the 2021 and 2022 field seasons, faunal remains greater than 20 mm in length were piece plotted using a total station, given a unique identifier and bagged. Smaller bone fragments, shells and other faunal remains were recovered through various methods, including an exhaustive programme of wet sieving, flotation and manual inspection of 8-mm, 4-mm, 2-mm, 1-mm and 0.5-mm sieved sinks under microscopy. Here we present a preliminary taxonomic and taphonomic analysis of this faunal material, but note that a full detailed analysis is currently underway that comprises all remains recovered during excavation.

Bones were identified to skeletal element and, for the most part, to broad taxonomic categories (for example, fish and birds), facilitated by relevant literature^73–76^, online resources and comparative material housed at the University of Malta. The taphonomic analysis focused on identifying bone fractures and surface modifications, such as burning, butchery marks (such as cut marks) and carnivore damage (for example, gnawing) following standard protocols^77–80^. Remains are reported as the number of specimens (NRSP) and number of identified specimens (NISP), following a previous report^81^. NRSP includes all skeletal remains (bones and teeth) included in this study, whereas NISP is defined as all skeletal elements (bones and teeth) identified minimally to class.

In addition to the piece-plotted bone, we also report here the complete counts of marine fauna for two excavation squares (L2 and N2), reflecting material that was directly recovered and bagged during excavation and material from wet sieving and flotation. Given the very different sediment volumes exposed for the different phases, we chose here to focus at first on these two squares, which offer a good sequence through the phases, to showcase the marine component at the site.

### Isotope methods

Nineteen samples, comprising 12 wood mouse (*Apodemus sylvaticus*) and 7 red deer (*Cervus elaphus*), were selected for δ^13^C and δ^18^O isotope analysis of tooth enamel (Supplementary Table 7). For red deer, molar teeth were targeted for analysis, although the sample set does include one red deer premolar tooth. It should also be noted that because some of these samples are non-overlapping teeth, it is possible that some pseudo-sampling (sampling from the same individual) took place. For wood mouse, whole molar and incisor teeth were used to ensure that the minimum sample size for stable isotope analysis was met.

Before sampling, red deer were cleaned through gentle abrasion with a diamond-tipped drill to remove any adhering material. After cleaning, the same approach was used to sample the tooth enamel along the full length of the buccal surface to ensure a representative measurement for the period of tooth formation. For wood mouse, as much of the dentine was removed as possible using a drill before the remaining whole teeth were crushed using a mortar and pestle, with cleaning of the mortar and pestle using 70% ethanol between samples.

To remove organic or secondary carbonate contaminates, all samples underwent pretreatment, which involved soaking in 0.1 M acetic acid for 10 min followed by three rinses in purified water^82,83^. After reaction with 100% phosphoric acid, gasses were analysed using a Thermo GasBench II connected to a Thermo Delta V Advantage mass spectrometer housed at the Department of Archaeology at the Max Planck Institute of Geoanthropology. Carbon and oxygen isotopes are reported as the ratio of heavier to lighter isotopes (^13^C/^12^C or ^18^O/^16^O) in parts per million (‰) relative to international standards (Vienna Peedee Belemnite, VPDB). δ^13^C and δ^18^O values were normalized using a three-point calibration against the international standards IAEA-603 (δ^13^C = 2.5‰, δ^18^O = −2.4‰), IAEA-CO-8 (δ^13^C = −5.8‰, δ^18^O = −22.7‰) and IAEA NBS 18 (δ^13^C = −5.014‰, δ^18^O = 23.2‰), as well as the in-house standard of USGS44 (δ^13^C = −42.2‰).

### Reporting summary

Further information on research design is available in the Nature Portfolio Reporting Summary linked to this article.

## Online content

Any methods, additional references, Nature Portfolio reporting summaries, source data, extended data, supplementary information, acknowledgements, peer review information; details of author contributions and competing interests; and statements of data and code availability are available at 10.1038/s41586-025-08780-y.

## Supplementary information

Supplementary Information This document contains additional details and information including the context of research (1), a description of the deposits (2), details of the archaeobotany (3), a chronology (4), lithic analyses (5), faunal analyses (6), Supplementary Tables 1–16 (7), OxCal scripts (8) and Supplementary References (9).

Reporting Summary

## Data Availability

Data required to reproduce the chronological models are available at https://github.com/wccarleton/mesoneomalta, and are archived with Zenodo at 10.5281/zenodo.14192393 (ref. ^84^).

## Code availability

Code required for reproducing the chronological models is available at https://github.com/wccarleton/mesoneomalta, and is archived with Zenodo at 10.5281/zenodo.14192393 (ref. ^84^).
