# Author Correction: Hunter-gatherer sea voyages extended to remotest Mediterranean islands

**DOI:** 10.1038/s41586-025-10024-y

**Published:** 2026-01-29

**Authors:** Eleanor M. L. Scerri, James Blinkhorn, Huw S. Groucutt, Mathew Stewart, Ian Candy, Ethel Allué, Aitor Burguet-Coca, Andrés Currás, W. Christopher Carleton, Susanne Lindauer, Robert Spengler, Kseniia Boxleitner, Gillian Asciak, Margherita Colucci, Ritienne Gauci, Amy Hatton, Johanna Kutowsky, Andreas Maier, Mario Mata-González, Nicolette Mifsud, Khady Niang, Patrick Roberts, Joshua de Giorgio, Rochelle Xerri, Nicholas C. Vella

**Affiliations:** 1 Human Palaeosystems Group, Max Planck Institute of Geoanthropology, Jena, Germany; 2 Department of Classics and Archaeology, Faculty of Arts, University of Malta, Msida, Malta; 3 Institute of Prehistoric Archaeology, University of Cologne, Cologne, Germany; 4 Department of Archaeology, Classics and Egyptology, University of Liverpool, Liverpool, UK; 5 Australian Research Centre for Human Evolution, Griffith University, Brisbane, Queensland Australia; 6 Department of Geography, Royal Holloway University of London, Egham, UK; 7 Institut Català de Paleoecologia Humana i Evolució Social (IPHES-CERCA), Tarragona, Spain; 8 Departament d’Història i Història de l’Art, Universitat Rovira i Virgili (URV), Tarragona, Spain; 9 Department of Archaeological Sciences, Faculty of Archaeology, Leiden University, Leiden, The Netherlands; 10 Department of Land Use and Urbanisation, Max Planck Institute of Geoanthropology, Jena, Germany; 11 Curt-Engelhorn-Centre Archaeometry, Mannheim, Germany; 12 Domestication and Anthropogenic Evolution Research Group, Max Planck Institute of Geoanthropology, Jena, Germany; 13 Superintendence of Cultural Heritage, Valletta, Malta; 14 Evolutionary Ecology Group, Department of Zoology, University of Cambridge, Cambridge, UK; 15 Department of Geography, Faculty of Arts, University of Malta, Msida, Malta; 16 Department of Structural Changes of the Technosphere, Max Planck Institute of Geoanthropology, Jena, Germany; 17 Institute for Archaeological Sciences, University of Tübingen, Tübingen, Germany; 18 Département d’Histoire, Université Cheikh Anta Diop de Dakar, Dakar, Senegal; 19 isoTROPIC Research Group, Max Planck Institute of Geoanthropology, Jena, Germany; 20 Department of Archaeology, University of York, York, UK; 21 National Museum of Natural History, Heritage Malta, Mdina, Malta

**Keywords:** Archaeology, Palaeoecology

Correction to: *Nature* 10.1038/s41586-025-08780-y Published online 9 April 2025

Since the version of the article initially published, two corrections have been made, which do not have a meaningful impact on our results. The errors referred to one specific element of our study, the regional model of the timing of the Mesolithic to Neolithic transition which was discussed in the Supplementary Information and presented in Extended Data Fig. 1. While we consider it scientifically prudent to issue a correction, we emphasise that the results of this specific part of our analysis (in essence, a summary of radiocarbon dates from the central Mediterranean) are virtually the same as previously published. Our results mirrored, and still mirror, the general consensus on the timing of the Neolithic transition in the region.

The first correction involved correcting the uncertainties (errors) of radiocarbon dates in the regional radiocarbon date database used for the phase models of the Mesolithic and Neolithic in the central Mediterranean, where the error from one radiocarbon date had been incorrectly repeated for a number of others. The errors have been updated where necessary.

The second correction involved the boundaries in the regional Mesolithic-to-Neolithic phase models. We had intended to use OxCal’s ‘sigma’ boundaries throughout, but we had actually only used sigma boundaries for the Mesolithic of Northern Italy; the boundaries for the remaining phases and regions were of the ‘uniform’ type. Since debates could be had regarding which are the most appropriate theoretically, we decided we should present both as part of this correction (see Supplementary Fig. 1), rather than simply changing the unintentional ‘uniform’ types to ‘sigma’ types. To reduce the likelihood of this error, we edited the R script for the analysis to include a section fully automating the production of the OxCal scripts (the ‘.oxcal’ files).

Neither correction makes a material difference to our interpretations (see Supplementary Tables 1 and 2). The corrected uncertainties reduced the variance of the modelled boundary posteriors because many of the correct date errors were lower than the incorrect value we used. Furthermore, using either type of boundary, ‘uniform’ or ‘sigma’ types, made little material difference to the posterior distributions for the starting boundaries of the Neolithic phases across regions. In line with expectations based on what these boundaries actually refer to (see Fig. 4 in ref. ^1^), the ‘sigma’ boundaries for each phase are closer together, because the underlying distribution of events is assumed to cluster more than with the uniform boundaries, and because the boundaries themselves refer to an interval within the absolute earliest and latest dates within a given phase. In every region, the estimated start date distributions for the Neolithic phases are slightly later in time under the assumption of ‘sigma’ boundaries than under the assumption of ‘uniform’ boundaries, which does not impact our evidence for a pre-Neolithic occupation at Latnjia.

Following discussion with Prof. Marcello Mannino, who reported the uncertainties error to us, we also explored the effects of classifying three samples from around the Mesolithic-Neolithic transition at Grotta dell’Uzzo as Neolithic rather than Mesolithic (labelled as SicilyAlt in Supplementary Table 1 and Supplementary Fig. 1). These three samples (MAMS-40712, MAMS-48212, KIA-36032) are somewhat ambiguous in terms of their cultural/population affinities. As shown in Supplementary Table 1, the categorisation of these samples as either Neolithic or Mesolithic has little impact on the overall timing of the Mesolithic/Neolithic transition in the region.

Supplementary Table 2 summarises these results to highlight how the different models and interpretations have little overall impact on our interpretations of the chronology of the broader regional shift from the Mesolithic to the Neolithic. The results across all models demonstrate a probable onset of the Neolithic in Sicily between ca. 8 and 7.5 ka, which is the widest interval containing the 95% highest density regions of the posterior densities for Neolithic start boundaries across phase models. The end boundary interval for the Mesolithic has a less tightly constrained chronology of between ca. 8.7 and 7.2 ka.

These corrections are reflected in the updated GitHub repository. A new release was issued and the corrected repository has been archived with Zenodo, and can be found using the same DOI as the previously published version. The original version could be reviewed by looking through the commit history of the repository, but for convenience we also created a ‘legacy’ branch, which can be viewed at https://github.com/wccarleton/mesoneomalta/tree/legacy and we preserved relevant legacy files in correspondingly named folders within the main branch.

We thank the anonymous peer reviewer who assessed these corrections and Prof. Marcello Mannino for discussions.

**Supplementary information** is available in the online version of this amendment.

## Supplementary information

Supplementary Fig. 1 and Supplementary Tables 1 and 2
