# Supplementary material for: Hunter-gatherer sea voyages extended to remotest Mediterranean islands
Source: Nature. 2025 Apr 9;641(8061):137–43. doi: 10.1038/s41586-025-08780-y (PMC12043505; doi:10.1038/s41586-025-08780-y)
Supplement: Supplementary file 1 — This document contains additional details and information including the context of research (1), a description of the deposits (2), details of the archaeobotany (3), a chronology (4), lithic analyses (5), faunal analyses (6), Supplementary Tables 1–16 (7), OxCal scripts (8) and Supplementary References (9). [file 41586_2025_8780_MOESM1_ESM.pdf]

nature portfolio

https://doi.org/10.1038/s41586-025-08780-y

Supplementary information

Hunter-gatherer sea voyages extended to remotest Mediterranean islands

In the format provided by the authors and unedited

Supplementary Information

Hunter-gatherer sea voyages extended to remotest Mediterranean islands

Eleanor M.L. Scerri[1,2,3*] , James Blinkhorn[1,4*], Huw S. Groucutt[2,3,], Mathew Stewart[5], Ian Candy[6], Ethel Allué[7,8], Aitor Burguet-Coca[7,8,9], Andrés Currás[1], W. Christopher Carleton[10], Susanne Lindauer[11], Robert Spengler[12], Kseniia Boxleitner[12], Gillian Asciak[13], Margherita Colucci[1,14], Ritienne Gauci[15], Amy Hatton[10,16,17], Johanna Kutowsky[1], Andreas Maier[3], Mario Mata-González[1,2], Nicolette Mifsud[2], Khady Niang[1,18], Patrick Roberts[10,19], Joshua de Giorgio[20], Rochelle Xerri[2], Nicholas C. Vella[2*].

Affiliations:

[1]Human Palaeosystems Group, Max Planck Institute of Geoanthropology; Jena, Germany.

[2]Department of Classics and Archaeology, University of Malta, Faculty of Arts; Msida, Malta.

[3]Institute of Prehistoric Archaeology, University of Cologne; Cologne , Germany.

[4]Department of Archaeology, Classics, and Egyptology, University of Liverpool, Liverpool, U.K.

[5]Australian Research Centre for Human Evolution, Griffith University; Brisbane, Australia.

[6]Department of Geography, Royal Holloway University of London; Egham, U. K.

[7]Institut Català de Paleoecologia Humana i Evolució Social (IPHES-CERCA); Tarragona, Spain.

[8]Universitat Rovira i Virgili (URV), Departament d'Història i Història de l'Art; Tarragona, Spain.

[9]Leiden University, Faculty of Archaeology, Department of Archaeological Sciences; Leiden, The Netherlands.

[10]Department of Archaeology, Max Planck Institute of Geoanthropology; Jena, Germany.

[11]Curt-Engelhorn-Centre Archaeometry; Mannheim, Germany.

[12]Domestication and Anthropogenic Evolution Research Group, Max Planck Institute of Geoanthropology; Jena, Germany.

[13] Superintendence of Cultural Heritage; Valletta, Malta.

[14]Evolutionary Ecology Group, Department of Zoology, University of Cambridge; Cambridge, U.K.

[15]Department of Geography, University of Malta, Faculty of Arts; Msida, Malta.

[16]The Department Structural Changes of the Technosphere, Max Planck Institute of Geoanthropology; Jena, Germany.
[17]Institute for Archaeological Sciences, University of Tübingen; Tübingen, Germany.
[18]Département d'Histoire, Université Cheikh Anta Diop de DakarUniversity of Cheikh Anta Diop; Dakar, Senegal.
[19]isoTROPIC Research Group, Max Planck Institute of Geoanthropology; Jena, Germany.
[20]Department of Archaeology, University of York; York, U.K.

*Corresponding authors sceri@gea.mpg.de, blinkhorn@gea.mpg.de, nicholas.vella@um.edu.mt

Table of Contents

1. Context of Research 4
  1.1: The context of early seafaring and island colonization in the Mediterranean 4
  1.2: The Maltese record and the Neolithic transition 7
  1.3: The regional character of the Late Palaeolithic and Mesolithic 12
  1.4: The Mesolithic to Neolithic transition in the central Mediterranean 16
  1.5: Future Questions 24
2. Description of the Deposits 26
  2.1: Unit 4 (Matrix rich silty/sand diamicton with rare clasts) 26
  2.2: Unit 3 (Ash/Charcoal rich fine sands and silts): 27
  2.3: Unit 2 (Phorcus rich cut and fill features) 29
  2.4: Unit 1 (clast rich diamictons) 30
  2.5: Site formation processes and human occupation at Latnija 31
3. Archaeobotany 32
  3.1: Pollen and Charcoal Results 32
  3.2: Phytoliths and FTIR 35
  3.3: Plant Macrofossils 38
4. Chronology 39
  4.1: Latnija Age Model 39
  4.2: Phorcus turbinatus chronology 40
5. Lithic Analysis 42
6. Faunal and Isotopic Analyses 45
  6.1: Taxonomy and biogeography 45
  6.2: Species abundance 47
  6.3: Preliminary taphonomic analysis 48
  6.4: Isotopic Analyses 49
7. Supplementary Tables 51
8. OxCal Scripts 73
9. Supplementary Information – References 98

1. Context of Research

1.1: The context of early seafaring and island colonization in the Mediterranean

The Mediterranean is the world's largest inland sea, and it sits at a crucial nexus of continents, around which many societies and states have risen and fallen. Consequently, the origins and character of prehistoric seafaring in the Mediterranean have been much debated (e.g.,[1–5]). The early, pre-Neolithic evidence is sparse, and diverse opinions have been expressed regarding the chronology and implications of early seafaring and island settlement. Clear indications of human presence on islands in the form of unambiguous artefacts or fossils, directly dated, or strongly associated with chronometrically dated materials is still lacking. While it is possible that there were earlier occupations on some of the larger islands, there was clearly a significant increase in maritime engagement in the Mediterranean around the end of the Pleistocene and into the early Holocene (e.g.,[1,2,6,7]). The strongest evidence suggests that these early crossings were preferentially to the largest islands and/or those closest to the mainland. This is thought to be because smaller and more remote islands would have presented challenges for maintaining viable population sizes given the land area required to sustain hunter-gatherer societies[8]. An important aspect of our findings in Malta is that they show not only early seafaring activity, including the longest documented early Mediterranean Sea crossing, but that this was to a very small land mass. This contrasts with the traditional focus on the largest islands of the Mediterranean. To give an example, the Maltese Islands have a combined landmass of about 3% of that of Cyprus, the island which currently has the earliest clear evidence for human arrival.

As will be discussed more below, several islands in the Mediterranean have provided evidence for the arrival of people in the terminal Pleistocene and early Holocene. This has been recorded in the Aegean, and on Cyprus, Sardinia, and Sicily (e.g.,[1,2,9–16]). While in the case of Sicily, the Mesolithic has often been argued to flow from the preceding Late Upper Palaeolithic ('Epigravettian'), in other cases these early Holocene Mesolithic societies represent the first arrival of people on Mediterranean islands. Broodbank[1] links this development to the climatic shock of the Younger Dryas. It has also been argued that the Mesolithic shows indications of increased marine subsistence compared to the preceding Upper Palaeolithic (e.g.,[6,7,10]). The distinction between the Late Upper Palaeolithic and the Mesolithic is rather blurry in the region, and as traditionally divided, the difference is largely

one of time, with the Mesolithic being used to refer to the societies of the warming period of the early Holocene.

There are ambiguous signs of very early occupations of some Mediterranean islands. In certain cases, what are today islands were actually joined to the mainland by land bridges when humans occupied these areas, due to lower sea levels. In other cases, there were very narrow sea crossings. The example of Sicily, which was occupied by around 16 ka, is prominent here (e.g.,[17]). Today the gap between Sicily and Calabria is less than three kilometres, and during the Last Glacial Maximum there was seemingly a land bridge[18]. In other cases, the ambiguities about early occupations revolve around issues, such as distinguishing geofacts from artefacts, obtaining reliable chronometric age estimates for claimed findings, and distinguishing natural from anthropogenic accumulations. The claims of Acheulean and Middle Palaeolithic presence in Crete may suggest very early arrival on some Mediterranean islands[19,20]. However, the findings are restricted to possible lithics from undated contexts. In any case, these findings may date to periods of lower sea levels, when Crete may have been as little as three kilometres from the mainland[21]. Further research is needed to confirm if there really were Acheulean and Middle Palaeolithic hominins on Crete[5].

It is around the time of the transition from the Pleistocene to the Holocene that signs of longer sea crossings become evident. Previous knowledge suggested that there was a clear pattern to the age at which people first arrived on Mediterranean islands, with those that are larger and nearer the mainland being occupied first[5]. The earliest evidence, although much debated, comes from Cyprus[2,12,15,22], which is a very large island, visible from the mainland, and with amenable currents around it for arrival from the mainland. Today Cyprus is less than 70 km from the closest adjacent mainland, and this would have been reduced during periods of low sea level. The combination of enlarged coastal plains in southern Turkey and the emergence of small islands during times of low sea level means that crossings of less than 50 km were necessary to reach Cyprus. In other cases, colonization of islands such as Corsica and Sardinia, even during periods of high sea level similar to the present, involved island hops of no more than 30 km. Low-sea level would have further exaggerated this, especially for islands such as Melos and Crete. These would all have been achievable using dugout canoes, which are the only known form of sea vessel from this time (e.g.,[23,24]). No evidence

for ships with sails is known before their appearance in Egypt and the eastern Mediterranean around 5/5.5 ka (e.g.,[2,25,26]), several thousand years after the Mesolithic occupation of Malta.

The discovery of pre-Neolithic occupation on Malta is, therefore, significant as it shows that people were making much longer trips in simple vessels to small and remote islands earlier than had been thought. This is in contrast to human arrival on large and nearshore islands, which were previously known in terms of Mesolithic settlement. The spread of humans to Malta may have been near the limits of regional Mesolithic adaptation, as is perhaps indicated by a variety of islands which lack evidence for early people. The Balearic Islands are pertinent in these regards. Ibiza is around the same distance from mainland Spain as Malta is from Sicily, and in turn Mallorca is a similar distance (ca. 85 km) further onwards, and no Mesolithic (or even Neolithic) evidence has been found in these islands. While debated, it seems the earliest evidence for people in the Balearics is around 4.5 to 4 ka (see e.g.,[4,27]). The relatively late arrival of humans in the Balearic Islands may reflect the strong currents that run southwards between the Balearic Islands and the mainland, making crossings in simple dugout canoes challenging. Additionally, it may result from the fact that - at least with current sea level - Ibiza is just visible from the mainland, and in turn Mallorca is at the limits of visibility from Ibiza.

Pantelleria is a source of distinctive obsidian that was later widely distributed and provides a clear indication of interactions between the island and adjacent landmasses. Pantelleria is around 70 km from Tunisia and 100 km from Sicily, and current evidence suggests that people first arrived there in the Neolithic (e.g.,[28,29]). There have been some suggestions of Pantellerian obsidian in pre-Neolithic settings in the Maghreb, which would imply early seafaring (e.g.,[30]). The evidence is currently somewhat ambiguous[28,31] (*contra*[32]). The key site is Hergla SHM-1 in Tunisia, where a few pieces of obsidian were reported by Mulazzani and colleagues[30]. However, these are small fragments of obsidian (<20 mm), in settings which may well have some stratigraphic mixing, since a few pieces of pottery seem to be intrusive. Although the Maghrebi evidence *may* indicate early obsidian transport and hence seafaring, it should currently be considered with caution. Future excavations may help to clarify the evidence from Tunisia. Additionally, sea level changes impact the distances between North Africa, Pantelleria, and Sicily; notably, during very low sea level, such as during glacial maxima, a large landmass is exposed extending from southwest Sicily[33]. This reduces the distance from Pantelleria to Sicily to around 25 km. Further west on the exposed land

enlarging Sicily, it would have been possible to island hop to North Africa with sea crossings of no more than 25 km. However, by the period in question, some eight thousand years ago, most of this exposed land had been submerged and Pantelleria was once again a remote island.

The seemingly 'negative' examples of Pantelleria and the Balearics may, therefore, suggest that the settlement of Malta was at the limits of the abilities, or ambitions, of Mesolithic societies in the region. However, aside from the possibility that such evidence may one day be found in these places, one final aspect should be kept in mind. The similarities between early Holocene, pre-Neolithic technologies on either side of the Mediterranean have long been noticed[32]. While wrapped up in different regional nomenclatures (e.g., the Capsian of the Maghreb, the Castelnovian or 'Second Mesolithic' of Italy, and other such terms) and research methods, an argument can be made that similar technological changes occur at similar times in areas like Italy and the Maghreb. As discussed by authors, including[34], this is not simply in the form of the synchronous prominence of trapezes in terms of the retouched toolkit, but also in techniques and methods of blank manufacture, such as pressure flaking. This situation either reflects a fascinating example of convergent technological development – for wider perspectives on which see[35,36] – or hints at trans-Mediterranean crossings. It is by looking at Mediterranean islands that we might distinguish these processes. Our findings from Malta indicate the possibility of these early crossings of the Mediterranean, but, as explored further in this section, the lithic technology at Latnija is of a very simple character, and so does not in that sense tell us about the relationship between Capsian and Castelnovian material culture.

1.2: The Maltese record and the Neolithic transition

The archaeological record of Neolithic to modern Malta has long been celebrated, as reflected in the presence of multiple UNESCO World Heritage sites in the small landmass that makes up the archipelago.

Maltese prehistory and history are characterized by repeated population arrivals, and there is debate about the extent of subsequent population continuity and behavioural change. In the traditional narrative, the first people to arrive in the islands were Neolithic farmers coming from Sicily, around 7.5 to 7 ka. The Early Neolithic is known from sites, such as Għar Dalam and Skorba (e.g.,[37,38]). Dramatic changes occurred during the Late Neolithic, or Temple

Period, when the distinctive megalithic 'temples' appeared and prospered (e.g.,[37–41]). This was followed by the Bronze Age[42] and more recent societies.

Claims for very early, Palaeolithic, occupation of the islands by Neanderthals (e.g.,[43–45]) have generally been rejected on both morphological and chronological grounds (e.g.,[37]). Anati[46] suggested a possibly pre-Neolithic date for cave paintings at Għar Ħasan in southeast Malta. However, our recent re-evaluation of the site has challenged this hypothesis and suggests that at least the vast majority of paintings in the cave are of a very recent date[47].

The dominant view, then, has been that the Maltese Islands were uninhabited until the arrival of Neolithic farmers around 7.5 ka. Evidence for the origin of the Neolithic can be seen in both 'direct' (i.e. archaeological sites) and 'indirect' (i.e. interpretations of pollen data from coastal/near-coastal settings) terms.

Thanks to a suite of new radiocarbon dates produced by the FRAGSUS project, important information has become available. The earliest direct Neolithic evidence is a wheat seed from Santa Verna, directly dated to 6412 ± 44 radiocarbon years ago[48], which calibrates to 7.3/7.4 ka. A further dozen dates over 6000 radiocarbon years old are available from domesticates, giving a relatively continuous sequence of dates for the early Neolithic in the Maltese Islands[48]. This chronology is consistent with indirect evidence for fire regime/ecological changes, which might indicate new forms of landscape use, namely a spike in charcoal recorded in a core from Marsa around 7.5 ka[49]. Based on this evidence, the Neolithic in Malta most likely begins around 7.5 ka/7.4 ka, consistent with the wider regional timing of the transition to the Neolithic.

A claim has been made for an earlier origin of the Neolithic in Malta, around 8 ka. Hunt and colleagues[50] report that the Salina Deep core contains evidence for domesticated plants at around or just under eight thousand years ago according to their age model (and a date of 6067-5821 cal. BC from charcoal from the unit containing the indication of the Neolithic itself). They also highlight coprophilous fungal evidence potentially consistent with the presence of domesticated grazing animals, but which could also reflect wild fauna. However, the sequence contains multiple age inversions and the chronological precision is questionable. The closest radiocarbon dates below the apparent Neolithic onset produced ages in the ca. 9-8.7 ka age range, yet the two next dates *beneath* those are one thousand years *younger*.

Overlying dates, above the inferred start point of the Neolithic are again not in good stratigraphic order, and cluster around 7.5 to 7.3 ka. Given the multiple age inversions in the sequence it is quite possible that the indirect evidence for the arrival of the Neolithic from the Salina Deep core is actually considerably younger than suggested by the authors, but even if the ‘long chronology’ for the Maltese Neolithic is accepted (which would make the Neolithic in Malta older than in Italy, from where it is believed to have spread), this would extend no further than around 8 ka.

To evaluate the notion that the Salina Deep record supports an earlier origin of the Neolithic in the Maltese islands, we conducted new age depth modelling (for replication information see R scripts and data available on Github, https://github.com/wccarleton/mesoneomalta). The suggested first presence of the Neolithic occurs at 2250 cm. In Extended Data Fig. 2, Panel A, we plotted the radiocarbon dates available for the sequence, along with the pollen sequence reported by Farrell and colleagues[51]. We used the BChron R package[52], repeating one of the methods used by Hunt and colleagues[50]. This is a good approach for gradually accumulating sequences, where strict age-depth monotonicity is highly likely and intrusive chronological are more-or-less easy to identify against a background of otherwise *in-situ* material. However, as Panel A in Extended Data Fig. 2 shows, there were many potentially intrusive samples used to date the Salina Deep sediments and, in fact, very few sequences of dates that appear to be in strict stratigraphic order. As a result, the automated outlier detection used in BChron (and typical of other age-depth modelling software) has led to an age-depth model with dubiously high precision.

In essence, the timing in this model reflects the fact that the model prefers the more tightly clustered group of dates around 2400 cm to the more scattered three dates around 2600 cm (Extended Data Fig. 2, Panel A). Likewise, dates between approximately 2000 cm and 1800 cm are not in good stratigraphic order and hint at problems with the record. Given the potential for age-target sampling variability (not accounted for in current age-depth modelling software) and the lack of clear monotonicity, it is unclear which of the samples should be considered the most reliable indicators of the true age-depth relationship, particularly around the depths associated with the onset of the Neolithic. As Hunt et al.[50] (p. 35) themselves stated, dating these sequences is challenging as “alluvial sediments in Malta were originally deposited during relatively high-energy rainstorms, causing much recycling of organic material and thus radiocarbon results that may not necessarily truly reflect the date

they were buried". Previous work on Maltese sediment cores have likewise noted major discontinuities and other stratigraphic complexities (e.g.,[53]). In the part of the core from which the claims for early Neolithic evidence in the Salina Deep record come, Fenech and colleagues[54] (p. 141) noted sedimentary and molluscan evidence for "massive land-derived erosion" and suggestions of "very rapid sedimentation". It therefore appears problematic to assume that radiocarbon dated samples necessarily give precise ages for particular parts of the sedimentary sequence.

Given the lack of reliable stratigraphic positioning, ambiguity regarding which samples could reasonably be considered intrusive or out-of-order, and environmental context that suggests stratigraphic monotonicity to be highly unlikely, a more conservative approach to age-depth modelling is warranted. Keeping this in mind, we used a simple Bayesian regression model to relate depth to age in the Salina Deep core (Extended Data Fig. 2, Panel B). The model, like any age-depth model, predicts ages of undated sediments using the observable relationship between age and depth in the dated samples[55,56]. Since we do observe an overall general age-depth trend in the data, but have to grapple with the many intrusions, we opted for a simple linear model. To account for chronological uncertainty in the radiocarbon dates, we employed a standard measurement error component, one based on the (also standard) calibration equation involving a mixture of Gaussian distributions to account for both isotope measurement errors and calibration curve uncertainties[57]. The model treats the unobserved ages (in years before present, 'ybp') at each depth in the Salina Deep core as latent variables that are Normally distributed given the known depth and measured indirectly with radiocarbon assays (where available). Thus, the true age of the sediment depth, d, for the n-th core sample is given by

$$age_{true,n} \sim N(\beta_0 + \beta_1 d_n, \sigma),$$

where the age is latent and measured with an error distribution (itself a mixture of lab and calibration curve uncertainties) as follows[57],

$$y_{obs,n} \sim Normal(\mu_{cal}(age_{true,n}), \sqrt{\sigma^2{}_{lab} + \sigma^2{}_{curve}(age_{true,n})}).$$

We used uniform priors for $age_{true,n}$, a uniform prior for σ, and normal (highly uninformative) priors for β_0 and $\beta_{\cdot 1}$

The model parameters were estimated using Markov-Chain Monte Carlo (MCMC) methods and the R Nimble package (https://r-nimble.org/). The radiocarbon calibration error model with implemented with a custom Nimble distribution (see https://github.com/wccarleton/mesoneomalta/blob/master/Src/dcal.R). We then inspected trace plots and used Geweke tests to ensure convergence of MCMC posterior chains. One major benefit of this approach is that Nimble can automatically sample from relevant distributions in cases where data are missing, performing Bayesian imputation during the course of the MCMC simulation used to estimate model parameters. This is how we interpolated the age-depth model to derive (latent) ages for a sequence of un-dated depth measurements from the Salina Deep core. The 95% credible intervals of these interpolated estimates along with the 95% credible intervals of the calibrated radiocarbon dates were then used to plot an uncertainty envelope around the mean age-depth model, revealing a conservative but more accurate assessment of the overall age-depth relationship (Extended Data Fig. 2, Panel B).

With this approach, the calibrated radiocarbon dating uncertainties can be directly accounted for and the general age-depth trend modelled without questionable or undue assertions about outliers leading to unwarranted precision. In our analysis, we used the IntCal20 calibration curve[58] to calibrate the dates. Using this approach, which makes a more accurate and robust accounting of the considerable uncertainty involved, the results suggest that the first Neolithic evidence in the Salina deep record has a date with a broad error range of around two thousand years. The date for the relevant sediments could therefore be less than 7 ka, and the record is certainly consistent with the other local evidence for the Neolithic beginning around 7.5 ka and the regional picture of the Neolithic transition discussed more below.

Given the evidence from Latnija, and the lack of robust evidence for a Neolithic presence before around 7.5 ka, we can begin to consider the dynamics of Maltese Mesolithic society. The small size of the islands suggests that long-term continuity of hunter-gatherer populations was unlikely, as they would have been unable to support more than a few dozen people. We are therefore probably looking at a situation of repeated small, and possibly

seasonal, phases of occupation. This appears to be somewhat analogous to the much larger island of Sardinia. Lugliè[59] (p. 289) described the Sardinian Mesolithic as a matter of "irregular almost ephemeral human presence". Not only is this interesting in terms of the Mesolithic to Neolithic transition, but also in terms of the Mesolithic itself. For instance, we think it is unlikely that humans were able to survive on Malta for thousands of years after the land bridge to Sicily disappeared in the Late Pleistocene. The demographic limitations implied by the characteristics of Malta are, therefore, an important part in arguing that the Mesolithic presence implies long-distance seafaring.

The extent to which different 'push' and 'pull' factors related to the journey of Mesolithic human groups to Malta (see e.g.,[60] for somewhat analogous discussions for the subsequent Neolithic), and the character of these groups and their behaviour once they arrived in Malta, remain to be understood in the future. While much debate has focused on the earliest arrival of humans on Mediterranean islands, the exact nature of early human presence on islands (e.g., seasonal occupations, repeated short phases of occupation, etc.) remains to be elucidated. Dawson[4,61], for example, discusses the character of human presence on Mediterranean islands. In the following pages, we discuss regional knowledge on the Mesolithic and the transition to the early Neolithic, to contextualize our findings in Malta.

1.3: The regional character of the Late Palaeolithic and Mesolithic

In the following paragraphs we seek to contextualize the Maltese evidence in terms of its wider regional setting. Due to its proximity, Sicily is central to our discussion, since it has been generally accepted as the route for early humans to move to Malta from. In contrast, the closest points in North Africa, are more than 300% further to Malta, and face challenging currents.

We also consider evidence from mainland Italy and elsewhere in the region in terms of the dynamics of the Late Palaeolithic and Mesolithic and the transition to the Neolithic. Sardinia offers useful analogies, as like Malta it is relatively distant from the mainland, even during low sea level (although much closer than Malta was, except for the occasional episodes when a land bridge putatively connected the islands to Sicily). The arrival of human groups in the Maltese Islands during periods of high sea level contributes to ongoing debates about early island colonization and seafaring abilities.

The Mesolithic and Late Palaeolithic is well known in Sicily (e.g.,[10]), and on other larger islands in the Mediterranean (e.g.,[34]), including Sardinia, as well as mainland areas such as the Italian peninsula. The extent to which smaller and more distant islands were occupied before the Neolithic has been much debated (e.g.,[4,5]). In the cases of the larger islands, the Mesolithic is securely known, but whether there is an earlier presence is unclear. In Sicily, a late Upper Palaeolithic (Epigravettian) is well-known, from several sites. However, Sicily is very close to the Italian mainland and during the Last Glacial Maximum low sea level stand, seems to have joined it to mainland Italy[18].

Places that definitely remained islands throughout the later Middle and Late Pleistocene have less secure evidence for pre-Mesolithic people. In Sardinia, claims for pre-Mesolithic occupations have been made[62,63], but the evidence is limited, and has been questioned by some (e.g.,[64–66]), but accepted by others (e.g.,[2]). Our point here is that findings from places such as Sardinia and eastern Mediterranean islands (e.g.,[22]), contribute to a set of debates on the reality/extent of pre-Neolithic/Mesolithic seafaring in the region. We do not expect to fundamentally resolve that debate here, but simply to highlight the context for our findings in Malta. Whatever one's opinion on particular sites and sequences, it is clear that in many cases the chronometric dating is challenging and it is possible that claimed artifacts are geofacts, among other ambiguities. Across the Mediterranean basin evidence for seafaring associated with the Mesolithic has been growing, while earlier claims remain controversial.

Sardinian Mesolithic sites are known from around 10 ka (e.g.,[9,67]), by which point sea crossings of approximately 30 km between islands would have been involved. While behaviourally complex, with evidence for various trapping, fishing, and hunting strategies, stone tools of the Sardinian Mesolithic are generally simple and have been classified as part of the 'Undifferentiated Epipaleolithic'. Similar points apply to adjacent Corsica[68]. As summarized by Lugliè[59], the Sardinian Mesolithic is faint and 'ephemeral'.

In Sicily, claims for very early human occupations have been made (e.g.,[69]), but are generally seen as problematic[17]. For instance, crude bifacial tools have been found in Neolithic contexts and are not indicative of belonging to the Acheulean as had been suggested (e.g.,[70]). It must be remembered that Sicily in its current form is geologically very young, and consisted of different islands until the eruption of Mount Etna and other processes that occurred in the Middle Pleistocene. The geology that forms modern Sicily and Calabria are the result of rapid

(geologically speaking) eastward movement of various islands. Given that proximity of Sicily to Calabria is, therefore, a recent phenomenon, it might be the case that there was only a single period in which Sicily and mainland Italy connected, the Last Glacial Maximum[18].

A commonly cited (e.g.,[2,70]) claim for an early, pre-Last Glacial Maximum, human presence in Sicily comes from the site of Fontana Nuova[71]. This claim was based on purported similarities of the lithic assemblage from the site with Aurignacian assemblages from mainland Europe. However, subsequent radiocarbon dating of the site showed that it, in fact, dates to around 9000 years ago, in the Mesolithic[72].

The first clear evidence for human occupation on Sicily dates to between 16.5 and 12 ka, in sites which are late Upper Palaeolithic, typically described as Epigravettian[17]. This is known from several sites and therefore appears robust (e.g.,[73–76]).

The distinction between the late Upper Palaeolithic and the Mesolithic is less clear than in areas such as northern Europe. On Sicily, the Mesolithic can be used to refer to early Holocene hunter-gatherer occupations. The most well understood Sicilian Mesolithic sites are in the northwest. Grotta dell'Uzzo is the key Mesolithic to Neolithic site in Sicily[77–80]. As well as its lithics and human burials, Grotta dell'Uzzo preserves extensive faunal assemblages which offer insights into the Mesolithic to Neolithic transition in Sicily. The Late Mesolithic saw a focus on terrestrial animals (deer, etc.), but with significant numbers of birds and in the final stages of the Mesolithic a significant increase in maritime resources, including fish, shellfish, and whales (e.g.,[77]). This is particularly prominent during the so called 'Mesolithic-Neolithic transition' layers, which in the absence of domesticated taxa and demonstrably *in situ* pottery are best classified as Mesolithic. We will return to the site in terms of the subsequent Neolithic transition. Various other Mesolithic sites in Sicily have been published to varying levels of detail (e.g.,[11]).

Other sites illustrate some similar features to the Grotta dell'Uzzo and, therefore, begin to hint at some regional patterning, but these too need to be seen in their context. Grotta d'Oriente in the Egadi Islands shows a significant increase in marine diet from around 10 ka[81]. Other late Mesolithic sites contain frequent marine molluscs (e.g.,[11,82]). Just as with Latnija in Malta, sites such as Grotta d'Oriente and Grotta dell'Uzzo show large numbers of marine molluscs, including *P. turbinatus*. As Broodbank[2] (p. 179) commented, it is "an

exaggeration, but a pardoningly illuminating one, to characterize the Mesolithic as the age of the snail, and of molluscs in general".

Other findings are consistent with an increased engagement with the sea at the Sicilian Late Mesolithic sites (e.g.,[11,82,83]). This arguably provides context for the Maltese record that we are reporting in this paper. The extent to which increased use of coastal and marine resources meant improved abilities, and/or increased desire, to make sea crossings is currently unclear, as much of maritime subsistence reflects easy-to-collect near shore resources and naturally beached species[77]. In the eastern Mediterranean, zooarchaeologists have used prey choice model of optimal foraging theory to associate an increase in marine resource use during the Mesolithic with increasing human foraging pressure, lower residential mobility, and/or shifting shorelines[6,7].

Regarding the lithic technology, Mesolithic assemblages from Sicily have generally been discussed in terminological and conceptual terms in common with elsewhere in Italy (e.g.,[11]). Table S8 attempts to summarize the key features and chronologies for these different facies. It is worth noting the very small number of assemblages assigned to these different Mesolithic facies in Sicily. The Castelnovian assemblages (n=3) include evidence for a late occurrence (less than 9 ka), whereas the other forms date to over 9 ka. Given that only two assemblages are categorized as 'Undifferentiated Epipaleolithic' by Lo Vetro and Martini[11], it is possible that such forms continued into the more recent past. In Sardinia, all Mesolithic occurrences are of the 'Undifferentiated Epipaleolithic' form. This is important to note as, particularly in combination with poor quality raw materials in Malta, we should not be surprised to see simple technologies in the Maltese Mesolithic, whereas a superficial read of the regional literature might suggest that all of the Mesolithic shows complex features such as backed technologies. On current knowledge, the late Mesolithic of Sicily emphasizes the production of trapezes, similarly to the more well studied Castelnovian assemblages from northern Italy[11].

A point worth mentioning is that Fontana Nuova[71,72], discussed above, lacks the backed forms and microliths that are generally abundant in the Epigravettian and Mesolithic of Sicily, and hints at greater levels of technological diversity in Mesolithic Sicily than might initially be apparent. This observation should be kept in mind when considering the technology of the earliest occupants of Malta.

Other aspects of material culture and subsistence are relevant in discussions of the Mesolithic to Neolithic transition in the area. For instance, at Grotta dell'Uzzo there is evidence for the use of heated clay as a fire-hardened flooring material[70], which, while not representing the production of enclosed vessels, does show an experimentation with different materials in late prehistory. Likewise, stable isotope analysis on marine gastropods suggests evidence for the use of Grotta dell'Uzzo year-round, which might indicate increased sedentism[80].

The latest genetic evidence highlights the population dynamism within the period archeologically labelled as Mesolithic. For instance, Yu and colleagues[84] modelled earlier Mesolithic humans from Grotta dell'Uzzo as having affinities to the 'west European hunter-gatherer' group, whereas the Late Mesolithic people have an additional element of 'eastern European hunter-gatherer' ancestry. As well as this population dynamism, stable isotope data from Grotta dell'Uzzo highlights the diversity of diet in the late Mesolithic[84]. We will return to these points in the following section.

1.4: The Mesolithic to Neolithic transition in the central Mediterranean

The westwards spread of the Neolithic through Europe and the Mediterranean has been the topic of considerable research and debate. Many ideas have been expressed on the topic of the transition to the Neolithic representing population movement, cultural exchange, or independent invention (or some blend of these processes). There has also been discussion on whether the spread was along a broad-fronted wave of advance or was more of a patchwork with 'leapfrogging' movements (e.g.,[85–89]). Archaeologists continue to debate the fundamental meaning of the Neolithic and its spread (e.g.,[3,89–98]). Traditionally the onset of the Neolithic in the Mediterranean and Europe was seen as the appearance in the archaeological record of domesticated plants and animals, pottery, and permanent settlements (and also with aspects such as the common use of particular technologies [e.g.,[99]]). While the extent to which these elements and others are required to constitute a full Neolithic 'package' is debated (e.g., see discussion in[96]). For instance, it is now clear that foragers made pottery in some parts of the world. While in parts of Europe it appears that different elements of the Neolithic did indeed take time to come together, in areas such as the Aegean and much of the central Mediterranean, virtually all of the elements occur together at an initial stage (e.g.,[93]). As we will discuss below, claims have been made that Sicily follows a rather different pattern

from this general model of a relatively abrupt Neolithic transition in the Central Mediterranean.

While some have argued for the uptake of the Neolithic by existing Mesolithic populations in Europe (e.g.,[100]), many objections have since been made (e.g.,[90,101]). More recently, the advent of ancient DNA analysis has cast further doubt upon the extensive *in situ* development of agriculture in Europe (e.g.,[102–105]). Genetic evidence has generally been interpreted as suggesting a close correlation between the transition to the Neolithic and the movement of new human groups into and through Europe. In material culture terms, the distribution of impressed pottery from the western Aegean to Iberia marks a very clear spatial patterning, typically correlated with the westward movements of human groups (e.g.,[94]).

Much of the recent debate on the character of the Neolithic spread and most genetic analyses concern Europe north of the Alps (see for instance[97] and references therein). In what follows, the relative paucity of information in many regions must be kept in mind, and with this the chance that future discoveries may lead to significant revisions.

An important point to note is that there are some indications of incongruence between narratives for the transition to the Neolithic at a Mediterranean-wide scale, and at a local scale particularly in Sicily. Most large-scale studies for the spread of the Neolithic agree on the basic pattern and chronology of spread, be it at a Mediterranean/European scale (e.g.,[3,94,106,107]) or an Italian scale[14,108]. Most researchers suggest that the transition to the Neolithic in Sicily was around 7.7 ka, fitting with the spread across the wider region. As discussed below, however, some claims have been made for the Neolithic occurring centuries earlier in Sicily, which obviously would have implications for the Maltese record. We engage in a formal manner with available radiocarbon data below. We will, however, first make some broad points in relation to debates on the chronology of the transition to the Mesolithic in the following paragraphs.

While most broad-scale analyses in recent years have emphasized processes of population spread in the transition to the Neolithic, persistent narratives from the Central Mediterranean have argued for an *in situ* adaptation of aspects of the Neolithic package by indigenous Mesolithic groups. In Sardinia, for instance, Dyson and Rowland[109] argue for a slow, gradual, and piecemeal adoption of aspects of the Neolithic. In their model these changes are driven

by things such as the development of the obsidian exchange network, in which obsidian from Sardinia was exchanged for pots, animals, etc., from Neolithic groups. Arguing against such a view, archaeological evidence in Sardinia suggests a discontinuity in human habitation of the islands for perhaps several centuries, on either side of approximately 8 ka. Genetic evidence likewise suggests that the Neolithic people are very distinct from the late Mesolithic people[110]. Finally, archaeological findings from Sardinia have been interpreted as in fact showing an essentially complete Neolithic package, from its earliest appearance on the island, such as at Filiestru Cave[111]. While pottery was first introduced with the Neolithic, it is also significant that lithic technology and typology also dramatically changed between the Mesolithic and the Neolithic in Sardinia[59]. These findings have led to suggestions that the transition to the Neolithic in Sardinia "has more and more the aspect of a sharp rupture that led to the first permanent occupation of the islands" (ref.[59], p. 285; see also[14]). Webster[112] offers an extensive review of the Sardinian Neolithic.

The key Sicilian site for documenting the Neolithic transition is Grotta dell'Uzzo[77–79]. The stratigraphy of the site is somewhat complex as it was excavated in several small trenches, and it is not always evident how the different sequences correlate with each other. The key stratigraphic sequence discussed in the literature comes from Trench F (e.g.,[77]). The first Neolithic layer is taken to be context 10. Small numbers of domesticated fauna appear in this layer, and pottery is also present. Interestingly, there is a continued increase in marine fauna, which reaches a peak in the Early Neolithic[77]. Over the subsequent millennia there was a decrease in marine and hunted terrestrial fauna, and gradual increase in the proportion of domesticated animal remains. The problem with Grotta dell'Uzzo is that it is located very close to the coast in an area with a steep coastal shelf. In other words, the distance from the sea would not have significantly changed in the recent past, as this is an area where rugged highlands come right down to the coast. This is not a very suitable area for growing many of the classic Neolithic crops, nor rearing cattle. Given the site's role as a key sequence for the Mesolithic to Neolithic transition in the region, these particularities should be kept in mind.

It is unfortunate that the chronology for the Neolithic ditched villages of southeast Sicily (including Stentinello, after which the Stentinello Phase is named) remains essentially unknown. In terms of pottery, it has been argued that sites in the northwest, such as Grotta dell'Uzzo, show an initial Neolithic phase characterized by archaic impressed ware, whereas the slightly younger impressed ware traditionally described as the Stentinello phase could

correlate with the age of the ditched villages. In this view, the expansion of Neolithic populations led to the ditched villages, with the ditches seen as defensive constructions[70], and this may be the framework in which population movement to Malta occurred. In the absence of direct dating this framework must remain rather speculative, and it should be noted that in mainland Italy, ditched villages are present from the earliest Neolithic. For a general review of the Sicilian Neolithic, see[13,113].

In terms of claims for early (i.e. pre-8 ka) Neolithic sites in Sicily and the wider region, a number of points can be made. Large scale studies positing early dates need to be carefully evaluated. For instance, Betti et al.[106] published a paper on the spread of the Neolithic by different routes from Southeast Europe. The essence of their argument is that the more northerly routes, moving through central and eastern Europe, show a considerable reduction in the speed of the spread of the Neolithic, which they attribute to populations moving into areas with different environmental characteristics. In contrast, they argue that the 'Mediterranean route', across the Adriatic and moving west to southern France and Iberia does not show this slowdown. This suggestion of an extremely rapid Neolithic spread around the southern European rim must be carefully evaluated in terms of the underlying data. The claims for Early Neolithic sites in south-central and southwest Europe rely on an extremely small number of data points. Claims for Early Neolithic sites in Iberia have been convincingly critiqued in terms of factors such as the 'old wood problem' (e.g.,[114,115]). Likewise, the very early Neolithic sample on the Atlantic coast in France in Betti et al.[106] can be questioned. For instance, Bailey and Spikins[116] describe La Lede du Gurp as being Mesolithic rather than Neolithic as suggested by Betti et al.[106].

Likewise, one of the Italian 'Neolithic' dates accepted by Betti et al.[106] is an uncalibrated date of 7555 BP, which calibrates to around 8.4 ka (lab code: R-285), described as being from a site called Praia di Mare (Grotta del Santuario della Madonna at Praia a Mare)[117]. The fact that this date is several centuries earlier than other suggestions for the Neolithic area makes sense in the light of renewed research at the site. Tagliacozzo and colleagues[118] opened a new trench adjacent to one in which the original radiocarbon sample mentioned above had come from. They found clear evidence that the Neolithic occupation disturbed the underlying Mesolithic layers, and so even if the date is accurate, it appears to be a disturbed a Mesolithic related sample. This interpretation is supported by the fact that newly dated samples from the

site associated with Mesolithic deposits produced younger ages than the supposedly Neolithic date from the 1960s[118].

Large-scale radiocarbon-based studies are an important way to evaluate the spread of the Neolithic, but they need to be carefully interrogated by future studies and the results continually evaluated. Another example is that of Parkinson et al.[14], who have some samples marked as Neolithic in their database which are very early (e.g., over 8500 radiocarbon years in one case in Italy). Does this suggest the possibility of a much earlier Neolithic than generally accepted? Upon checking the original publication, it is clear that the sample is actually from a pre-Neolithic layer (e.g.,[119]). Clearly, it is hard to carefully check every single one of the thousands of radiocarbon dates in a large database. In the following section we try to clean the database to remove samples from uncertain stratigraphic contexts or those for which insufficient information is available. It is also hard to deal with factors such as the old wood problem. Likewise, we follow Whitehouse[120] and indeed Skeates himself who published the dates[121], in rejecting two very early Neolithic dates from Coppa Nevigata, which are in the Parkinson et al.'s database[14]. One is on a shell, and it is unclear what kind of shell this is, so accurate calibration is not possible. The other was on a sample of grain that was subsequently re-dated using modern radiocarbon methods and shown to be much younger (OxA-1475). Our analyses in the following section have therefore sought to remove problematic dates. We echo here the conclusions of the detailed study by Binder et al.[122], who used a Bayesian chronological model on short-life samples. Their analysis again suggests that old (i.e. over 8 ka) dates are problematic for the spread of the Neolithic in Italy.

In terms of claims from Sicily, one important point to note is that incorrect radiocarbon calibration makes samples appear much older than they are. Use of terrestrial rather than marine calibration makes a sample appear about 500 years older than it actually is (ca. 7.8-7.7 ka). For example this happened for sample OxA2071-31, from the first Neolithic layer (F-10) at Grotta dell'Uzzo[123]. The claims for a very early Neolithic in Sicily are rather incongruous with the wider pattern of Neolithization in the Mediterranean; as Leppard[3] (p. 247) puts it "Sicily either fits well into pattern or deviates substantially from it", the latter possibility being if one accepts the claim for an early Neolithic at Grotta dell'Uzzo.

Other aspects are more interpretive, and in stratigraphically complex and often bioturbated or otherwise taphonomically complex contacts between major depositional phases, some noise

is common. In this regard, the recent paper by Yu and colleagues[84] offers important data, as they directly dated a series of human teeth from Grotta dell'Uzzo, and acquired isotopic and aDNA data from those teeth. This direct dating means stratigraphic complexities are less important than where dating is by correlation with other materials from a stratigraphic context. Yu et al.[84] report a series of eleven Mesolithic individuals, with nine dating to the Late Mesolithic, from 8.7-8.6 ka to the youngest dated Mesolithic individual at 8-7.8 ka. Seven Neolithic individuals were sampled, beginning at 7.5/7.4 ka. The study of Yu and colleagues[84] offers a valuable coupled dataset of genetic, chronological, and isotopic (dietary) reference points. Significantly, two teeth from the supposedly Neolithic layers turn out to have clearly Mesolithic genomes. This is perhaps consistent with some mixing in the sediments around the Mesolithic to Neolithic transition. This should also caution reliance on an *Epinephelus* bone suggesting a Mesolithic age several centuries younger than other samples from the site (KIS-36032). It is, however, also possible that there was a long overlap between Mesolithic and Neolithic groups in Sicily.

In addition to the samples from Grotta dell'Uzzo, with their complex stratigraphic issues and marine reservoir effects, another key early Neolithic date comes from Grotta Kronio, consisting of a date on charcoal of 6750 ± 90 BP (P-2733)[13], which calibrates to ca. 7.6 ka. From both Grotta dell'Uzzo and Grotta Kronio, a series of dates then follow from the 7.6/7.5 ka timeframe and suggest a reliably dated Neolithic phase at this time.

In our opinion the available evidence suggests a relatively late chronology for the Neolithic in Sicily, which we will formally evaluate in the following section. Crucially, more work needs to be conducted on the Neolithic transition in Sicily, and methods such as modern archaeobotany used more extensively[124].

In this regard, it is interesting to note suggestions for lithic continuity from the Mesolithic to Neolithic at sites like Grotta dell'Uzzo[125], in contrast to the situation in Sardinia. Likewise, factors such as the continuity of heavy marine resource use from the Mesolithic to the Neolithic has been argued to indicate a "strong Mesolithic tradition in which various elements of neolithization are grafted, not traumatically and progressively" (ref.[13], p. 50, *our translation*). Arguments for local continuity have been strong in Sicily (e.g., ref.[70], p. 51; ref.[126]). While in our opinion the evidence for demic spread seems reasonable at an overall

regional scale, there does remain uncertainty of some of the specific dynamics of Neolithization in Sicily.

The genetic evidence presented by Yu et al.[84] suggests that the Neolithization process was one primarily driven by the arrival of a new population, with its particular subsistence practices and material culture, but the genetic evidence does suggest some element of admixture/continuity. This implies close population proximity, and interactions between the populations. They demonstrated that the Sicilian Early Neolithic is best modelled with a ca. 7.5% contribution from the local late Mesolithic population. The demonstration of population mixture, albeit a relatively small proportion, opens the door to discussions on population interaction. This scientific discovery offers robust footings to what were previously more theoretical approaches (e.g.,[126]). Interestingly, the chronologically younger (ca. 6.9 ka) Middle Neolithic sample from another site in Sicily reported by Fernandes and colleagues[127] actually had a higher proportion of Mesolithic ancestry (ca. 12%), indicating continued population interaction, as opposed to an abrupt population replacement. Finally, it is worth noting that one of the youngest individuals from Grotta dell'Uzzo showing a Mesolithic genetic ancestry has a stable isotopic signature interpreted by Yu and colleagues[84] as being more typical of a farming diet than a hunter-gatherer one. They interpret this as indicating the adoption of farming by local Late Mesolithic foragers. However, the relevant isotope signal is simply indicating that the individual had little dietary protein from animals. They may simply have been a vegetarian forager, and it should be noted that their nitrogen values fall outside the range associated with the Neolithic group. Even if Yu and colleagues' interpretation[84] of the individual (UZZ88) as being someone biologically from a local Mesolithic background but culturally Neolithic is correct, this is still consistent with the appearance of the Neolithic in the area at less than 8 ka.

Flowing from the above considerations, we conducted standard radiocarbon phase modeling[57] to explore the chronology of the Mesolithic-Neolithic transition in the central Mediterranean, to allow us to contextualize our findings from Latnija. We used the database of radiocarbon dates published by Parkinson and colleagues[14], but removed ambiguous or problematic dates as discussed in the text above. We also checked seemingly anonymous dates, such as one (LTL2154A) which had been incorrectly labelled as Mesolithic. In general, in the region the Neolithic dates fall after those of the Mesolithic. In Central Italy, there is a single purportedly Mesolithic date which is much younger than others for the area, but is a date on a bulk

sediment sample, which might therefore be of dubious accuracy[128]. Northern Italy seems to present a different picture, with Mesolithic groups surviving long after the Neolithic had begun in the central area. We note in passing that further work is desirable on some of the specifics of this. For instance, the youngest Mesolithic dates from northern Italy are from human bones in the southern Alps (ETH-15980, ETH-15981, UtC-202, Utc-7202). Gazzoni and colleagues[129] discuss the possibility that these dates are erroneous, and that compound specific dating might help clarify such samples.

We used OxCal phase modelling to estimate the start and end dates for the Mesolithic and Neolithic phases in and around Malta[57]. We divided the database into regions and labelled the radiocarbon dates as either "Mesolithic" or "Neolithic" on the basis of sample context. Then, we created a separate phase model for each region and used OxCals' MCMC tools to derive estimates for the end of the Mesolithic and start of the Neolithic in each case (Extended Data Fig. 1, Table S9 below). Given the regional scale of the analysis, there was no stratigraphic information to sensibly include within the phase groupings. Consequently, we simply aggregated dates into their appropriate cultural phases without inputting any further structure (prior information) into the models. It is important to note that we also applied published marine calibration corrections where necessary for marine samples (see the OxCal scripts at the end of this SI and in https://github.com/wccarleton/mesoneomalta for specifics). Otherwise, we used the IntCal20 terrestrial calibration curve[58]. OxCal's standard diagnostic indicator (the agreement index) suggested that the data were congruent with the simple phase model and, so, we extracted the posterior densities for the relevant phase boundaries and plotted them (see Extended Data Fig. 1, Table S9 below). As the posterior density plots indicate, there is evidence for a general geographic cline in the spread of the Neolithic in Italy. From southern Italy, the Neolithic spread to Sicily, Sardinia, and Corsica, and then finally to Malta, all in rough agreement with previous research on the geographic pattern of Neolithization in this part of the Mediterranean.

These radiocarbon modelling results present a coherent picture of the Neolithic transition in the central Mediterranean. In Italy and adjacent islands, the latest Mesolithic associated dates fall in the 9-8 ka time frame, although in northern Italy they continue for much longer. The earliest Neolithic dates for the region are in southern Italy, consistent with the notion of a spread across the Adriatic. From there, broadly consistent timing characterizes the spread of the Neolithic to both Sicily and central Italy. This is followed by a subtly later Neolithic onset

in northern Italy, Corsica, and Sardinia. Malta comes later again. This model is consistent with theoretical expectations for the chronology of the Neolithic transition if it is seen as spreading across the Adriatic and then more or less evenly with geographical distance from southern Italy. This model makes claims for a significantly earlier Neolithic in Malta and Sicily unlikely, as discussed above in terms of the problems at an individual site level. According to this regional picture, human presence in Malta in the ca. 8.5-7.5 ka time period correlates with Mesolithic populations.

An interesting point that our chronological model suggests is that the Mesolithic in Sicily survives late, and overlaps with the start of the Neolithic. The youngest direct Mesolithic date from Sicily is 7036 ± 90 radiocarbon years old (MAMS-40712)[84], which comes from a *Homo sapiens* tooth that has a clear Mesolithic genetic signal, and calibrates to 7.7-8 ka. The Sicilian data is biased towards the single site of Grotta dell'Uzzo, from which over half of the Sicilian Mesolithic dates in our database come. Some samples with a heavy marine influence suggest a younger Mesolithic age, perhaps to 7.5 ka or less. However, the combination of stratigraphic mixing and complex marine calibration mean that this young Mesolithic presence should be treated with some caution. Some support for late surviving Mesolithic populations in Sicily comes from the observation that there is a rise in Mesolithic genetic ancestry in the middle[127] compared to early Neolithic samples[84]. In all, the available data suggests that in Sicily the Mesolithic ends and the Neolithic begins between approximately 7.9 and 7.2 ka. Further studies are required to improve the chronological resolution in Sicily.

1.5 Future Questions

With the discovery of a Mesolithic presence in Malta, many new questions emerge. Was this an ephemeral, seasonal presence, featuring brief, intensive occupation of Latnija, and presumably other sites in the islands? Or could these hunter-gatherers have succeeded in exploiting the resources of Malta all year round? It is hoped that ongoing geochronological and isotopic studies may be able to answer this question in future. Building on this, what was the ecological impact of the Mesolithic occupation of the Maltese Islands on the endemic flora and fauna? Although red deer (*Cervus elaphus*) are known from Pleistocene contexts in Malta, until their discovery at Latnija, it was not known with any great security that they may have survived into the Holocene. Although this was likely a survival, the possibility that these animals may also have been translocated must be investigated (e.g.,[130], p. 102). Assuming

they were not, as the most parsimonious explanation, then, were humans responsible for the extinction of at least some of Malta's iconic endemic species and subspecies? If any substantial impact on the ecosystem can be detected, then new questions arise as to whether cascading ecological changes affected the lifeways and choices of the first farmers arriving on the Maltese Islands. These farmers, often thought of as entering a pristine landscape unaffected by human activity, may have encountered an already anthropogenically impacted environment.

Finally, a key question concerns who these hunter-gatherers were. The most obvious and parsimonious explanation is that they originated from Sicily. If this was the case, how did they navigate the journey to Malta, and how did they know it was there? Did they observe it from high ground, or was there some memory of a land to the south? Did earlier Mesolithic hunter-gatherer groups reach Malta too, and perhaps even earlier Upper Palaeolithic populations? Were they able to navigate short hops to Malta as sea levels rose, before distances approximated that of the present day? Although no trace of an Upper Palaeolithic in Malta has yet been reported, it is not beyond the realms of possibility that they were indeed present. With regards to the earlier Mesolithic, it is at least possible that some memory of a 'closer Malta' was retained, before sea levels began to reach present levels, and the Maltese Islands approximate their current configuration.

If the Mesolithic hunter-gatherers on Malta did not originate from Sicily, some may have come from North Africa, although given the distances involved, this seems a far less likely scenario. In any case, the question should be resolvable through ancient DNA, which will be a focus of future studies currently examining the possibility of sedimentary traces. An additional line of research on the relationship between North Africa and maritime activity in the Mediterranean can come from the excavation of further Maghrebi sites containing imported obsidian from Pantelleria[28]. The current ambiguities about this evidence render conclusions premature, yet further excavations can hopefully clarify this topic. The robust demonstration of pre-Neolithic obsidian import would be a clear indication of long-distance seafaring in the region.

2. Description of the Deposits

The full sedimentological sequence exposed in the Latnija excavation is shown in Extended Data Figure 6. This sequence can be divided into 14 Beds on the basis of texture, sorting and color (along with evidence for features such as burning). All 14 Beds can be grouped, on the basis of commonalities in depositional processes, into four Units which record the major large-scale changes in sedimentation patterns that have led to the formation of the sequence. These closely align with the archaeological Phases. The lowest and earliest of these is Unit 4, fine-grained minerogenic sediments with occasional but rare clasts, which consists of Beds 14, 13, 13a and 13b (and the large boulders that these sediments contain, i.e. Bed 15) and correlates with Phase VI. This is overlain by Unit 3, a deposit rich in ash and charcoal which contain abundant *Phorcus* shells and bone fragments. Unit 3 consists of beds 12, 11 and 10 and can be correlated with Phase V. Unit 2 is characterized by a cross-cutting relationship with the underlying sediments and has been interpreted as the tipping of anthropogenic debris (*Phorcus* shells, bone, and charcoal) into artificially cut tips. Unit 2 consists of Beds 9, 8, 7, 6 and 5. Unit 3 deposits become increasingly clast rich upwards and are correlated with Phase III. The final unit is Unit 1 which contains abundant large clasts (cobble and pebble in size) within a fine-grained matrix. Unit 1 varies between being clast and matrix supported and consist of Beds 4, 3a, 3b, 2 and 1 and correlates with Phases IV, II and I. The increasing abundance in clasts through Unit 2 means that, in character, it progressively appears more like Unit 1. It is likely that the processes responsible for the formation of Unit 1 were also operating during the cut and fill phases of Unit 2 with the resulting deposits of the two interdigitating. Indeed it is likely that Phase IV, which is the interface between the ash rich deposits of Unit 3 (Phase V), represents deposits with the character of Unit 1 (Phase II and I) that began to accumulate prior to the cutting of the pits in Unit 2 (Phase III). This is illustrated in the differentiation of Beds 3a and 3b on Figure 2 and Extended Data Figure 6. These four Units will subsequently be described from the base upwards.

2.1 Unit 4 (Matrix rich silty/sand diamicton with rare clasts)

The lowermost Unit is dominated by a matrix of fine sands and silts with frequent granule sized clasts (Extended Data Figs. 6, 7, 8). Pebbles are rare, while cobbles and pebbles are present but very rare. The sediment is pink (5 YR 7/4 Pink) to pale orange (5 YR 7/6 reddish yellow) in colour. The sediments become increasingly yellow/orange towards the contact with Unit 3. Crude stratification is present within this Unit with this expressed through variations in the number of coarse clasts that are present and/or lenses of finer material being

separated by lines of clasts. In thin section the sediments can be seen to consist primarily of limestone (local bedrock) derived material both in terms of the pebbles, granules and sand grains and the fine-grained material that constitutes the matrix (Extended Data Fig. 7A). Quartz grains are also present but these are very fine and are primarily fine sand to silt grade in size and never constitute more than 2% of the slide. In places intraclasts of fine-grained sediments (grey silt and clay matrix with fine sand grains embedded within them) are present indicating that sediments on the cave floor had been eroded and locally remobilised and deposited. Terrestrial mollusc shells (whole and fragmented) and bone fragments are present but rare (Extended Data Fig. 7B), with scarce charcoal fragments only recovered though flotation standing in stark contrast to overlying deposits.

The sedimentary characteristics of the deposits of Unit 4 are typical of sediments accumulating on a cave floor through the weathering and redeposition of the limestone bedrock that makes up the cave walls. The sediment is dominated by limestone derived fine-grained material that is the likely end product of the physical weathering of the bedrock. Evidence for remobilisation of sediments on the cave floor comes from both the crude horizontal stratification and the discrete intraclasts. The former represent the entrainment and sorting of particles of different grain sizes as a result of surface water flows moving across the cave floor, whilst the latter represent the erosion and transport of fragments of consolidated cave floor material during similar surface flow processes. The presence of quartz grains is anomalous considering the dominance of pure limestones in the cave bedrock and it is most likely that these are derived from aeolian input, a common source of sediment supply to landsurfaces across the southern Mediterranean[131]. The dominance of carbonate material in this Unit and the absence of iron or clay hypercoatings suggests that minimal weathering of these sediments occurred post-depositionally. The unaltered nature of the sediments indicates that either; 1) the sediments were deposited relatively rapidly (resulting in burial before weathering could occur) or 2) that the cave floor contained little in the way of vegetation limiting the potential for pedogenic alteration.

2.2 Unit 3 (Ash/Charcoal rich fine sands and silts):

Unit 3 is conspicuous through its colouration and the abundance of macroscopic charcoal fragments. Bed 12 consists of very loose, and relatively pure, ashy material, whilst Bed 10 is more consolidated and contains *Phorcus* shells, charcoal, bones, and limestone clasts. The loose ashy nature of Bed 12 precluded the sampling of this deposit for thin section analysis,

however, micromorphogical samples from Bed 10 indicate some degree of complexity to its formation. Whilst ash is the main component of this Bed limestone clasts (granules and pebbles) are abundant, but these are supported in a matrix that is rich in fine carbonate (sands and silts) as well as ash (Extended Data Fig. 7C). The limestone clasts in this Bed show variable degrees of alteration with some of these being heavily blackened whilst others show no sign of alteration and are relatively unweathered (Extended Data Fig. 7D). Intraclasts are common within Bed 10 but these are heavily reddened and, the absence of high birefringence values in these intraclasts under cross-polarised lights indicate that these are enriched in iron oxides rather than clay minerals (Extended Data Fig. 7E). Large fragments of charcoal are clearly seen in thin section (Extended Data Fig. 7F).

Much of the sedimentary characteristics of this unit provides evidence for intense burning, at a most basic level through the abundance of ash and charcoal[132]. The difference in properties, however, between the most ash rich Beds (12 and 10) indicate that their style of accumulation may be different. As such the relationship between these Beds and the underlying sediments is of interest. In both cases samples for thin section were taken in the uppermost part of Unit 4, directly below the contact with Unit 3, whilst the relationship between Unit 4 and Bed 10 of Unit 3 was recorded in greater detail (Extended Data Fig. 7). The thin section from directly below Unit 3 is characterized by all of the sediment properties that typify Unit 4 (see above) but the sediment is heavily reddened throughout (Extended Data Fig. 7G). Again, this reddening is not a function of clay translocation as the matrix has low birefringance values under cross-polarised light, and is, consequently, interpreted as enrichment of the sediment with iron oxide. Furthermore, this iron oxide is evenly dispersed throughout the sediment and is not arranged in a series of hyper- or hypocoatings. A complex relationship is observable below Bed 10 at the interface with Unit 4 (Extended Data Fig. 6). While at the very southern end of the exposure a zone of reddened sediment (5YR 5/6 yellowish red) characterizes the upper 10 cm of Unit 4 (labelled “lens a” on Extended Data Fig. 6), this zone is cut into in the central section of the trench by a 5 cm thick laterally discontinuous zone of brown (7.5 YR 6/4 light brown) loose sands/silts (labelled “lens b” on Extended Data Fig. 6) within which fine, dipping lenses of ash (7.5 YR 6/2 pinkish grey) are found. In thin section this lens of brown loose sands/silts has a pelleted structure without the presence of an intact, coherent matrix (Extended Data Fig. 7H). The individual pellets consist of limestone clasts, fragments of consolidated and reworked clay/silts (e.g. peds), charcoal and ash.

The variable sedimentary characteristics of Beds 12 and 10 combined with the complex relationship between these Beds and the underlying sediments of Unit 4 indicate that the ash rich deposits of Unit 3 represent a mixture of both *in situ* and reworked evidence for burning. Bed 12 is interpreted as an *in situ* burning horizon on the basis of: 1) the relatively pure, loose nature the ash and 2) the enrichment in iron oxides within the sediments directly below Bed 12 which is interpreted as being a result of thermal alteration[133–136]. The diffuse enrichment of iron oxides throughout this sediment supports the idea of thermal alteration as enrichment through weathering or pedogenesis would typically concentrate iron products around voids or associate them with evidence for clay translocation. In contrast, the sediment architecture of the contact between Bed 10 and Unit 4 suggests a phase of incutting and erosion within which burning products have been redeposited. This is suggested through two main lines of evidence. Firstly, that the sediments of Bed 10 contain both heavily burnt limestone fragments and limestone fragments with no evidence for weathering. This is consistent with the idea that a mixture of burnt and unaltered material had been mixed together and redeposited. Secondly, that the contact between Bed 10 and Unit 4 appears to be characterized by an erosional cut infilled with loose material, the brown pelleted sediments of lens b. The loose and pelleted nature of lens b supports the idea that it is disturbed sediment that has accumulated at the base of a cut feature, whilst the dipping lenses of ash rich material within this lens indicates that this infill accumulated in an episodic manner with thin in washing or collapse features of more pure ash separating phases of deposition of the looser brown material. The complex relationship between Bed 10 and the underlying succession is seen at the most southerly end of the excavation where the loose brown sediment fill is absent and the ash rich deposits of Bed 10 are underlain by sediments of Unit 4 which have a strongly reddened colour. This reddening is more characteristic of a thermal alteration horizon and may indicate that locally, within the sequence, parts of Bed 10 represent *in situ* burning.

2.3 Unit 2 (Phorcus rich cut and fill features)

The uppermost surface of Unit 3 is cut into by a series of south to north dipping lenses of sediment that are rich in *Phorcus* shells and bone. These sediments were too loose to sample for micromorphology and consequently they are described with respect to field observations. Two trends in sedimentary characteristics occur upwards through this Unit. Firstly, in the lower part of this Unit the sediment matrix is rich in sand sized shell debris but this decreases upwards and is replaced by more minerogenic material (sands and silts, 7.5 YR 6/3 light

brown). Secondly, the clast content increases upwards. In Beds 8 and 9 clasts are rare and are, at the most, large pebble in size, however, in Bed 6 clasts are abundant, and in places the sediments are clast supported, and up to cobble in size. Whilst *Phorcus* shells are abundant in this Unit they are frequently discretely distributed through this Unit and only at the contact between Beds 6 and 7 is clearly defined layer that is rich in *Phorcus* shells present. Even at this locality the layer is no more than a single shell thick and no clear stratigraphic arrangement of *Phorcus* layers is present.

That the sediments of Unit 2 represent anthropogenic cut and fill features is indicated by: 1) the well-defined but localized erosional contact between the different Beds of this Unit and the sediments that underly them, and 2) the abundance of *Phorcus* shells within them. These deposits do not, however, suggest that each phase of cutting was characterized by long term usage for the discarding of waste products (see[137] for contrast). As most *Phorcus* occur in isolation it suggests that either material was occasionally and sporadically discarded into these cuts[138] or that material had collapsed into the cut features mixing both natural and anthropogenically collected material in the resulting fill[139]. Even where *Phorcus* shells are concentrated they represent a single layer of shells and do not, therefore, indicate a particularly prolonged or intense phase of usage.

2.4 Unit 1 (clast rich diamictons)

Unit 1 is characterised by the greatest abundance of large clasts (cobbles and boulders) in the entire sequence. In places these clasts are matrix supported (Bed 4) elsewhere they are clast supported (Bed 3). The matrix is fine with both sand and silt present but also with a noticeable clay component. The matrix is reddened (7.5 YR 6/6 reddish yellow) and has a stronger hue than most of the underlying succession. The clasts are all comprised of the bedrock limestone and whilst some facets of the clast are sub-angular many of the clast surfaces are sub-rounded.

Unit 1 is characterised by collapse of the cave wall but the sub-rounded nature of many of the clasts indicate that this wall collapse represents the failure of surfaces that had prolonged exposure to chemical weathering rather than the brecciation of entirely fresh limestone surfaces. The fact that Unit 1 is so coarse, relative to the underlying sediments indicates that a change in weathering occurred during the accumulation of the uppermost deposits. The more consistently orange matrix of Unit 1, compared with the pinkish grey sediments of Unit 4, for example, also indicates that a different style of sediment was being incorporated into the cave

deposits at this time. Furthermore, the abundance of fine material in this Unit suggests that more of this material was being supplied into the cave environment at this point. The similarity in colour between the matrix of this Unit and the residual soil material, typical of pedogenesis in a Mediterranean climate (e.g.,[140]) that is found on the landscape above the cave is used to suggest that during the accumulation of Unit 1 more pedogenically derived material was entering the cave. If this is the case then the abundance of large clasts could reflect the fact that greater pedogenesis associated with heightened vegetation not only delivered more reddened fine-grained material to the cave but also increased the physical biological weathering of the limestone through root growth, which would brecciate the bedrock more efficiently. This combination of processes could explain both the colour change and the sedimentological change seen in this Unit. It is not possible to discount the possibility that the intensive burning that is represented by the ash rich deposits of Unit 3 could have weakened the limestone and accelerated the collapse of the cave wall directly above this activity. It is also, however, possible that weakening by burning made the limestone in this vicinity weaker and, therefore, more susceptible to later biological weathering. Finally, it is important to note that the alternation, and interdigitation, between clast rich and matrix rich Beds indicates that multiple phases of cave wall failure occurred over time and that these phases were likely separated by episodes of cave floor and wall stability. It is during these phases that human occupation, and further potential cut features occurred.

2.5 Site formation processes and human occupation at Latnija

The Latnija sequence can be explained by the interaction between the accumulation of sediment through natural processes (weathering of the limestone bedrock, aeolian inputs, inwashing of reworked soil material, etc.) and anthropogenic use and modification of the cave environment. The lowermost Unit (Unit 4) contains minimal evidence for human activity and is, for the most part, dominated by the accumulation of fine-grained limestone derived sediments and the input of aeolian derived quartz. Extensive human occupation of the cave is indicated by the burning deposits in Unit 3 (primarily Beds 10 and 12) but the stratigraphic relationship between these Beds indicates that they represent a mixture of *in situ* burning and remobilised burning products. Human activity persists during Unit 2 and 1 but primarily through cut and fill activity. The later Beds of Unit 2 and the majority of Unit 1 indicate an increase in the rate of cave wall collapse which could reflect the impact of intensive burning or increased biological weathering of the limestone or the interaction of

both processes.

3. Archaeobotany

Archaeobotany consisted of looking at pollen (Extended Data Fig. 4A), phytoliths (Extended Data Fig. 4B, Extended Data Fig. 9), charcoal (Extended Data Fig. 4C) and seeds (Table S1).

3.1 Pollen and Charcoal Results

Pollen preservation was generally low and a variable degree of corrosion was observed in both samples. Palynomorphs known to be resistant to decay were frequently found. These features, which are expected in archaeological deposits consisting of alkaline soils, suggest that post-depositional preservation bias may have occurred. Subsequent caution inferring the composition of the vegetation producing the pollen record must be taken into account, with results shown in Extended Data Fig. 4A. Sample (034) yielded 10171 pollen/g and sample (048) yielded 10480 pollen/g concentration values, which indicates that, despite poor preservation, the pollen assemblage can be interpreted in terms of flora presence around the site[141].

Both samples are characterized by the dominance of *Erica* pollen with values around 35-40% representation. *Euphorbia* pollen is widely recorded in both samples with values of 20% in (048) and 10% in (034). Cichorioideae is attested with 20% representation in (034) and 9% in (048), whereas Asteroideae is similarly recorded at both samples with values of 5%. Brassicaceae pollen is particularly recorded in (048), reaching 15%, despite being only slightly attested to 3% representation in (034). *Pistacia* undiff. is recorded below 5% in (034), yet no remains were found in (048). Poaceae has values of between 9-5% representation at both samples, similarly to other herbaceous pollen taxa such as Lamiaceae, *Rumex*, Scrophulariaceae and Apiaceae. Around 25% of palynomorphs found were too poorly preserved to be taxonomically identified in both samples. The minor presence of *Pteridium*, *Glomus* and *Chaetomium* spores were furthermore observed.

The charcoal results are summarized in Table S10, with sample ESEM images shown in Extended Data Fig. 4C. Most of the charcoal fragments correspond to *Pistacia lentiscus*, *Pistacia* sp. or cf. *Pistacia*. Other present taxa are *Juniperus*/*Tetracrinis*, *Erica*, Cistaceae, cf. *Rhamnus,* and cf. Fabaceae. The observation of one fragment of *Juniperus*/*Tetraclinis* wood

anatomy with ESEM microscopy suggests that this likely corresponds to *Juniperus*. However, the presence of *Tetraclinis* cannot be discarded[141,142]. The assemblage is also characterized by an important number of undeterminable fragments related to wood anatomy alterations and/or size of the fragments.

The Latnija charcoal record shows similar taxa representation along the sequence in which *Pistacia* is recurrent and abundant. These results are in accordance with other environmental records from the region for the mid-Holocene (from ca. 7 ka)[142,143]. These data allow us to describe shrubby vegetation adapted to the island's open landscape environment. In the Mediterranean there are three species of *Pistacia*: *P. lentiscus*, *P. terebinthus,* and *P. atlantica*. *Pistacia lentiscus* grows in sclerophyllous shrubland formations together with other woody shrubs and is usually associated with *Olea europaea*. *Pistacia atlantica* is distributed in north Africa and is related to open sclerophyllous shrubland[143,144]. Both species could be suited to the Maltese islands[143]. The presence of different *Pistacia* species, such as *P. atlantica,* also indicate slight environmental differences related to humidity/aridity. In the Latnija assemblage the presence of *P. lentiscus* seems clear according to the observed characters of most of the remains, however we cannot rule out the presence of *P. atlantica*.

The potential source area for the pollen assemblage identified in the samples may have been influenced by the morphology of the site. Conditions for deposit formation in rock shelters or similar spots mainly result from weathering of the bedrock and, particularly in this case, from soil erosion from above the doline. Pollen can be incorporated into archaeological sediments via wind, insects, bats, birds, mammals and even humans[145]. Pollen can also enter from upper surface wash, which may include both fresh and reworked pollen derived from previous accumulations, enabling the formation of secondary depositional contexts[146,147]. Since Latnija is an open doline, we cannot discard vegetation inside growing locally that may have introduced pollen into the sediment. On the other hand, evidence of cave wall collapse at the top of Phase IV suggests that the openness of the doline could have been less wide in the past than it is in the present-day, constraining inner flora development. The vertical aperture at Latnija likely made the doline into a sediment trap, and therefore vegetation growing outside could have contributed to the accumulation of pollen inside the doline, with likely more relevance for the potential flora growing near the entrance. The round isodiametric morphology of the chamber[148] suggests little diagenetic factors other than those typically affecting pollen preservation in soil contexts, such as bioturbation, high pH, oxidation,

contamination by water percolation, or selective preservation[149]. Assessing selective preservation is particularly difficult, as in the Mediterranean region high values of decay-resistant pollen grains may not correspond exclusively to taphonomic bias, but to the spread of open habitat vegetation communities[150].

It is worthwhile considering the high presence of entomophilous pollen types occurring on the preserved assemblage at Latnija, with only Poaceae, Chenopodiaceae, *Pistacia* and *Rumex* among the anemophilous spectrum. This feature could be explained as a result of uneven pollen dispersal within the site[151]. Even if this would involve partial under-representation of some of this flora in the assemblage, the overall ecological significance of the vegetation composition remains unchanged.

Pollen content from contexts (034) and (048) indicates the predominance of an open vegetation cover during Phase V, with the dominance of Ericaceae heath, most likely low shrub formation of *Erica multiflora* and *Euphorbia melitensis*. These communities are well adapted to shallow soils and rocky environments such as those characterizing the local environment of the present-day Latnija area. Even though *P. atlantica* should not be disregarded, *Pistacia* undiff. pollen likely reflects patches of *P. lentiscus* shrubs. The occurrence of Poaceae, Apiaceae, Chenopodiaceae and Asteraceae pollen attests the spread of heliophilous herbaceous vegetation communities, strongly suggesting the existence of open landscape with presence of mixed wooded vegetation in this part of the island.

The vegetation as indicated by the palynomorphs preserved in the samples is in line with the outcome of previous research in Malta for the early to mid-Holocene, despite the paucity of data and preservation bias in the palaeoenvironmental records for this chronological period. Most of the Maltese pollen diagrams supply information from around 7.4 to 7 ka[53,142,152], with the exception of Salina Deep, Salina 4 and Xemxija 1, which go back until 8.5 and ~9 ka[51], although as discussed in section 1 of the supplementary text, precise dating of these cores is currently challenging. In these later sequences, evidence of *Er. multiflora*, *Pistacia lentiscus* and *Eu. melitensis* were identified prior to approximately 8.5 ka, indicating the spread of an open landscape with minor tree and shrub development. This vegetation canopy is similar to that from other sites in the southern central Mediterranean during the Early Holocene, as shown in coastal Sicilian pollen records of Biviere di Gela[153,154] and Gorgo Basso[153,155]. Here, the development of heath and lentisk is attested at this time and, as in Malta[142], is interpreted

as an indicator of higher moisture availability conditions succeeding a drier period at the very earliest stages of the Holocene[156]. The pollen and charcoal from Latnija confirm the abundance of these taxa, which probably characterized the local flora even earlier in the Holocene.

Juniperus and/or *Tetraclinis* is also recurrent in the Mediterranean islands, with *Tetraclinis* restricted to the southern areas of Iberia, the Maltese islands and North Africa[143]. *Juniperus*/*Tetraclinis* has been identified as dominant taxa or dominant together with *Pistacia* in charcoal assemblages dated to the Late Glacial in North African contexts related to arid and heliophilous formations under cool environmental conditions[157,158]. At Latnija, its presence is slightly more abundant in Phase V (Table S10).

Wood was probably the main fuel, and the availability of woody vegetation made the island suitable for the maintenance of hunter-gatherer communities. *Pistacia* spp. provides a good fuel and *P. lentiscus* has been vastly used as firewood in the Eastern Mediterranean coastal areas, North Africa, southern Iberia, Italy, and Mediterranean island contexts usually together with other shrubs in the forest composition[157–160]. With a paucity of other trees, such as *O. europaea* or pines, *Pistacia* and *Juniperus*/*Tetraclinis* were the most available trees for fuel.

3.2 Phytoliths and FTIR

The pyroarchaeological record is based on the presence of elements, such as charcoal, ashes, thermally altered sediments, and burned materials that can be easily affected by taphonomic and diagenetic processes[132]. At Latnija, these aspects are described in the sediments and micromorphology study presented in Section 2.

A hearth, or combustion structure, is one of the clearest forms of anthropogenic evidence that can be identified in the archaeological record, and is the result of, at least, a single short event resulting from an occupation[161], or it can represent a long-term accumulation, resulting from recurrent activities in a particular space[162,163]. From an archaeological point of view, combustion structures are also relevant in the organization of space and can be useful in the identification of occupation palaeosurfaces (e.g.,[164–167]). The relevance of fire has been described as a factor in human evolution and is linked to multiple aspects of past life systems such as diet and cooking[168–170], the transformation and gathering of materials[171–173], the maintenance of body temperature and the occupation of cold spaces[174], the modification of

the rhythms between day and night, the occupation of the interior of caves[175,176], and as a catalysing agent of social relations[177].

The analysis of the pyroarchaeological record has been approached mainly with microarchaeological techniques (e.g.,[132,177] and references therein), and the main focus has been on Neanderthals[178], while modern humans have received less attention[179]. Although soil microarchaeology has been the most widely applied (ref.[132], and references therein), other disciplines have also been useful for understanding the pyroarchaeological record. These include mineralogical techniques using Fourier Transform Infrared Spectroscopy (FTIR)[180–182], archaeobotanical disciplines, including the study of phytoliths, charcoals and microcharcoals[183–186], biochemical approaches, with the study of lipids or polycyclic aromatic hydrocarbons (PAHs)[187,188], and geophysical techniques, with archaeomagnetism as the most relevant technique[189,190]. The combination of the study of phytoliths and FTIR has been widely used in combination in order to understand the pyroarchaeological record. This approach allows for the identification and preservation of combustion structures, the recovery of palaeoenvironmental data, and ultimately, the reconstruction of past human behaviour in relation to the use and management of fire (e.g.,[191–198]; among others).

In most cases, the Latnija phytolith assemblages fail to reach the minimum number of phytoliths required for confident taxonomic and anatomical interpretation (Table S11). It is, therefore, necessary to interpret the data with caution and consider them as indicative of presence. In general, Poaceae morphologies dominate the assemblages, with a significant presence of morphologies attributed to dicotyledonous plants (Extended Data Fig. 9, Panel A). Anatomically, phytolith types that come from leaves and, to a lesser extent, inflorescence phytoliths morphologies were identified (Extended Data Fig. 9, Panel A). Among the Grass Silica Short Cell Phytoliths (GSSCP), the range of core-Poaceae are represented, including a greater abundance of C3 grasses (e.g., the Pooid subfamily) and lower abundance of C4 grasses (e.g., the Panicoid subfamily) (Table S11 and Extended Data Fig. 9, Panel A).

The number of phytoliths identified shows different dynamics among the layers comprising the hearth. The samples from the combustion residue (CR) show the highest abundance, while the samples from control (C) and thermal impact (TI) show low numbers of phytoliths (Extended Data Fig. 9, Panel B). The high values in the CR could be related to anthropogenic inputs associated with fire management and use. To make comparisons between samples, the

quantitative results have been expressed in absolute values, as only two samples reached 200 phytoliths, the minimum number of phytoliths to express the values in grams per AIF (Acid Insoluble Fraction).

The most common morphologies in the samples analysed are Elongate entire related to grass leaves, Elongate dentate related to grass inflorescence, and GSSCP Rondel, related to C3 Pooid grasses, among others (Extended Data Fig. 9, Panel A). However, the most abundant phytolith morphotype is Spheroid echinate (ICPN 2.0)[199], also called Globular echinate[200,201] (Extended Data Fig. 9, Panel B). Spheroid echinate is a diagnostic type for the palms (Arecaceae)[202–204] and, based on descriptions by García-Granero et al.[200], Spheroid echinate morphologies found in the samples probably belong to *Chamaerops humilis* (Mediterranean fan palm). The distribution of this taxon spans the Mediterranean Basin, with Italy and Malta as the westernmost boundaries[205]. Mediterranean fan palm, also called European fan palm or Mediterranean Dwarf palm, is the only palm described as a native of the Maltese Islands[205,206]. Unfortunately, the wild population became extinct during the 20th century, and today new varieties have been introduced[206,207].

In general terms, the FTIR results show a mineralogical composition consisting mainly of calcite, followed by clay and, to a lesser extent, quartz (Table S12). The control samples have a similar composition (calcite, clay and quartz) and, overall, the ratio between calcite and clay is similar (Extended Data Fig. 9, Panel C). In all cases, the calcite origin is geogenic and the clays are not altered thermally. The thermal impact samples from the hearth are mainly composed of geogenic calcite and unburnt clay. However, according to published references[177,180], some aspects of the spectra show evidence of thermal alteration of the clays in the form of attenuation of peak 5 (517 cm^{-1}), especially when this peak is compared with the spectra of the control samples[180]. It is possible that the temperature was not high enough (>400ºC) to record the different peaks associated with the thermal alteration of the clay. Three samples from the thermal impact show pyrogenic calcite, possibly because they were collected from the contact areas between the thermal impact and combustion residue or could be indicative of postdepositional processes. The samples collected at the top of the hearth, described as combustion residues, are mostly composed of pyrogenic calcite (ash), and some of them contain thermally altered clay (Extended Data Fig. 9, Panel C, and Table S12).

The microarchaeological results (phytoliths and FTIR) confirm the macroscopic observations made during the fieldwork. It has been possible to identify and characterize the layers that make up the combustion structures, with the presence of ash and a greater number of phytoliths associated with the ash. The combustion residue identified by FTIR and phytoliths are likely the result of more than one combustion cycle, given the thickness of the ash lens.

In general, the combustion structure is well-preserved. Ash is one of the elements most easily altered by post-depositional agents, such as trampling or exposure to the atmosphere. Rapid burial, and/or the location of the hearth close to the wall of the shelter or the recurrence of superimposed combustion episodes may have contributed to its preservation.

The phytoliths are not rich enough to discuss the functionality of the hearth, and the resolution of the sampling does not allow for the identification of the minimum number of episodes that formed it. However, the morphotypes identified, especially the Spheroid echinate associated with the taxon *Chamaerops humilis* (Mediterranean fan palm), provide insights into diverse sources of fuel, and possibly a broader use of palms at the site.

3.3: Plant Macrofossils

A total of 222 seeds or large seed fragments were recovered from the flotation samples from the two seasons; additionally, 4,926 land snail shells or shell fragments, 1,978 bone fragments, and 2,025 carbonized wood fragments (>2 mm) were recovered (Table S1). The Mesolithic assemblage consists of grasses (Poaceae, n=122), small-seeded wild pulses (Fabaceae, n=40), and a few small wild herbaceous plants (Amaranthaceae, Caryophyllaceae, Euphorbiaceae, and Malvaceae; n=41). Other fragments of unidentifiable plant remains (n=19) are, likewise, reported. Collectively, these seeds support the other palaeoecological lines of data presented in this paper, showing that the vegetation around Latnija during the Mesolithic included grassy meadows and shrubs.

Additionally, a seed of a Fabaceae type that resembles a wild vetch (n=26) was recovered from the Latnija 2021 and 2022 assemblages. The specimen shows the characteristic long hilum of *Vicia* species; although most of the specimens are incomplete or poorly preserved. A list of modern wild Fabaceae on Malta consists of 106 species that are adapted to the local environment[208], and further work is needed to narrow down the possible taxon for the specimens. Including the *Vicia* type, we recovered 40 seed remains of wild Fabaceae. Their

carbonized state does link them to humans, whether as unintentional introductions or burned in the sediments below a fire. Small unidentified vetches have been retrieved from Mesolithic and Neolithic layers of several other sites on islands in the western Mediterranean[209,210]. Zvelibil[211] claims that grasses, pulses, including small-seeded herbaceous types, such as Chenopodioideae, found in Mesolithic layers in Greece, Spain, Italy, and France, were actively exploited by people. Given the limited data, we cannot confidently determine whether the wild herbaceous plants served as a complement to marine and terrestrial resources such as red deer for Mesolithic hunter-gatherers on Malta, but their presence and carbonized state does suggest that further study is merited. These charred plant remains from the Latnija site add to the limited evidence for the Mesolithic occupation of the Western Mediterranean islands.

4. Chronology

4.1 Latnija Age Model

Based on the depositional contexts identified by the excavators (SI 2), we created a Bayesian phase model using OxCal[57] (v.4.4, https://intchron.org) for the 33 radiocarbon dates derived from charcoal and bone from the Mesolithic horizon (Phases III-V) and deposits that directly underlie them (Phase VI) (Table S2). A range of palaeobotanical analyses already confirmed that, where identifiable, the charcoal fragments were from *Pistacia* (likely *lentiscus*), and *Juniperus,* fast-growing shrubs that minimize concerns about the effect of old wood (Table S13, see also section 3 above). This is further supported by discrete concentrations of phytoliths within hearth features, suggesting active selection for fuel. The clustering of the majority of the radiocarbon dates indicate good stratigraphic integrity, a fact that is also independently corroborated by dated tip lines of *P. turbinatus* (Fig 2; see section 2).

Only one date was recovered from bone. Attempts to date bones from the initial 2019 test trench not reported here were all unsuccessful, likely due to the hot and humid climate of Malta. It was therefore anticipated that any further attempts would also result in failure. Nevertheless, attempts to date the collagen from a further six bone samples were made. Of these, only one succeeded (Table S2). Given the low probability of extracting ages from the bone, and the fact that the process is destructive, no further attempts to date bone were made. As discussed in section 2 above, individual contexts (stratigraphic units) can be grouped into phases with distinct formation processes. To construct a chronological model, the

archaeological Phases were ordered within a sequence, beginning with the lowest/oldest unit, Phase VIA/B, and ending with the most recent considered in this paper, Phase IIIB. We used "Sigma" boundaries between the phases in the sequence, which effectively allows for the start and end of each abutting phase to overlap. This modelling decision was made to accommodate the potential of gradual or disturbed boundaries between depositional phases to impact dating sample recovery, especially at the boundary of pre-Mesolithic and Mesolithic horizon deposits.

The target of our modelling efforts was primarily the phase boundaries rather than the dates themselves, and these results are presented in Table S2, Table S3, Table S6, and Fig. 2. The IntCal20[58] calibration curve was used to calibrate the radiocarbon dates during the modelling process. The standard metrics used in OxCal to judge the fit of the available dates to the model structure—the agreement indices—indicated an agreement of 75-80% (A_{model}=79, $A_{overall}$=76). For reference, Bronk Ramsey[57] suggests fit indices of at least 60% should be considered the minimum benchmark for accepting a model. The OxCal script needed to reproduce the phase modelling results is available in a Github (https://github.com/wccarleton/mesoneomalta) repository archived with Zenodo (https://doi.org/10.5281/zenodo.14192393.).

The end of the Mesolithic is difficult to constrain owing to the fact that Phase II deposits at Latnija are complex and contain a mixture of materials of different ages. Currently, it seems likely that the Mesolithic ended when the Neolithic took root, similar to events on Sicily. However, further studies, and ideally, further Mesolithic sites on Malta will better resolve this question.

4.2 __Phorcus turbinatus__ chronology

One of the significant features of the Mesolithic at Latnija is the abundance of marine gastropods (particularly *P. turbinatus* and, to a much lesser extent, *Patella* sp.). These were imported by humans to the site. In some cases, such as in Phase III, large quantities of marine gastropods were deposited as discrete tips, providing important evidence for occupation activity. This is especially the case since the tips form clear lines in section, providing important stratigraphic controls (Fig. 2). By dating shells from these Phases, we can evaluate their stratigraphic integrity, for added security.

A further motivation for dating these *P. turbinatus* shells is the fact that the Phase III deposits, containing rich archaeological material, represent a pit fill. In this situation, we wanted to distinguish between models for the accumulation of these deposits, testing, for example, whether dated charcoal samples could derive from redeposited sediments disturbed when the pit was dug out. Some contexts within Phase III, such as contexts (019) and (029) contain extremely high numbers of *P. turbinatus* shells, in discrete tip lines, marking individual dumping episodes (Fig. 2). Even when more isolated occurrences are present, each shell can be individually dated to show evidence for the chronology of human activity. We therefore conducted a program of radiocarbon dating of *P. turbinatus* shells (n=49) from the Mesolithic Horizon (phases III to V).

The challenge with marine samples is that a local marine reservoir effect (MRE) needs to be determined that is species-specific. This endeavour is problematized by the fact that MRE is temporally variable, as it not only reflects the animals' diet, but also the prevailing marine conditions, including sea-level and freshwater input. Latnija requires the determination of the MRE on *P. turbinatus* shells for different Phases, and, therefore, for several chronological periods. A major chronological difference between periods would point to a significantly changing marine environment over time. Minor fluctuations might point to smaller scale changes. However, these may still have effects on human occupation.

The marine reservoir effect calculations are described in the Methods, and calibrated dates for the Phase III-V *P. turbinatus* shells are shown in (Table S4). Our results show changing Marine Reservoir Effect (MRE) through time, from -57.3 ± 128.6 years for Phase V deposits, to 9.5 ± 111.5 years for IV, and 64.2 ± 64.8 years for Phase III (Table S5). This change through time, reflecting changing sea dynamics, is consistent with recent marine reservoir calculations for Malta and nearby Sicily. Marine shells from Malta collected in 1914-19 have average ΔR values of 51 ± 93 years[58], while a shell collected in Sicily produced a value of 71 ± 50 years[212]. The fact that the two recent samples from Malta used to create this average value were quite different from each other (-42 ± 24 years and 144 ± 24 years) highlights the variability of the reservoir effect. We interpret this as reflecting different levels of freshwater input where the shells were collected. The origin of these early 20th century samples is believed to be the Grand Harbour area, at the termination of the largest fluvial catchment in Malta. In contrast, no major fluvial activity occurs in the Latnija area.

The large number of samples from Latnija allows us to obtain robust insights into changing MRE in Malta over time. Chronology variability within contexts may reflect factors such as bioturbation and human activity at the site (e.g.,[213]), but may also simply reflect slightly different locations from which the shells had been collected, such as more versus less lagoonal. Lagoonal areas would have had higher calcium carbonate content, and therefore the incorporation of more 'old' carbon during growth.

In terms of the assessment of sediment integrity, the sequence and clustering of dates within phases attests to the good stratigraphic character of the site. The oldest *Phorcus* ages in Phase V range from 6609-6080 cal. BC ±129 (8.6-8.1 ka) consistent with the charcoal age model. Calibrated *P. turbinatus* dates from Phase III range in age from 6257-5932 to 5834-5557 cal. BC (~8.3-7.9 ka, to 7.8-7.6 ka). The relatively limited range of variation supports the primary position of the Phase III pit fill, in the Mesolithic Horizon. The calibrated ages of shells from the most pronounced of the *P. turbinatus* tips, from context (029), show very limited variation and ages, centring around 6100-5700 cal BC (~7.7-7.1 ka). The complexities of marine calibration mean that the *P. turbinatus* shells come with rather high levels of chronological uncertainty, but are used here to support the stratigraphic integrity of the site and the charcoal age model presented in Fig. 3.

5. Lithic Analysis

A total of 64 lithics (knapped stone tools) were recovered from the Phase V to III deposits at Latnija, as summarized in Table S14. The assemblage has a clear and consistent character, from raw material selection, to reduction technology, and use/abandonment. This offers the first description of Mesolithic lithic technology in the Maltese islands.

The Latnija lithic assemblage is dominated by the use of limestone for knapping, which is not a phenomenon that has been reported before in the Maltese islands. Previous work on Maltese lithic assemblages, primarily from the Neolithic, has documented the use of both local and imported chert, as well as imported obsidian (for a summary, see[214]). The potential of some Maltese limestone to be used for lithic reduction was highlighted by Groucutt[214], who performed knapping experiments to show its feasibility. While relatively fine-grained,

this is certainly not a high-quality raw material, with knapping errors (such as siret fractures and aberrant terminations) being frequent, and it does not form particularly sharp edges.

At least two basic forms of limestone used for knapping at Latnija are evident. Firstly, beach cobbles and T were commonly used, as indicated by rounded and smooth cortical surfaces (Extended Data Fig. 10, Panel A). In total, 23% of the Phase V to III lithics from the site show this characteristic. Given that not all lithics from such a raw material will show the original package form once cortex has been removed from part of the core, this indicates that beach cobbles are a very common raw material form used at the site. Today sources of similar raw materials can be observed at localities, such as Paradise Bay, just over 600 metres northeast of Latnija.

Other limestone used at the site is consistent with terrestrial sources. This is particularly evident for a group of three flakes from Phase III, which show a distinctive rough, orange cortex (Main text Fig. 4, Panel A, artefacts a and c; Extended Data Fig. 10, Panel A). This cortex is very similar to that on experimentally knapped flakes from an outcrop of Tal-Pitkal member limestone from the Upper Coralline Limestone formation in northern Malta[214]. The Tal-Pitkal member is from close to the top (i.e., the youngest) part of the Maltese geological sequence of Miocene age, and outcrops extensively across northern and western Malta.

We emphasise that the natural rocks at the site take the form of cave-wall collapse, leading to angular clasts of various sizes in the deposits. The rounded forms evidently used for much of lithic reduction are from a coastal source, and do not naturally occur in the site. The rocks selected for lithic reduction are also typically of denser and more homogenous character than the natural rock at the site. We were readily able to distinguish lithics from natural rocks at the site, as the former display the usual suite of features associated with deliberate lithic reduction (striking platforms, bulbs of percussion, dorsal scars, etc), while the latter do not.

Only one exception to the use of limestone occurs in the lithics found in the Phase V to III deposits, in contrast to the overlying deposits where more diverse raw materials, including chert and obsidian, occur in stratigraphically complex deposits that will be described in future publications. This is a single small (22 mm maximum dimension) core from Phase III on a dark brown material that appears to be a fine-grained chert. The source of this material is currently unknown.

Across the different Mesolithic phases of the site, lithic technology appears to have a similar character. Cores were seemingly reduced by the use of a hard hammer technique, using a simple single platform reduction method (Extended Data Fig. 10, b3). Cores were typically worked on their narrow, rather than wide faces. There was very limited platform preparation, with most striking platforms either being cortical or plain (i.e., shaped by a single removal). The frequently cortical character of flakes, and large amount of cortical cover on the small number of cores recovered, indicates that reduction processes at the site were typically short. Most flakes have unidirectional scar patterns, consistent with this simple, single platform, reduction method. Most flakes are squat, with only occasional examples of more laminar proportions being observed.

Very few flakes from Phases V to III at Latnija show evidence of retouch. In fact, the one complete retouched piece has simple 'scraper' retouch along one edge (Extended Data Fig. 10 , b4). The other example is a fragment of a retouched flake. Additionally, few flakes show evidence for use-related damage. Many flakes are broken, but this probably primarily reflects accidental fracture during knapping rather than as a result of use.

Finally, the lithic assemblage can offer some insights into site formation processes and taphonomy. Lithics from Phases V and III appear to generally be fresh, reflecting the predominance of fine sediments and limited post-depositional disturbance. In contrast, the lithics from Phase IV are sometimes more weathered. This may reflect differences in accumulation of clasts and fine sediments within these clast-supported contexts.

The relatively small number of lithics recovered from the Mesolithic horizon may reflect the ephemeral or seasonal character of occupations. However, the abundant faunal remains can certainly be contrasted against the much smaller amount of lithics. An alternative possibility, therefore, is that lithics of better-quality raw material were carefully conserved and were not discarded at the site. It could also be that the excavated areas reflect only certain aspects of site use, and that more abundant lithic remains are located elsewhere.

In summary, the Latnija lithic assemblage shows a consistent character. We interpret it as indicating a simple technological approach, where local raw materials were selected to produce sharp edges, which were then used to a limited extent before being abandoned. The

consistency of this behaviour, and the relatively sparse nature of the lithics at the site, indicate a pattern of repeated relatively ephemeral visits to the site. It appears that lithics were knapped on site. In terms of regional context, the Latnija lithics stand in stark contrast to the contemporary assemblages of the Mediterranean Mesolithic in the region, namely assemblages typically described as Castelnovian in Sicily, and the contemporaneous Capsian of North Africa (see section 1 above for discussion of regional context). Unlike broadly contemporary assemblages in areas like the Maghreb and Italy, Latnija lacks both the sophisticated blank production techniques (such as pressure and indirect percussion) and the product of complex retouched forms (e.g., trapezes). One obvious explanation for the simple character of lithics at Latnija is the low-quality raw material that was used/available. It is, however, interesting to note that in Sardinia, the other best documented example of Mesolithic island occupations in the central/western Mediterranean, the lithics are simple, accompanied by an ephemeral occupation[59]. It is therefore possible that several factors including the demographic and functional play an important role in shaping the lithic assemblage at Latnija. Further experimental studies on Maltese raw materials and, hopefully, the identification of further Mesolithic sites in Malta can help cast light on these issues.

6. Faunal and Isotopic Analyses

6.1 Taxonomy and biogeography

For this study we analysed a total of 955 (NSP; NISP = 692) piece-plotted bones from Phases V–III. This faunal assemblage is dominated by birds and deer, while remains of marine mammals (i.e., seal) and terrestrial microfauna (e.g., rodents, tortoise) occur at low frequencies throughout the sequence (Fig. 4, Table S15). The deer remains are attributed to red deer (*Cervus elaphus*; see below). Given the pre-Neolithic age of the deposits (see section 4 above) and absence of other known wild ungulates in Malta during the terminal Pleistocene/Holocene (see below), it is assumed that all Phase V-III ungulate remains are likely attributable to red deer. The bird and other microfauna remains will be analysed by specialists and the results reported in subsequent papers.

During the Middle to Late Pleistocene in Malta, three distinct size classes of red deer were present, comparable in size to modern roe, fallow, and small red deer[215]. This is in contrast to nearby Sicily, where two species of endemic deer were present: the Sicilian red deer (*C. e.*

siciliae) and the Sicilian fallow deer (*D. carburangelensis*). Both of these species were moderately reduced in size compared to their mainland counterparts, but neither approached the size of the smallest Maltese deer, which is estimated to have had an average adult body mass of ~25 kg (ref.[215]). Fallow deer appears to have gone extinct on Sicily by the San Teodoro-Pianetti faunal stage, dated to ca. 70–32 ka, and does not reappear on the island until its reintroduction, perhaps as early as the Bronze Age[216,217]. In contrast, the Sicilian red deer persisted on the island up until the late glacial period at which point it appears to have been replaced by its larger mainland form[215].

Similarly, red deer remains recovered from the upper layers at Għar Dalam and Neolithic tombs in Malta have been suggested to indicate a replacement of the smaller endemic with the larger mainland form[215,218], though this remains to be properly tested. Interestingly, if true, this would suggest the translocation of deer to the Maltese Islands during the early Holocene, with the findings presented here opening the possibility that this translocation was conducted by pre-Neolithic hunter-gatherers. Other instances of pre-Neolithic translocations have been documented elsewhere, such as that of wild boar to Cyprus some 11,500 years ago[219], and even earlier translocations of marsupials to various Pacific Islands[220,221]. Alternatively, red deer may have had a continuous presence on Malta from the Pleistocene into the Holocene up until their eventual extirpation (see also above), the date of which remains largely unknown, but probably occurred sometime in the early to mid-Holocene. For the present paper we follow the most parsimonious explanation—that an endemic red deer population inhabited Malta until its extinction—while noting that the translocation hypothesis warrants further exploration.

The presence of fallow deer on Malta in the upper red earth layers at Għar Dalam has been suggested (see[215]), though little-to-no data has been presented to support this claim. Nonetheless, considering this claim alongside fluctuating sea levels, the ability of deer to swim considerable distances, and the possibility of deer translocations, it remains plausible that fallow deer reached Malta. To that end, we conducted an analysis of deer remains at Latnija to determine whether they belong to red deer or fallow deer. For this we followed[222], focusing on skeletal elements that exhibit clear diagnostic features for distinguishing the two species: the radii exhibit a posteriorly-facing facet on the proximal epiphysis (Extended Data Fig. 11 Panel A, 1i), the medial-anterior edge of the distal articular epiphysis is almost flat (Extended Data Fig. 11 Panel A, 2i), the medial-posterior edge smooth (Extended Data Fig.

11 Panel A, 2ii), and the lateral-posterior edge angle wide (Extended Data Fig. 11 Panel A, 2iii); the metatarsals have a proximal epiphysis with clearly separated facets (Extended Data Fig. 11 Panel A, 3i) and a large foramen (Extended Data Fig. 11 Panel A, 3ii), and a distal epiphysis with long extensions of the articular surface (Extended Data Fig. 11 Panel A, 6vi) and a visible central suture (Extended Data Fig. 11 Panel A, 6v); metacarpals exhibit a clear gap between the posterior of the medial and lateral facets (Extended Data Fig. 11 Panel A, 4i); and the scapulae have a tuberosity that is only slightly downturned (Extended Data Fig. 11 Panel A, 5i), squared (Extended Data Fig. 11 Panel A, 5ii), and a rounded glenoid fossa (Extended Data Fig. 11 Panel A, 5iii).

All of these features are more characteristic of red than fallow deer. This suggests that red deer were present in Malta during the early Holocene, though again, whether this represents a continuous population from the Pleistocene or a translocation by people remains to be tested.

6.2: Species abundance

In Phase V, red deer are the most abundant taxon (62% of NISP), followed by birds (32%), and small numbers of fox (2%), rodents (2%), reptiles (1%), and, notably, a proximal phalanx and metapodial belonging to seal (1%; Table S15). Moving up the sequence into Phase IV, which represents a period of cave wall collapse, red deer (58%) and birds (37%) are still the dominant taxa, though with a slight decrease in deer and increase in birds, and marine mammals (2%), reptiles (1%), and rodents (1%) are again present in small numbers. In Phase III, bird (63%) replace deer (31%) as the dominant taxon, marine mammals (<1%), reptiles (3%), and rodents (<1%) are again present in small numbers, whereas suids make their first appearance at the site (2%). While still under study, the overlying Phase II clearly marks a radical shift in faunal representation at the site, which includes the introduction of domesticates like ovicaprids and cattle to the Maltese Islands, as well as a drastic reduction in birds at the site.

At the time of writing, over ten thousand marine shells have been recovered and we report here the complete counts of marine microfauna for squares L2 and N2 (NISP = 1754 for Phases V–III). *Phorcus turbinatus* is the dominant taxon of marine mollusc and is particularly abundant in Phase III (NISP = 1318) where it occurs in large discrete dumps (see section 2 above). Limpets are consistently present throughout the phases in low numbers, as are

remains of crab (e.g., claws, carapace), fish (e.g., vertebrae), and sea urchins (e.g., spines, tests; Table S16). The abundance of marine invertebrates at Latnija highlights the importance of marine resources in the diets of Mesolithic people in Malta, differing from the stricter focus on terrestrial resources in later periods[48,223,224]. In addition to the marine shells, terrestrial shells are also abundant albeit highly fragmented, and species include the chocolate-band, decollate, and Cliveden snail.

6.3 Preliminary taphonomic analysis

A preliminary taphonomic analysis of a subsample of 439 piece-plotted bone from Phase V offers some insights into the site formation processes and anthropogenic activity at Latnija. Firstly, the analysis has been hindered by poor cortical surface readability, with approximately a third of specimens being covered by sediment/concretions and having low cortical readability (<1/2 of the cortical surface visible) (Extended Data Fig. 11 Panel A, 12–14). Burning is the most commonly observable bone surface modification, with a total of 113 charred and two calcined specimens, or about 25% of the subsample (Extended Data Fig. 11 Panel A, 9–11). Many midshaft fragments exhibit oblique (n = 31), curved (n = 19), and smooth (n = 34) fracture patterns, indicative of being broken while fresh (e.g., Extended Data Fig. 11 Panel A, 7-8). A few midshaft fragments exhibit notches, such as the one presented in Extended Data Fig. 11 Panel A7, which displays a double notch, negative flake scarring, and a green fracture, consistent with this modification being an anthropogenic percussion mark. Twenty-nine carnivore tooth-marked bones have so far been identified from Phase V—which notably contains the fox remains—and include tooth pits, scores, and furrowing of spongy bone on a variety of red deer and bird remains. Finally, 51 (11%) bones exhibit root etching suggesting the presence of vegetation growth at the site, as also occurs today (see Fig. 1).

Taken together, the high degree of charring, fresh breakage patterns, as well as the probable butchery marks, strongly suggest that the Latnija faunal assemblage, or part thereof, was accumulated by people occupying the cave. Raptors may have contributed to the accumulation of microfauna (e.g., rodents, fish) at the site, and the presence of foxes and gnaw marked bones suggest that they too may have contributed or at least, in the case of larger animals like deer and marine mammals, secondarily scavenged from human refuse; though the low proportions of carnivore remains and minimal incidences of gnawing suggest a minor role of carnivores in the accumulation. The red deer and marine mammals were

almost certainly accumulated by people as these are far too large to have been accumulated by the Maltese carnivores, the largest known carnivore during the early Holocene being red fox. Ongoing cleaning, processing, and analysis of the fossil material will provide further insights into the site formation processes, including the specific site use and subsistence behaviours employed by the people living there.

6.4: Isotopic Analyses

Stable carbon ($\delta^{13}C$) and oxygen ($\delta^{18}O$) isotope analysis of animal tooth enamel has emerged as a widely applied tool for studying palaeoenvironmental change (e.g.,[225,226]). In terrestrial ecosystems, the main source of $\delta^{13}C$ variation is a result of differential fractionation of ^{13}C by plants during photosynthesis and the fixation of CO_2 (ref.[227–229]). In contemporary ecosystems, C_3 $\delta^{13}C$ values vary from about -24 to -36‰ while C_4 values range from approximately -9 to -17‰ (ref.[228]). Within C_3 plants, environmental factors such as aridity and temperature can further influence $\delta^{13}C$ values. In contemporary arid and semi-arid regions with annual rainfall below 500 mm of rainfall per year—as is often the case in Malta[230] – terrestrial C_3 plants have been shown to yield higher $\delta^{13}C$ values, up to about –21‰ (ref.[231,232]). Importantly, this $\delta^{13}C$ variation persists along the food chain, with $\delta^{13}C$ values expressed in herbivore bioapatite reflective of the diets of those animals and, therefore, indirectly, their environments[233–235].

$\delta^{18}O$ values of animal tissues can provide additional palaeoecological information about water and food. Precipitation $\delta^{18}O$ is influenced by a variety of factors including the source of rainfall, precipitation amount (amount effect), distance from the coast (continental effect), altitude, and temperature[236,237]. Vegetation $\delta^{18}O$ is reflective of this variation but shows additional variation as a result of evapotranspiration mediated by local climatic impacts[238–240]. Animals which obtain the majority of their water requirements from plants will strongly reflect those environmental factors that influence plant transpiration and plant $\delta^{18}O$ (ref.[241–243]).

Red deer are opportunistic feeders, having a mixed diet that can adapt to a wide range of browse types depending on environmental context[244]. Researchers have found that Corsican red deer—a subspecies of red deer native to nearby Corsica and Sardinia—selectively browse on holm oak (*Quercus ilex*) and green olive trees (*Phillyrea latifolia*)[244], two species native to the Maltese Islands. Wood mouse is primarily granivorous, though will also consume the

vegetative parts of plants, stems, leaves, and flowers, as well as invertebrates, when seed availability is low[245].

Although this stable isotope analysis is preliminary, and sample size small, the results offer some initial insights into environments at Latnija, particularly during Phase V, dated to ~8.6–7.8 ka (Table S7, Extended Data Fig. 11 Panel B). The $\delta^{13}C$ and $\delta^{18}O$ values range from 11.7‰ to -8.1‰ (mean = -9.90‰) and -5.0‰ to -2.4‰ (mean = -3.5‰), respectively (Table S7). Red deer $\delta^{13}C$ values span from -11.7‰ to -9.8‰ (mean = -10.7‰), while their $\delta^{18}O$ values range from -4.4‰ to -2.4‰ (mean = -3.2‰). Wood mouse $\delta^{13}C$ values range from -10.9‰ to -8.1‰ (mean = -9.5‰), while wood mouse $\delta^{18}O$ values span from -5‰ to -2.4‰ (mean = -3.7‰). Overall, these data suggest a dominance of C_3 ecosystems in Malta during the Mesolithic occupation of the site, with perhaps some small input of C_4 resources into the diets of wood mouse. That said, slightly higher values in these taxa could also be associated with the impacts of aridity on C_3 plants in more evaporative settings.

7. Supplementary Tables

Latnija 2021 and 2022				Charred material												Uncharred	
						Amaranthaceae	Caryophyllaceae	Euphorbiaceae	Fabaceae		Malvaceae	Poaceae		**Totals without charcoal**	**Totals with charcoal**	Faunal remains	
Year		Bag number	Phase	**Snails**	Charcoal >2mm	Chenopodioideae	*Vaccaria hispanica*	*Mercurialis* cf. *annua*	Fabaceae	*Vicia* (wild)	*Malva* sp.	Poaceae	unidentifiable			Snails	Small bones
2022	N3	19	III		1	2	2		1	2				7	8	311	28
2022	N3	25	III		162			4		1				5	167	545	27
2022	N3	29	III		554	4	3	5		3				15	569	185	62
2021	N2	25	III	2	1	1		1	3	1	4			10	11	98	8
2021	N2	29	III		114				3			33	4	40	154	2	
2021	L2	29	III		2									0	2	83	105
2021	L2	31	III		50									0	50	80	270
2021	N2	37	III		3							2		2	5	1	
2021	L2	28	IV		52		1			1		4	4	10	62	39	3
2022	N3	100	IV	1	111		4			4				8	119	169	5
2022	N3	102	IV	2	10	1		3		2			2	8	18	621	29
2022	M3	103	IV		32								1	1	33	191	1
2022	L3	104	IV		42									0	42	652	1
2021	L2	32	V		11									0	11	175	82
2021	L2	34	V		461				7	4		74	3	88	549	7	2
2021	L2	34	V		25					2				2	27	77	460
2021	L2	36	V		5					2				2	7	310	71
2021	L2	38	V	1	193							9	1	10	203	15	2
2021	L2	39	V		2									0	2	17	29
2021	L2	40	V		4									0	4	84	52
2021	L2	41	V		5									0	5	115	290
2021	L2	44	V		23									0	23	13	40
2021	L2	45	V		11									0	11	153	245
2021	L2	46	V											0	0	14	14
2021	L2	47	V		1									0	1	54	14
2021	L2	48	V		76					4				4	80	382	137
2021	N2	43	V		28									0	28	9	
2022	L3	106	V		36	2							2	4	40	270	1
2022	M3	106	V		10			4					2	6	16	248	
			Sum	6	2025	10	10	17	14	26	4	122	19	222	2247	4920	1978

Table S1. Floated remains from the 2021 and 2022 field seasons. Note charred material is separated from non-charred material. The floated material was particularly analysed for archaeobotanical remains, shown here in detail.

Lab No. MAMS	Square	Context	Phase	14C Age (UnCal)yr BP	±	13C AMS	Probability 68%<br><br>Cal BC	Probability 95%<br><br>Cal BC	C [%]
61861	N3	19	III	6929	27	-25	5836-5756	5886-5731	68.3
61857	N3	25	III	6956	27	-24	5886-5785	5967-5741	69
54779	N2	25	III	5510	30	-23.6	4440-4332	4446-4268	66.9
61851	N3	29	III	6918	22	-21.6	5830-5744	5877-5731	65.2
54774	N2	29	III	7041	32	-18.4	5983-5892	5993-5841	63.8
61854	N3	31	III	6984	26	-20.6	5969-5833	5979-5776	69.4
54781	N2	37	III	6871	35	-25.3	5794-5718	5839-5669	65.3
61846	L3	104	IV	6908	21	-30.3	5802-5736	5842-5727	60
54768	L2	28	IV	6997	32	-16.3	5971-5842	5983-5787	65.2
54765	L2	34	V	7096	34	-34.3	6015-5921	6059-5893	67.6
54771	L2	38	V	7058	33	-18.5	5987-5899	6015-5846	64.1
62546	N2	39	V	7078	27	-29.8	6007-5915	6018-5894	63.4
62547	N2	40	V	7081	26	-26.4	6007-5917	6019-5895	64.5
62553	L2	41	V	7008	26	-25.3	5974-5846	5984-5804	66.2
54769	N2	43	V	7229	33	-19.1	6211-6024	6220-6016	65.4
62552	L2	44	V	6970	26	-25.8	5891-5799	5971-5755	66.8
62548	N2	46	V	7375	27	-28.8	6351-6097	6372-6086	61.8
62549	N2	47	V	7112	26	-26.1	6019-5927	6061-5916	63.4
54780	L2	48	V	6998	35	-26.5	5973-5841	5985-5781	66.9
65878	LM3	143	V	6903	19	-21.5	5796-5736	5837-5728	72.3

65862	M3	167	V	7231	19	-20.8	6087-6029	6218-6022	71.9
65877	M3	168	V	7504	20	-21.4	6423-6277	6433-6260	57.1
62551	N2	51	VI	9917	31	-23.2	9441-9305	9511-9288	61.6
65864	L3	172	VI	9018	21	-21.7	8276-8246	8286-8231	70.8
65863	M3	175	VI	7653	20	-20	6500-6455	6570-6437	71.6
65865	L3	177	VI	7367	19	-23.1	6329-6099	6357-6086	72.7
65866	L3	182	VI	5160	17	-19.8	3982-3959	4039-3951	72
65867	L3	183	VI	7740	20	-21	6598-6508	6641-6481	69.7
65868	M3	184	VI	7715	20	-22.6	6588-6503	6596-6473	71
65869	M3	186	VI	9238	22	-19.5	8544-8356	8554-8340	60.1
65879	N3	190	VI	6932	19	-20.9	5837-5764	5883-5737	72.9
65874	MN3	198	VI	7068	20	-22.8	5989-5914	6010-5894	67.4
**71684**	**N3**	**171**	**V**	**n/a**					
**71685**	**N3**	**166**	**V**	**na**					
**71686**	**M3**	**162**	**V**	**n/a**					
**71687**	**L3**	**161**	**V**	**n/a**					
**71688**	**L3**	**143**	**V**	**6100**	**30**		**5195-4955**	**5205-4910**	
**71689**	**M3**	**143**	**V**	**n/a**					

Table S2. Radiocarbon dates and calibrated ages used to calculate the age model. Calibrated with Oxcal 4.4. with the IntCal20 dataset. Dates in bold represent bone, whereas non-bold represent charcoal samples. Most bone dates failed due to a lack of collagen in the samples.

Overall Agreement								76.9
Model Agreement								79.1
			Modelled	Unmodelled		Modelled		
Name	Type	Expected Age	HDI (95%)	Lower 95%	Upper 95%	Lower 95%	Upper 95%	Agree
U	number	-0.04	4.7	-2.55	2.5	-2.4	2.3	
T	number	1.46	3.32	0.04	3.76	0.02	3.34	100
General	interval	-6.82	280			-155	125	
VIA_st	date	-13380.44	6475			-16999.5	-10524.5	
62551	date	-11318.95	215	-11394.5	-11239.5	-11454.5	-11239.5	100.6
VA_st	date	-8117.3	385			-8344.5	-7959.5	
62546	date	-7907.26	115	-7964.5	-7844.5	-7964.5	-7849.5	100.2
62547	date	-7909.22	115	-7964.5	-7844.5	-7964.5	-7849.5	99.9
54769	date	-8027.45	195	-8164.5	-7969.5	-8159.5	-7964.5	110
62548	date	-8114.82	255	-8314.5	-8039.5	-8284.5	-8029.5	85.2
62549	date	-7936.58	135	-8004.5	-7869.5	-8004.5	-7869.5	101.8
=IV_st	date	-7835.46	155			-7919.5	-7764.5	
VIB_st	date	-11490.22	3460			-13479.5	-10019.5	
65879	date	-7752.4	140	-7829.5	-7689.5	-7829.5	-7689.5	100.4
65863	date	-8435.06	125	-8514.5	-8389.5	-8514.5	-8389.5	99.7
65874	date	-7896.49	110	-7954.5	-7844.5	-7954.5	-7844.5	99.6

65869	date	-10400.21	205	-10499.5	-10294.5	-10499.5	-10294.5	100.1
65868	date	-8488.82	115	-8539.5	-8429.5	-8539.5	-8424.5	100.7
65864	date	-10206.34	45	-10229.5	-10184.5	-10229.5	-10184.5	100.2
65865	date	-8187.7	275	-8314.5	-8039.5	-8314.5	-8039.5	99.5
65867	date	-8509.77	135	-8584.5	-8434.5	-8584.5	-8449.5	100.4
65866	date	-5925.24	85	-5984.5	-5904.5	-5984.5	-5899.5	99
VB_st	date	-8467.05	465			-8714.5	-8249.5	
54771	date	-7891.21	160	-7959.5	-7799.5	-7959.5	-7799.5	101.4
54765	date	-7921.52	155	-8004.5	-7844.5	-8004.5	-7849.5	100.1
62553	date	-7856.81	145	-7929.5	-7754.5	-7929.5	-7784.5	101.8
62552	date	-7808.67	200	-7914.5	-7704.5	-7919.5	-7719.5	100.9
54780	date	-7843.79	185	-7929.5	-7734.5	-7929.5	-7744.5	102.5
65878	date	-7730.28	105	-7779.5	-7679.5	-7784.5	-7679.5	97.4
71688	date	-8996.91	335	-9119.5	-8989.5	-9119.5	-8784.5	97.1
65862	date	-8038.52	190	-8164.5	-7974.5	-8164.5	-7974.5	98.9
65877	date	-8314.9	170	-8379.5	-8214.5	-8379.5	-8209.5	97.2
=IV_st	date	-7835.46	155			-7919.5	-7764.5	
IV_st	date	-7835.46	155			-7919.5	-7764.5	
54768	date	-7810.71	160	-7929.5	-7739.5	-7904.5	-7744.5	104.6
61846	date	-7779.91	115	-7784.5	-7679.5	-7834.5	-7719.5	57.1
IIIA_st	date	-7780.56	95			-7829.5	-7734.5	

61851	date	-7742.35	100	-7819.5	-7679.5	-7789.5	-7689.5	103.8
54774	date	-7802.59	180	-7939.5	-7794.5	-7899.5	-7719.5	49.6
61854	date	-7776.34	135	-7924.5	-7729.5	-7839.5	-7704.5	84.2
54781	date	-7726.11	130	-7784.5	-7619.5	-7789.5	-7659.5	88.7
IIIB_st	date	-7677.45	290			-7789.5	-7499.5	
61861	date	-7748.54	145	-7829.5	-7684.5	-7829.5	-7684.5	101.3
61857	date	-7777.12	220	-7909.5	-7694.5	-7909.5	-7689.5	100.7
54779	date	-6316.17	170	-6389.5	-6219.5	-6389.5	-6219.5	98.8
IIIB_en	date	-5745.52	3570			-7039.5	-3469.5	

Table S3. OxCal modelling results. HDI refers to Highest Density Interval. The first three parameters (U, T, General) refer to the Outlier Model (see[57]), which identified none of the modelled events as outliers (all had posterior outlier probabilities of <= 0.05).

Phase	No. charcoal	No. Phorcus	MRE	±
III	6	24	64.2	64.8
IV	1	3	9.5	111.5
V	7	22	-57.3	128.6

Table S4. MRE corrections by Phase.

Phase	Lab No MAMS	14C Age [yr BP]	±	Context	Material	MRE	±	cal BC (95%)
III	61861	6929	27	19	charcoal	64.2	64.8	cal BC 5886-5732
	61745	7811	24	19	shell marine: *Phorcus*			cal BC 6219-5877
	61754	7531	24	19	shell marine: *Phorcus*			cal BC 5974-5628
	61755	7790	24	19	shell marine: *Phorcus*			cal BC 6211-5860
	59275	7563	27	19	shell marine: *Phorcus*			cal BC 6001-5646
	61729	7517	25	19	shell marine: *Phorcus*			cal BC 5966-5621
	61772	7861	22	19	shell marine: *Phorcus*			cal BC 6236-5906
	61857	6956	27	25	charcoal			cal BC 5967-5741
	61724	7380	25	25	shell marine: *Phorcus*			cal BC 5851-5559
	61770	7348	22	25	shell marine: *Phorcus*			cal BC 5834-5557
	54774	7041	32	29	charcoal			cal BC 5994-5841
	61851	6918	22	29	charcoal			cal BC 5877-5731
	61695	7932	25	29	shell marine: *Phorcus*			cal BC 6257-5932
	61735	7625	24	29	shell marine: *Phorcus*			cal BC 6062-5703
	61737	7511	23	29	shell marine: *Phorcus*			cal BC 5961-5618
	61740	7776	24	29	shell marine: *Phorcus*			cal BC 6201-5843
	61752	7479	25	29	shell marine: *Phorcus*			cal BC 5929-5591
	59273	7801	28	29	shell marine: *Phorcus*			cal BC 6215-5865
	61713	7600	22	29	shell marine: *Phorcus*			cal BC 6036-5676
	61719	7450	25	29	shell marine: *Phorcus*			cal BC 5901-5581
	61769	7454	21	29	shell marine: *Phorcus*			cal BC 5903-5581
	61773	7477	21	29	shell marine: *Phorcus*			cal BC 5925-5591
	61807	7539	19	29	shell marine: *Phorcus*			cal BC 5977-5633
	61809	7681	20	29	shell marine: *Phorcus*			cal BC 6118-5741
	61810	7765	20	29	shell marine: *Phorcus*			cal BC 6197-5837
	61811	7865	21	29	shell marine: *Phorcus*			cal BC 6240-5909
	61854	6984	26	31	charcoal			cal BC 5979-5779
	61813	7655	20	31	shell marine: *Phorcus*			cal BC 6082-5721

	54781	6871	35	37	charcoal			cal BC 5841-5672
	61808	7591	19	37	shell marine: *Phorcus*			cal BC 6025-5669
IV	54768	6997	32	28	charcoal	9.5	111.5	cal BC 5983-5790
	61764	7905	26	28	shell marine: *Phorcus*			cal BC 6466-5956
	61781	7647	25	28	shell marine: *Phorcus*			cal BC 6218-5722
	61784	7404	21	28	shell marine: *Phorcus*			cal BC 5979-5526
V	54765	7096	34	34	charcoal	-57.3	128.6	cal BC 6058-5894
	61751	7592	25	34	shell marine: *Phorcus*			cal BC 6236-5671
	61760	7467	23	34	shell marine: *Phorcus*			cal BC 6099-5555
	61708	7790	23	34	shell marine: *Phorcus*			cal BC 6442-5884
	61790	7245	24	34	shell marine: *Phorcus*			cal BC 5951-5462
	61792	7419	23	34	shell marine: *Phorcus*			cal BC 6056-5536
	61806	8091	20	34	shell marine: *Phorcus*			cal BC 6609-6080
	62546	7078	27	39	charcoal			cal BC 6017-5895
	61734	7750	24	39	shell marine: *Phorcus*			cal BC 6410-5843
	61714	7685	22	39	shell marine: *Phorcus*			cal BC 6356-5775
	61780	7708	26	39	shell marine: *Phorcus*			cal BC 6376-5801
	62547	7081	26	40	charcoal			cal BC 6019-5896
	61736	7406	23	40	shell marine: *Phorcus*			cal BC 6043-5528
	62553	7008	26	41	charcoal			cal BC 5985-5806
	61690	7345	23	41	shell marine: *Phorcus*			cal BC 5991-5495
	61696	7971	25	41	shell marine: *Phorcus*			cal BC 6580-6041
	61747	7602	25	41	shell marine: *Phorcus*			cal BC 6248-5680
	61749	7420	27	41	shell marine: *Phorcus*			cal BC 6056-5536
	61797	7339	19	41	shell marine: *Phorcus*			cal BC 5985-5492
	62548	7375	27	46	charcoal			cal BC 6367-6084
	61785	7403	21	46	shell marine: *Phorcus*			cal BC 6038-5526
	61793	7776	19	46	shell marine: *Phorcus*			cal BC 6431-5871
	61801	7906	22	46	shell marine: *Phorcus*			cal BC 6544-5993

	**62549**	**7112**	**26**	**47**	**charcoal**			**cal BC 6060-5916**
	**61783**	**7564**	**22**	**47**	**shell marine: *Phorcus***			**cal BC 6216-5657**
	**61802**	**7586**	**19**	**47**	**shell marine: *Phorcus***			**cal BC 6230-5672**
	**54780**	**6998**	**35**	**48**	**charcoal**			**cal BC 5985-5789**
	**61777**	**7544**	**21**	**48**	**shell marine: *Phorcus***			**cal BC 6200-5641**
	**61743**	**7449**	**25**	**48**	**shell marine: *Phorcus***			**cal BC 6081-5546**

Table S5. *P. Turbinatus* dates with charcoal ages organized by Phase. The results of the calibration per phase are shown in the last column on the right-hand side. Note results are calculated in cal. B.C.

Sample ID	Prior	Posterior	Model	Type
62551	5	4	General	t
62546	5	4	General	t
62547	5	4	General	t
54769	5	4	General	t
62548	5	4	General	t
62549	5	4	General	t
65879	5	4	General	t
65863	5	4	General	t
65874	5	4	General	t
65869	5	4	General	t
65868	5	4	General	t
65864	5	4	General	t
65865	5	4	General	t
65867	5	4	General	t
65866	5	4	General	t
54771	5	4	General	t
54765	5	4	General	t
62553	5	4	General	t
62552	5	4	General	t
54780	5	4	General	t
65878	5	4	General	t
71688	5	7	General	t
65862	5	4	General	t
65877	5	4	General	t
54768	5	4	General	t

61846	5	6	General	t
61851	5	4	General	t
54774	5	8	General	t
61854	5	4	General	t
54781	5	4	General	t
61861	5	4	General	t
61857	5	4	General	t
54779	5	4	General	t

Table S6. Outlier modelling results from OxCal 4.4.

Taxon	Phase	δ13C	S.D.	δ18O	S.D.
Cervus elaphus	3	-9.8	0.3	-2.5	0.2
Cervus elaphus	5	-11.7	0.2	-2.4	0.1
Cervus elaphus	5	-11.2	0.2	-3.5	0.1
Cervus elaphus	5	-11.1	0.1	-2.9	0.1
Cervus elaphus	5	-10.9	0.2	-3.1	0.1
Cervus elaphus	5	-10.1	0.1	-4.4	0.1
Cervus elaphus	5	-9.9	0.2	-3.6	0.0
Apodemus sylvaticus	5	-10.9	0.2	-2.4	0.1
Apodemus sylvaticus	5	-10.5	0.1	-2.5	0.0
Apodemus sylvaticus	5	-10.3	0.2	-3.0	0.1
Apodemus sylvaticus	5	-9.9	0.2	-4.0	0.2
Apodemus sylvaticus	5	-9.8	0.2	-4.4	0.1
Apodemus sylvaticus	5	-9.8	0.2	-3.4	0.1
Apodemus sylvaticus	5	-9.3	0.1	-4.6	0.0
Apodemus sylvaticus	5	-9.2	0.2	-2.8	0.1
Apodemus sylvaticus	5	-9.0	0.1	-5.0	0.1
Apodemus sylvaticus	5	-8.6	0.2	-3.4	0.2
Apodemus sylvaticus	5	-8.2	0.2	-4.0	0.2
Apodemus sylvaticus	5	-8.1	0.1	-4.4	0.1

Table S7. Raw $\delta^{13}C$ and $\delta^{18}O$ data.

Facies	Age range (ka)	Sicily dates (ka)	Sardinia dates (ka)	Main features
Sauveterrian-like	11.6-8.6	9.8-9.4		Small flakes and blades, hard hammer percussion, abundant microliths, double backed points, segments, triangles, end scrapers.
Undifferentiated Epipalaeolithic	11.2-8.0	10.1-9.1	10.1-8.1	Low technical investment. Wide unstandardized flakes. Hard hammer percussion. Side scrapers, denticulates, etc. Rare microliths.
Epiromanellian	11.7-9.9			Small flakes and blades, circular end scrapers, single backed tools, low rate of geometrics.
Epigravettian-tradition	11.0-9.1	10.7-9.3		Small flakes and blades, single backed tools, triangles and crescent, long end-scrapers.
Trapeze facies (Castelnovian)	9.2-7.7	9.0-8.0		Small bladelets, pressure flaking and indirect percussion, trapezes, notched blades, long end-scrapers.

Table S8. Ages and characteristics of the main discussed Mesolithic facies in central-southern Italy and adjacent islands (information from[11]).

**Region**	**Phase**	**Boundary**	**expected_age**	**From (95.4%) cal. BC**	**To (95.4%) cal. BC**	**Boundary Variant**
Central_Italy	Meso	End	-8727	-9175	-8250	sigma
Central_Italy	Meso	End	-7767	-7955	-7510	uniform
Central_Italy	Neo	Start	-7552	-7600	-7510	sigma
Central_Italy	Neo	Start	-7698	-7735	-7670	uniform
Corsica	Meso	End	-8541	-8850	-8220	sigma
Corsica	Meso	End	-8138	-8375	-7880	uniform
Corsica	Neo	Start	-7505	-7625	-7390	sigma
Corsica	Neo	Start	-7662	-7780	-7580	uniform
Malta	Neo	Start	-7240	-7355	-7140	sigma
Malta	Neo	Start	-7348	-7460	-7240	uniform
Northern_Italy	Meso	End	-7743	-7965	-7510	sigma
Northern_Italy	Meso	End	-6775	-6920	-6635	uniform
Northern_Italy	Neo	Start	-7420	-7460	-7385	sigma
Northern_Italy	Neo	Start	-7665	-7695	-7630	uniform
Sardinia	Meso	End	-8440	-8775	-8075	sigma
Sardinia	Meso	End	-7889	-8150	-7645	uniform
Sardinia	Neo	Start	-7507	-7605	-7420	sigma
Sardinia	Neo	Start	-7611	-7705	-7525	uniform
SicilyAlt	Meso	End	-8336	-8660	-8000	sigma
SicilyAlt	Meso	End	-7898	-8115	-7700	uniform
SicilyAlt	Neo	Start	-7676	-7830	-7540	sigma
SicilyAlt	Neo	Start	-7930	-8035	-7845	uniform
Sicily	Meso	End	-8160	-8495	-7815	sigma
Sicily	Meso	End	-7411	-7630	-7175	uniform
Sicily	Neo	Start	-7603	-7750	-7465	sigma
Sicily	Neo	Start	-7848	-7975	-7725	uniform
Southern_Italy	Meso	End	-8295	-8730	-7825	sigma
Southern_Italy	Meso	End	-7741	-7935	-7500	uniform
Southern_Italy	Neo	Start	-7675	-7715	-7640	sigma
Southern_Italy	Neo	Start	-7865	-7935	-7805	uniform

Table S9. 95% Credible Intervals for the posterior densities of OxCal phase model start of Neolithic and end of Mesolithic dates by region and model boundary variant. Note that SicilyAlt refers to the model in which in which a few ambiguous samples were experimentally labelled as Neolithic, rather than Mesolithic.

Taxa	III	IV	V
Cistaceae			1
Erica sp.	1		
Juniperus/*Tetraclinis*			5
Pistacia cf. *lenstiscus*	16	11	29
Pistacia sp.	73	9	30
cf. Fabaceae			1
cf. *Pistacia*	2	1	15
cf. *Rhamnus*			1
Angiosperm	5	3	12
Conifer			2
Undetermined	6	2	6
Total	37	26	102

Table S10. Results of the identification of the charcoal remains from Latnija.

Sample	61	62	63	64	65	66	67	68	69	70	71	72	73	74	75	76	77	78	79	80	81	82	83	84
Layer	C	C	C	C	C	C	TI	TI	TI	TI	TI	TI	TI	TI	CR	CR	CR	CR	CR	CR	CR	CR	CR	CR
Bulliform flabellate	1			0	0				0		1				1		1							
Crenate	5	1									1							1		2	5		2	
Cylindroid entire	1																							
Polygonal acicular														2		5	4		1					
Acicular	3																							
Elongate dentate	8	1									4		1				3			1	26			7
Elongate entire	34	3	3				1	1		1	12	8	2	7	11	9	7	9	8	6	65	7	6	10
Elongate sinuate	1	4	1					1			3				5		1				6			3
Elongate clavate											1										1			
Elongate crenate	3	1																						
Papillate	3												1			1								
Blocky	13	1	3								1	2	1					1		2	6			1
Tabular entire	13		2								1	2	1	1		1				1	12	1	2	
Polygonal entire	1										1													
GSSCP Bilobate	16	2									1	1		1		1				1	17		1	2
GSSCP Cross	2										1													
GSSCP Polylobate	6		1																		4			3
GSSCP Rondel	64	9	2								2	2	1	1	1	1					41	2	2	9
GSSCP Trapezoid	2																							
Hair base												1												
Amoeboid clavate	6	5								1	2	5			8	37	11	10	10	4	19	7	12	9
Acute bulbosus	3										1	1				1	1			1	6			
Spheroid echinate	2	1					1				2	13			69	1	28	15	28	28	26	36	48	32
Spheroid clavate										1														
Tracheary			1								1	1												
MC Amoeboid clavate												8												
MC Blocky														2										
MC Elongate clavate		2												2										
MC Elongate crenate																					2			
MC Elongate entire	19	6																						
MC Elongate entire											7	3	5						3		23	2		8
MC Elongate dentate			2									5		3			3						2	
MC Polygonal entire	4																							
MC GSSCP Rondel		7																						
MC Tracheary annulate												7												
MC Amoeboid clavate																5								
MC Amoeboid clavete & Stomata	5																							
MC Elongate dentate & Crenate	4																							
MC Elongate dentate & Stomata																							3	
MC Elongate entire & Crenate	5																4							
Weathered morphotypes	37	13	5	0	0					1	6	15	1			4	2	1		1	7	1		1
Melted morphotypes	2										3													
**Total identified phytoliths**	263	56	20	0	0	0	2	2	0	4	51	74	13	19	95	66	65	37	50	47	266	56	78	85

Table S11. Raw data obtained from phytolith analysis of the basal Phase V hearth in square N2. Key: C, Control; TI, Thermal Impact; CR, Combustion Residue.

Sample	Layer	Main mineral composition	Clay	Calcite origin
61	C	Ca, Cl (nb), Qz	Non burned	Geogenic calcite
62	C	Ca, Cl (nb), Qz	Non burned	Geogenic calcite
63	C	Ca, Cl (nb), Qz	Non burned	Geogenic calcite
64	C	Ca, Cl (nb), Qz	Non burned	Geogenic calcite
65	C	Ca=Cl (nb), Qz	Non burned	Geogenic calcite
66	C	Ca, Cl (nb), Qz	Non burned	Geogenic calcite
67	TI	Ca, Cl (nb), Qz	Non burned	Pyrogenic calcite
68	TI	Ca, Cl (nb), Qz	Non burned	Geogenic calcite
69	TI	Ca, Cl (nb), Qz	Non burned	Geogenic calcite
70	TI	Ca, Cl (nb), Qz	Non burned	Pyrogenic calcite
71	TI	Ca, Cl (nb), Qz	Non burned	Geogenic calcite
72	TI	Ca, Cl (nb), Qz	Non burned	Geogenic calcite
73	TI	Ca, Cl (nb), Qz	Non burned	Geogenic calcite
74	TI	Ca, Cl (nb), Qz	Non burned	Pyrogenic calcite
75	CR	Ca, Cl (nb), Qz	Non burned	Pyrogenic calcite
76	CR	Ca=Cl (nb), Qz	Non burned	Geogenic calcite
77	CR	Ca, Cl (nb), Qz	Non burned	Pyrogenic calcite
78	CR	Ca, Cl (nb), Qz	Non burned	Pyrogenic calcite
79	CR	Ca, Cl (nb), Qz	Non burned	Pyrogenic calcite
80	CR	Ca, Cl (nb), Qz	Non burned	Pyrogenic calcite
81	CR	Ca, Cl* (nb), Qz	Burned	Geogenic calcite
82	CR	Ca, Cl (b), Qz	Burned	Pyrogenic calcite
83	CR	Ca, Cl (b), Qz	Burned	Pyrogenic calcite
84	CR	Ca, Cl (b), Qz	Burned	Pyrogenic calcite

Table S12. Results of FTIR analysis. Key: C, Control; TI, Thermal Impact; CR, Combustion Residue; Ca, Calcite; Cl, Clay; Qz, quartz; b, thermally altered clay; nb, not thermally altered clay. Minerals are arranged according to their relative peak heights in the infrared spectra.

**Lab No. MAMS**	**Square**	**Context**	**Species**
65878	LM3	143	*Pistacia* sp.
65862	M3	167	*Pistacia* sp.
65864	L3	172	*Pistacia* sp.
65863	M3	175	angiosperm
65865	L3	177	*Juniperus/Tetraclinis*
65866	L3	182	*Juniperus/Tetraclinis*
65867	L3	183	angiosperm
65868	M3	184	*Juniperus/Tetraclinis*
65879	N3	190	cf. *Pistacia*
65874	MN3	198	angiosperm

Table S13. List of identified wood charcoal used in the Age Model.

	Flake	Retouched	Core	Total
**Phase V**	19 (90%)	1 (5%)	1 (5%)	21
**Phase IV**	13 (93%)	/	1 (7%)	14
**Phase III**	25 (86%)	1 (3%)	2 (7%)	29

Table S14. Composition of lithic assemblage by phase (total counts and % values).

Class	Family	Taxon	Common name	III	IV	V	Total
Aves							
		Gen. et sp. indet.	Bird	175 (55%)	28 (25%)	108 (20%)	**316**
Reptilia							
	Testudinidae	Gen. et sp. indet.	Tortoise	7 (2%)	1 (1%)	4 (1%)	**14**
	Lacertidae	Gen. et sp. indet.	Lizard	1 (<1%)	0 (0%)	0 (0%)	**1**
Mammalia							
	Muridae	*Apodemus sylvaticus*	Wood mouse	2 (1%)	1 (<1%)	2 (<1%)	**5**
		Gen. et sp. Indet.	Rodent	0 (0%)	0 (0%)	4 (1%)	**4**
	Ungulata	Gen. et sp. Indet.	Medium-sized ungulate	58 (18%)	38 (31%)	161 (30%)	**257**
	Cervidae	*Cervus elaphus*	Red deer	28 (9%)	6 (5%)	50 (9%)	**84**
	Suidae	*Sus* sp.	Suid	5 (2%)	0 (0%)	0 (0%)	**5**
	Canidae	*Vulpes Vulpes*	Red fox	0 (0%)	0 (0%)	8 (2%)	**8**
	Phocidae	Gen. et sp. indet.	Seal	1 (<1%)	2 (2%)	2 (<1%)	**5**
Indet.			Indet.	39 (12%)	34 (30%)	190 (36%)	**268**
**Total**				**316**	**110**	**529**	**955**

Table S15. NRSP (%NRSP) of piece-plotted finds by taxon and phase.

**Taxon**	**III**	**IV**	**V**	**Total**
Phorcus turbinatus	1318 (98%)	60 (80%)	328 (91%)	**1706**
Limpet (*Patella* sp.)	18 (1%)	15 (20%)	15 (4%)	**56**
Crab	1 (<1%)	/	6 (2%)	**7**
Fish	2 (<1%)	/(%)	7 (2%)	**9**
Sea urchin	3 (<1%)	/	5 (1%)	**11**
**Total**	**1342**	**75**	**361**	**1789**

Table S16. NSP (%NSP) of marine invertebrates and fish recovered during excavation and from wet-sieving and flotation of sediments from squares L2 and N2.

8. OxCal Scripts

Latnija Main

```
Plot()
{
	Outlier_Model("General",T(5),U(0,4),"t");
	Sequence("LAT")
	{
	Phase("VI_V")
	{
	Sequence("VIA_VA")
	{
		Sigma_Boundary("VIA_st");
		//Phase VIA
		//051
		R_Date("62551",9917,31){
		Outlier("General",0.05);
		};
		Sigma_Boundary("VA_st");
		Phase("VA")
		{
		//039
		R_Date("62546",7078,27){
		Outlier("General",0.05);
		};
		//040
		R_Date("62547",7081,26){
		Outlier("General",0.05);
		};
		//043
		R_Date("54769",7229,33){
		Outlier("General",0.05);
		};
		//046
		R_Date("62548",7375,27){
		Outlier("General",0.05);
		};
		//047
		R_Date("62549",7112,26){
		Outlier("General",0.05);
		};
		};
		Sigma_Boundary("=IV_st");
	};
	Sequence("VIB_VB")
	{
		Sigma_Boundary("VIB_st");
		Phase("VIB")
		{
```

```
//190
R_Date("65879",6932,19){
Outlier("General",0.05);
};
//175
R_Date("65863",7653,20){
Outlier("General",0.05);
};
//198
R_Date("65874",7068,20){
Outlier("General",0.05);
};
//186
R_Date("65869",9238,22){
Outlier("General",0.05);
};
//184
R_Date("65868",7715,20){
Outlier("General",0.05);
};
//172
R_Date("65864",9018,21){
Outlier("General",0.05);
};
//177
R_Date("65865",7376,19){
Outlier("General",0.05);
};
//183
R_Date("65867",7740,20){
Outlier("General",0.05);
};
//182
R_Date("65866",5160,17){
Outlier("General",0.05);
};
};
Sigma_Boundary("VB_st");
Phase("VB")
{
//038
R_Date("54771",7058,33){
Outlier("General",0.05);
};
//034
R_Date("54765",7096,34){
Outlier("General",0.05);
};
//041
R_Date("62553",7008,26){
```

```
			Outlier("General",0.05);
			};
			//044
			R_Date("62552",6970,26){
			Outlier("General",0.05);
			};
			//048
			R_Date("54780",6998,35){
			Outlier("General",0.05);
			};
			//143
			R_Date("65878",6903,19){
			Outlier("General",0.05);
			};
			//143 bone
			R_Date("71688",8100,30){
			Outlier("General",0.05);
			};
			//167
			R_Date("65862",7231,19){
			Outlier("General",0.05);
			};
			//168
			R_Date("65877",7504,20){
			Outlier("General",0.05);
			};
			};
			Sigma_Boundary("=IV_st");
	};
	};
	Sigma_Boundary("IV_st");
	Phase("IV")
	{
	//028
	R_Date("54768",6997,32){
			Outlier("General",0.05);
	};
	//104
	R_Date("61846",6908,21){
			Outlier("General",0.05);
	};
	};
	Sigma_Boundary("IIIA_st");
	Phase("IIIA")
	{
	//029
	R_Date("61851",6918,22){
			Outlier("General",0.05);
	};
	//029
```

```
        R_Date("54774",7041,32){
                Outlier("General",0.05);
        };
        //031
        R_Date("61854",6984,26){
                Outlier("General",0.05);
        };
        //037
        R_Date("54781",6871,35){
                Outlier("General",0.05);
        };
        };
        Sigma_Boundary("IIIB_st");
        Phase("IIIB")
        {
        //019
        R_Date("61861",6929,27){
                Outlier("General",0.05);
        };
        //025
        R_Date("61857",6956,27){
                Outlier("General",0.05);
        };
        //025
        R_Date("54779",5510,30){
                Outlier("General",0.05);
        };
        };
        Sigma_Boundary("IIIB_en");
        };
};
```

Regional Meso-Neo Transition (By RegioCultural Phase)

In each RegioCultural Phase, we include the OxCal model with ”Sigma” and then “Uniform” boundaries.

Mesolithic

Central Italy:

```
Plot(){
Sequence("Central_Italy_mesolithic"){
 Sigma_Boundary("Central_Italy_mesolithic_start");
  Phase("Central_Italy_mesolithic") {
   Curve("IntCal20", "intcal20.14c");
    R_Date("LTL6186A", 7000, 60);
    R_Date("R-553", 9100, 100);
    R_Date("R-554", 9330, 100);
    R_Date("R-552", 9490, 199);
```

```
    R_Date("TO-6081", 8110, 80);
    R_Date("TO-3421", 9140, 70);
    R_Date("TO-3422", 9370, 80);
    R_Date("R-1525a", 7380, 90);
    R_Date("R-1527", 8590, 90);
    R_Date("R-1526", 8840, 120);
    R_Date("R-1528", 8780, 110);
    R_Date("R-1529a", 9220, 100);
    R_Date("R-1529", 9420, 90);
    R_Date("R-1525", 7460, 90);
    R_Date("F-90", 9370, 150);
    R_Date("LTL232A", 8823, 40);
    R_Date("LTL4231A", 8885, 65);
    R_Date("OxA-2630", 7480, 100);
    R_Date("R-400", 7330, 85);
    R_Date("R-395", 8080, 90);
    R_Date("R-396", 8450, 90);
    R_Date("R-398", 8780, 90);
    R_Date("R-397", 8890, 90);
    R_Date("R-399", 8990, 90);
    R_Date("R-341", 8565, 80);
    R_Date("AA-10951", 9458, 91);
  };
 Sigma_Boundary("Central_Italy_mesolithic_end");
 };
};

Plot(){
Sequence("Central_Italy_mesolithic"){
 Boundary("Central_Italy_mesolithic_start");
  Phase("Central_Italy_mesolithic") {
   Curve("IntCal20", "intcal20.14c");
    R_Date("LTL6186A", 7000, 60);
    R_Date("R-553", 9100, 100);
    R_Date("R-554", 9330, 100);
    R_Date("R-552", 9490, 199);
    R_Date("TO-6081", 8110, 80);
    R_Date("TO-3421", 9140, 70);
    R_Date("TO-3422", 9370, 80);
    R_Date("R-1525a", 7380, 90);
    R_Date("R-1527", 8590, 90);
    R_Date("R-1526", 8840, 120);
    R_Date("R-1528", 8780, 110);
    R_Date("R-1529a", 9220, 100);
    R_Date("R-1529", 9420, 90);
    R_Date("R-1525", 7460, 90);
    R_Date("F-90", 9370, 150);
    R_Date("LTL232A", 8823, 40);
    R_Date("LTL4231A", 8885, 65);
    R_Date("OxA-2630", 7480, 100);
```

```
    R_Date("R-400", 7330, 85);
    R_Date("R-395", 8080, 90);
    R_Date("R-396", 8450, 90);
    R_Date("R-398", 8780, 90);
    R_Date("R-397", 8890, 90);
    R_Date("R-399", 8990, 90);
    R_Date("R-341", 8565, 80);
    R_Date("AA-10951", 9458, 91);
  };
 Boundary("Central_Italy_mesolithic_end");
 };
};
```

Corsica:

```
Plot(){
Sequence("Corsica_mesolithic"){
 Sigma_Boundary("Corsica_mesolithic_start");
  Phase("Corsica_mesolithic") {
   Curve("IntCal20", "intcal20.14c");
    R_Date("GIF-2705", 8520, 150);
    R_Date("GIF-1851", 7700, 150);
    R_Date("UGAMS-3825", 7700, 39);
    R_Date("UGAMS-3824", 7920, 35);
    R_Date("Beta-318791", 8940, 40);
    R_Date("Poz-44201", 8970, 60);
    R_Date("GIF-796", 7300, 160);
    R_Date("GIF-1961", 7310, 170);
    R_Date("GIF-1962", 7600, 150);
    R_Date("GIF-1963", 8300, 150);
    R_Date("GIF-795", 8560, 170);
    R_Date("Ly 823", 8130, 70);
    R_Date("AA-35788", 7930, 80);
    R_Date("AA-18108", 8050, 60);
    R_Date("AA-35791", 8115, 60);
    R_Date("AA-35789", 8135, 65);
    R_Date("ETH-8305", 8225, 80);
    R_Date("AA-35790", 8275, 65);
    R_Date("AA-35792", 8315, 75);
    R_Date("AA-18110", 8335, 70);
    R_Date("AA-18112", 8415, 65);
    R_Date("AA-18109", 8965, 70);
    R_Date("Ly-2837", 9140, 300);
    R_Date("LGQ-507", 7840, 310);
  };
 Sigma_Boundary("Corsica_mesolithic_end");
 };
};

Plot(){
```

```
Sequence("Corsica_mesolithic"){
 Boundary("Corsica_mesolithic_start");
  Phase("Corsica_mesolithic") {
   Curve("IntCal20", "intcal20.14c");
    R_Date("GIF-2705", 8520, 150);
    R_Date("GIF-1851", 7700, 150);
    R_Date("UGAMS-3825", 7700, 39);
    R_Date("UGAMS-3824", 7920, 35);
    R_Date("Beta-318791", 8940, 40);
    R_Date("Poz-44201", 8970, 60);
    R_Date("GIF-796", 7300, 160);
    R_Date("GIF-1961", 7310, 170);
    R_Date("GIF-1962", 7600, 150);
    R_Date("GIF-1963", 8300, 150);
    R_Date("GIF-795", 8560, 170);
    R_Date("Ly 823", 8130, 70);
    R_Date("AA-35788", 7930, 80);
    R_Date("AA-18108", 8050, 60);
    R_Date("AA-35791", 8115, 60);
    R_Date("AA-35789", 8135, 65);
    R_Date("ETH-8305", 8225, 80);
    R_Date("AA-35790", 8275, 65);
    R_Date("AA-35792", 8315, 75);
    R_Date("AA-18110", 8335, 70);
    R_Date("AA-18112", 8415, 65);
    R_Date("AA-18109", 8965, 70);
    R_Date("Ly-2837", 9140, 300);
    R_Date("LGQ-507", 7840, 310);
  };
 Boundary("Corsica_mesolithic_end");
 };
};
```

Northern Italy:

```
Plot(){
Sequence("Northern_Italy_mesolithic"){
 Sigma_Boundary("Northern_Italy_mesolithic_start");
  Phase("Northern_Italy_mesolithic") {
   Curve("IntCal20", "intcal20.14c");
    R_Date("KIA30570", 6627, 33);
    R_Date("Bln-3276", 6610, 60);
    R_Date("R-109", 7800, 100);
    R_Date("R-148", 8100, 90);
    R_Date("R-126", 8400, 100);
    R_Date("Beta-76823", 7870, 60);
    R_Date("GrN-20886", 6220, 45);
    R_Date("GX-18843", 8820, 112);
    R_Date("R-895", 9370, 130);
    R_Date("BLN-3277", 6810, 70);
```

```
R_Date("R-1497", 7000, 200);
R_Date("R-2567", 7112, 121);
R_Date("R-1043", 7050, 60);
R_Date("R-1042", 7230, 240);
R_Date("R-1044", 7620, 150);
R_Date("R-1045", 8380, 60);
R_Date("R-1045a", 8650, 70);
R_Date("R-819", 8650, 70);
R_Date("Gd-6154", 6000, 140);
R_Date("Gd-5639", 6460, 70);
R_Date("Gd-6153", 6500, 150);
R_Date("Gd-6155", 7280, 150);
R_Date("Gd-5613", 7660, 100);
R_Date("Gd-54797", 7870, 70);
R_Date("Gd-5481", 8140, 80);
R_Date("Gd-5492", 8270, 90);
R_Date("Gd-4510", 8520, 190);
R_Date("Gd-5618", 9110, 70);
R_Date("Gd-6132", 9300, 100);
R_Date("R-1041", 8260, 60);
R_Date("R-982", 6930, 60);
R_Date("GrN-26795", 6410, 50);
Curve("Marine20", "marine20.14c");
Delta_R("GrN-25474", 131, 30);
R_Date("GrN-25474", 6480, 40);
Curve("IntCal20", "intcal20.14c");
R_Date("GrN-27229", 6510, 70);
R_Date("GrA-19912", 6620, 60);
R_Date("GrA-14106", 8045, 40);
R_Date("GrN-25137", 8060, 70);
R_Date("GrN-25138", 8110, 90);
R_Date("GrA-11818", 8250, 50);
R_Date("GrN-25139", 8350, 120);
R_Date("GrN-25800", 8160, 100);
R_Date("HAR-8871", 6790, 120);
R_Date("GrN-18091", 6870, 70);
R_Date("Beta-35241", 7850, 80);
R_Date("Grn-21889", 7870, 50);
R_Date("GD-6156", 7500, 150);
R_Date("GD-6160", 8220, 110);
R_Date("KL-3636.01", 8540, 180);
R_Date("KL-3630.02", 9020, 190);
R_Date("Rome-394", 6620, 80);
R_Date("R-394", 6624, 45);
R_Date("GrN-19590", 8880, 150);
R_Date("ETH-15980", 6005, 75);
R_Date("ETH-15981", 6170, 75);
R_Date("UtC-202", 6210, 60);
R_Date("Utc-7202", 6210, 60);
R_Date("UtC-7201", 6380, 50);
```

```
R_Date("ETH-15984", 6410, 75);
R_Date("OZE033", 7091, 45);
R_Date("OxA-7468", 7425, 55);
R_Date("R-1939", 7440, 50);
R_Date("GX-21793", 8260, 175);
R_Date("R-1937", 8380, 70);
R_Date("I-12520", 7630, 120);
R_Date("I-12687", 7670, 120);
R_Date("Bln-2839", 8260, 60);
R_Date("R-3130", 6414, 203);
R_Date("R-3129", 7633, 85);
R_Date("R-3122", 8585, 75);
R_Date("R-3123", 8800, 63);
R_Date("Birm-830", 6960, 130);
R_Date("NA-192", 7540, 210);
R_Date("NA-159", 7950, 240);
R_Date("NA-209", 8530, 300);
R_Date("NA-139", 8830, 260);
R_Date("NA-210", 8950, 170);
R_Date("NA-211", 9080, 350);
R_Date("NA-231", 9130, 150);
R_Date("NA-212", 9200, 150);
R_Date("NA-251", 7030, 750);
R_Date("R-2714", 8688, 99);
R_Date("R-2566", 9377, 198);
R_Date("R-1148", 6870, 50);
R_Date("R-1149", 8200, 50);
R_Date("R-1150", 8240, 200);
R_Date("R-1151", 9320, 50);
R_Date("R-1137A", 7500, 160);
R_Date("R-1137B", 7800, 80);
R_Date("R-1137", 7850, 60);
R_Date("R-1138", 8140, 80);
R_Date("R-1139", 8220, 80);
R_Date("R-1140", 8560, 70);
R_Date("R-1141", 8590, 90);
R_Date("R-1142", 8740, 90);
R_Date("R-1143", 9090, 90);
R_Date("R-1144", 9100, 90);
R_Date("R-1145", 9200, 60);
R_Date("R-1146", 9420, 60);
R_Date("R-1146B", 9490, 80);
R_Date("R-487", 7250, 110);
R_Date("R-488", 7540, 75);
R_Date("R-488a", 7585, 75);
R_Date("R-491a", 7740, 150);
R_Date("R-489", 7810, 75);
R_Date("R-489a", 7810, 95);
R_Date("R-490", 7860, 110);
R_Date("R-490a", 7860, 100);
```

```
   R_Date("R-491", 8000, 110);
   R_Date("KIA-10363", 6968, 41);
   R_Date("KIA-10362", 7283, 38);
   R_Date("KIA-10366", 7725, 49);
   R_Date("KIA-10367", 7902, 55);
   R_Date("KIA-10364", 7971, 42);
   R_Date("KIA-10368", 8193, 66);
   R_Date("KIA-10365", 8323, 63);
   R_Date("KIA-10369", 8509, 44);
   R_Date("KIA-10370", 8847, 57);
   R_Date("Grn-20093", 9410, 80);
  };
 Sigma_Boundary("Northern_Italy_mesolithic_end");
 };
};

Plot(){
Sequence("Northern_Italy_mesolithic"){
 Boundary("Northern_Italy_mesolithic_start");
  Phase("Northern_Italy_mesolithic") {
   Curve("IntCal20", "intcal20.14c");
   R_Date("KIA30570", 6627, 33);
   R_Date("Bln-3276", 6610, 60);
   R_Date("R-109", 7800, 100);
   R_Date("R-148", 8100, 90);
   R_Date("R-126", 8400, 100);
   R_Date("Beta-76823", 7870, 60);
   R_Date("GrN-20886", 6220, 45);
   R_Date("GX-18843", 8820, 112);
   R_Date("R-895", 9370, 130);
   R_Date("BLN-3277", 6810, 70);
   R_Date("R-1497", 7000, 200);
   R_Date("R-2567", 7112, 121);
   R_Date("R-1043", 7050, 60);
   R_Date("R-1042", 7230, 240);
   R_Date("R-1044", 7620, 150);
   R_Date("R-1045", 8380, 60);
   R_Date("R-1045a", 8650, 70);
   R_Date("R-819", 8650, 70);
   R_Date("Gd-6154", 6000, 140);
   R_Date("Gd-5639", 6460, 70);
   R_Date("Gd-6153", 6500, 150);
   R_Date("Gd-6155", 7280, 150);
   R_Date("Gd-5613", 7660, 100);
   R_Date("Gd-54797", 7870, 70);
   R_Date("Gd-5481", 8140, 80);
   R_Date("Gd-5492", 8270, 90);
   R_Date("Gd-4510", 8520, 190);
   R_Date("Gd-5618", 9110, 70);
   R_Date("Gd-6132", 9300, 100);
```

```
R_Date("R-1041", 8260, 60);
R_Date("R-982", 6930, 60);
R_Date("GrN-26795", 6410, 50);
Curve("Marine20", "marine20.14c");
Delta_R("GrN-25474", 131, 30);
R_Date("GrN-25474", 6480, 40);
Curve("IntCal20", "intcal20.14c");
R_Date("GrN-27229", 6510, 70);
R_Date("GrA-19912", 6620, 60);
R_Date("GrA-14106", 8045, 40);
R_Date("GrN-25137", 8060, 70);
R_Date("GrN-25138", 8110, 90);
R_Date("GrA-11818", 8250, 50);
R_Date("GrN-25139", 8350, 120);
R_Date("GrN-25800", 8160, 100);
R_Date("HAR-8871", 6790, 120);
R_Date("GrN-18091", 6870, 70);
R_Date("Beta-35241", 7850, 80);
R_Date("Grn-21889", 7870, 50);
R_Date("GD-6156", 7500, 150);
R_Date("GD-6160", 8220, 110);
R_Date("KL-3636.01", 8540, 180);
R_Date("KL-3630.02", 9020, 190);
R_Date("Rome-394", 6620, 80);
R_Date("R-394", 6624, 45);
R_Date("GrN-19590", 8880, 150);
R_Date("ETH-15980", 6005, 75);
R_Date("ETH-15981", 6170, 75);
R_Date("UtC-202", 6210, 60);
R_Date("Utc-7202", 6210, 60);
R_Date("UtC-7201", 6380, 50);
R_Date("ETH-15984", 6410, 75);
R_Date("OZE033", 7091, 45);
R_Date("OxA-7468", 7425, 55);
R_Date("R-1939", 7440, 50);
R_Date("GX-21793", 8260, 175);
R_Date("R-1937", 8380, 70);
R_Date("I-12520", 7630, 120);
R_Date("I-12687", 7670, 120);
R_Date("Bln-2839", 8260, 60);
R_Date("R-3130", 6414, 203);
R_Date("R-3129", 7633, 85);
R_Date("R-3122", 8585, 75);
R_Date("R-3123", 8800, 63);
R_Date("Birm-830", 6960, 130);
R_Date("NA-192", 7540, 210);
R_Date("NA-159", 7950, 240);
R_Date("NA-209", 8530, 300);
R_Date("NA-139", 8830, 260);
R_Date("NA-210", 8950, 170);
```

```
  R_Date("NA-211", 9080, 350);
  R_Date("NA-231", 9130, 150);
  R_Date("NA-212", 9200, 150);
  R_Date("NA-251", 7030, 750);
  R_Date("R-2714", 8688, 99);
  R_Date("R-2566", 9377, 198);
  R_Date("R-1148", 6870, 50);
  R_Date("R-1149", 8200, 50);
  R_Date("R-1150", 8240, 200);
  R_Date("R-1151", 9320, 50);
  R_Date("R-1137A", 7500, 160);
  R_Date("R-1137B", 7800, 80);
  R_Date("R-1137", 7850, 60);
  R_Date("R-1138", 8140, 80);
  R_Date("R-1139", 8220, 80);
  R_Date("R-1140", 8560, 70);
  R_Date("R-1141", 8590, 90);
  R_Date("R-1142", 8740, 90);
  R_Date("R-1143", 9090, 90);
  R_Date("R-1144", 9100, 90);
  R_Date("R-1145", 9200, 60);
  R_Date("R-1146", 9420, 60);
  R_Date("R-1146B", 9490, 80);
  R_Date("R-487", 7250, 110);
  R_Date("R-488", 7540, 75);
  R_Date("R-488a", 7585, 75);
  R_Date("R-491a", 7740, 150);
  R_Date("R-489", 7810, 75);
  R_Date("R-489a", 7810, 95);
  R_Date("R-490", 7860, 110);
  R_Date("R-490a", 7860, 100);
  R_Date("R-491", 8000, 110);
  R_Date("KIA-10363", 6968, 41);
  R_Date("KIA-10362", 7283, 38);
  R_Date("KIA-10366", 7725, 49);
  R_Date("KIA-10367", 7902, 55);
  R_Date("KIA-10364", 7971, 42);
  R_Date("KIA-10368", 8193, 66);
  R_Date("KIA-10365", 8323, 63);
  R_Date("KIA-10369", 8509, 44);
  R_Date("KIA-10370", 8847, 57);
  R_Date("Grn-20093", 9410, 80);
 };
 Boundary("Northern_Italy_mesolithic_end");
 };
};
```

Sardinia:

```
Plot(){
```

```
Sequence("Sardinia_mesolithic"){
 Sigma_Boundary("Sardinia_mesolithic_start");
  Phase("Sardinia_mesolithic") {
   Curve("IntCal20", "intcal20.14c");
    R_Date("BM-2139R", 7760, 130);
    R_Date("UtC-301", 7860, 60);
    R_Date("UtC-22", 8040, 180);
    R_Date("UtC-235", 8160, 130);
    R_Date("UtC-300", 8750, 140);
    R_Date("UtC-726", 8960, 110);
    R_Date("GrN-11434", 9120, 380);
    R_Date("Beta-167932", 7400, 40);
    R_Date("Beta-145956", 7740, 50);
    R_Date("Beta-145957", 7920, 50);
    R_Date("AA-79862", 7127, 59);
    R_Date("AA-76545", 7678, 73);
    R_Date("AA-76546", 7860, 44);
    R_Date("Beta-334464", 8080, 40);
    R_Date("Beta-334465", 8190, 40);
    R_Date("Beta-334467", 8350, 40);
    R_Date("Beta-334466", 8410, 50);
    R_Date("AA-80544", 8780, 130);
    R_Date("LTL1588A", 8358, 40);
    R_Date("LTL1587A", 8512, 70);
  };
 Sigma_Boundary("Sardinia_mesolithic_end");
 };
};

Plot(){
Sequence("Sardinia_mesolithic"){
 Boundary("Sardinia_mesolithic_start");
  Phase("Sardinia_mesolithic") {
   Curve("IntCal20", "intcal20.14c");
    R_Date("BM-2139R", 7760, 130);
    R_Date("UtC-301", 7860, 60);
    R_Date("UtC-22", 8040, 180);
    R_Date("UtC-235", 8160, 130);
    R_Date("UtC-300", 8750, 140);
    R_Date("UtC-726", 8960, 110);
    R_Date("GrN-11434", 9120, 380);
    R_Date("Beta-167932", 7400, 40);
    R_Date("Beta-145956", 7740, 50);
    R_Date("Beta-145957", 7920, 50);
    R_Date("AA-79862", 7127, 59);
    R_Date("AA-76545", 7678, 73);
    R_Date("AA-76546", 7860, 44);
    R_Date("Beta-334464", 8080, 40);
    R_Date("Beta-334465", 8190, 40);
    R_Date("Beta-334467", 8350, 40);
```

```
   R_Date("Beta-334466", 8410, 50);
   R_Date("AA-80544", 8780, 130);
   R_Date("LTL1588A", 8358, 40);
   R_Date("LTL1587A", 8512, 70);
  };
 Boundary("Sardinia_mesolithic_end");
 };
};
```

Sicily:

```
Plot(){
Sequence("Sicily_mesolithic"){
 Sigma_Boundary("Sicily_mesolithic_start");
  Phase("Sicily_mesolithic") {
   Curve("IntCal20", "intcal20.14c");
    Curve("Marine20", "marine20.14c");
    Delta_R("OxA-14256", 88, 50);
    R_Date("OxA-14256", 8159, 37);
    Curve("IntCal20", "intcal20.14c");
    R_Date("LTL874A", 8608, 65);
    R_Date("LTL876A", 8619, 65);
    R_Date("LTL875A", 8699, 60);
    Curve("Marine20", "marine20.14c");
    Delta_R("?", 88, 50);
    R_Date("?", 8960, 130);
    Curve("IntCal20", "intcal20.14c");
    R_Date("MAMS-40712", 7036, 25);
    R_Date("MAMS-48212", 7051, 27);
    Curve("Marine20", "marine20.14c");
    Delta_R("KIA-36032", 88, 50);
    R_Date("KIA-36032", 7175, 45);
    Curve("IntCal20", "intcal20.14c");
    R_Date("MAMS-40726", 7471, 26);
    R_Date("MAMS-40709", 7713, 26);
    Curve("Marine20", "marine20.14c");
    Delta_R("KIA-36034", 88, 50);
    R_Date("KIA-36034", 7730, 80);
    Delta_R("OxA-13662", 88, 50);
    R_Date("OxA-13662", 7744, 33);
    Curve("IntCal20", "intcal20.14c");
    R_Date("MAMS-40721", 7807, 26);
    R_Date("MAMS-40719", 7809, 26);
    R_Date("MAMS-40720", 7809, 26);
    R_Date("MAMS-40722", 7809, 26);
    R_Date("MAMS-40711", 7848, 26);
    R_Date("OxA-V-2364-41", 7871, 30);
    R_Date("P-2734", 7910, 70);
    Curve("Marine20", "marine20.14c");
    Delta_R("MAMS-16238", 88, 50);
```

```
   R_Date("MAMS-16238", 7959, 25);
   Delta_R("MAMS-16239", 88, 50);
   R_Date("MAMS-16239", 8083, 26);
   Curve("IntCal20", "intcal20.14c");
   R_Date("P-2735", 8330, 80);
   Curve("Marine20", "marine20.14c");
   Delta_R("OxA-13419", 88, 50);
   R_Date("OxA-13419", 8745, 55);
   Curve("IntCal20", "intcal20.14c");
   R_Date("P-2556", 9030, 100);
   R_Date("P-2557", 9180, 100);
   R_Date("P-2558", 9300, 100);
   R_Date("MAMS-40708", 9436, 36);
   R_Date("MAMS-40710", 9436, 29);
   R_Date("LTL1514A", 7998, 80);
   R_Date("LTL1515A", 8320, 85);
   Curve("Marine20", "marine20.14c");
   Delta_R("OxA-14257", 88, 50);
   R_Date("OxA-14257", 8166, 37);
   Curve("IntCal20", "intcal20.14c");
   R_Date("LTL772A", 8467, 67);
   R_Date("LTL771A", 9332, 60);
   R_Date("OxA-534", 8600, 100);
   Curve("Marine20", "marine20.14c");
   Delta_R("OxA-18070", 88, 50);
   R_Date("OxA-18070", 8620, 45);
   Delta_R("OxA-18069", 88, 50);
   R_Date("OxA-18069", 8685, 40);
   Delta_R("OxA-18071", 88, 50);
   R_Date("OxA-18071", 8785, 45);
   Curve("IntCal20", "intcal20.14c");
   R_Date("UtC-1355", 8460, 70);
   R_Date("UtC-1424", 8700, 150);
  };
 Sigma_Boundary("Sicily_mesolithic_end");
 };
};

Plot(){
Sequence("Sicily_mesolithic"){
 Boundary("Sicily_mesolithic_start");
  Phase("Sicily_mesolithic") {
   Curve("IntCal20", "intcal20.14c");
   Curve("Marine20", "marine20.14c");
   Delta_R("OxA-14256", 88, 50);
   R_Date("OxA-14256", 8159, 37);
   Curve("IntCal20", "intcal20.14c");
   R_Date("LTL874A", 8608, 65);
   R_Date("LTL876A", 8619, 65);
   R_Date("LTL875A", 8699, 60);
```

```
Curve("Marine20", "marine20.14c");
Delta_R("?", 88, 50);
R_Date("?", 8960, 130);
Curve("IntCal20", "intcal20.14c");
R_Date("MAMS-40712", 7036, 25);
R_Date("MAMS-48212", 7051, 27);
Curve("Marine20", "marine20.14c");
Delta_R("KIA-36032", 88, 50);
R_Date("KIA-36032", 7175, 45);
Curve("IntCal20", "intcal20.14c");
R_Date("MAMS-40726", 7471, 26);
R_Date("MAMS-40709", 7713, 26);
Curve("Marine20", "marine20.14c");
Delta_R("KIA-36034", 88, 50);
R_Date("KIA-36034", 7730, 80);
Delta_R("OxA-13662", 88, 50);
R_Date("OxA-13662", 7744, 33);
Curve("IntCal20", "intcal20.14c");
R_Date("MAMS-40721", 7807, 26);
R_Date("MAMS-40719", 7809, 26);
R_Date("MAMS-40720", 7809, 26);
R_Date("MAMS-40722", 7809, 26);
R_Date("MAMS-40711", 7848, 26);
R_Date("OxA-V-2364-41", 7871, 30);
R_Date("P-2734", 7910, 70);
Curve("Marine20", "marine20.14c");
Delta_R("MAMS-16238", 88, 50);
R_Date("MAMS-16238", 7959, 25);
Delta_R("MAMS-16239", 88, 50);
R_Date("MAMS-16239", 8083, 26);
Curve("IntCal20", "intcal20.14c");
R_Date("P-2735", 8330, 80);
Curve("Marine20", "marine20.14c");
Delta_R("OxA-13419", 88, 50);
R_Date("OxA-13419", 8745, 55);
Curve("IntCal20", "intcal20.14c");
R_Date("P-2556", 9030, 100);
R_Date("P-2557", 9180, 100);
R_Date("P-2558", 9300, 100);
R_Date("MAMS-40708", 9436, 36);
R_Date("MAMS-40710", 9436, 29);
R_Date("LTL1514A", 7998, 80);
R_Date("LTL1515A", 8320, 85);
Curve("Marine20", "marine20.14c");
Delta_R("OxA-14257", 88, 50);
R_Date("OxA-14257", 8166, 37);
Curve("IntCal20", "intcal20.14c");
R_Date("LTL772A", 8467, 67);
R_Date("LTL771A", 9332, 60);
R_Date("OxA-534", 8600, 100);
```

```
   Curve("Marine20", "marine20.14c");
   Delta_R("OxA-18070", 88, 50);
   R_Date("OxA-18070", 8620, 45);
   Delta_R("OxA-18069", 88, 50);
   R_Date("OxA-18069", 8685, 40);
   Delta_R("OxA-18071", 88, 50);
   R_Date("OxA-18071", 8785, 45);
   Curve("IntCal20", "intcal20.14c");
   R_Date("UtC-1355", 8460, 70);
   R_Date("UtC-1424", 8700, 150);
  };
 Boundary("Sicily_mesolithic_end");
 };
};
```

Siciliy (with alternate phase labels for some dates)

```
Plot(){
Sequence("SicilyAlt_mesolithic"){
 Sigma_Boundary("SicilyAlt_mesolithic_start");
  Phase("SicilyAlt_mesolithic") {
  Curve("IntCal20", "intcal20.14c");
   Curve("Marine20", "marine20.14c");
   Delta_R("OxA-14256", 88, 50);
   R_Date("OxA-14256", 8159, 37);
   Curve("IntCal20", "intcal20.14c");
   R_Date("LTL874A", 8608, 65);
   R_Date("LTL876A", 8619, 65);
   R_Date("LTL875A", 8699, 60);
   Curve("Marine20", "marine20.14c");
   Delta_R("?", 88, 50);
   R_Date("?", 8960, 130);
   Curve("IntCal20", "intcal20.14c");
   R_Date("MAMS-40726", 7471, 26);
   R_Date("MAMS-40709", 7713, 26);
   Curve("Marine20", "marine20.14c");
   Delta_R("KIA-36034", 88, 50);
   R_Date("KIA-36034", 7730, 80);
   Delta_R("OxA-13662", 88, 50);
   R_Date("OxA-13662", 7744, 33);
   Curve("IntCal20", "intcal20.14c");
   R_Date("MAMS-40721", 7807, 26);
   R_Date("MAMS-40719", 7809, 26);
   R_Date("MAMS-40720", 7809, 26);
   R_Date("MAMS-40722", 7809, 26);
   R_Date("MAMS-40711", 7848, 26);
   R_Date("OxA-V-2364-41", 7871, 30);
   R_Date("P-2734", 7910, 70);
   Curve("Marine20", "marine20.14c");
   Delta_R("MAMS-16238", 88, 50);
```

```
    R_Date("MAMS-16238", 7959, 25);
    Delta_R("MAMS-16239", 88, 50);
    R_Date("MAMS-16239", 8083, 26);
    Curve("IntCal20", "intcal20.14c");
    R_Date("P-2735", 8330, 80);
    Curve("Marine20", "marine20.14c");
    Delta_R("OxA-13419", 88, 50);
    R_Date("OxA-13419", 8745, 55);
    Curve("IntCal20", "intcal20.14c");
    R_Date("P-2556", 9030, 100);
    R_Date("P-2557", 9180, 100);
    R_Date("P-2558", 9300, 100);
    R_Date("MAMS-40708", 9436, 36);
    R_Date("MAMS-40710", 9436, 29);
    R_Date("LTL1514A", 7998, 80);
    R_Date("LTL1515A", 8320, 85);
    Curve("Marine20", "marine20.14c");
    Delta_R("OxA-14257", 88, 50);
    R_Date("OxA-14257", 8166, 37);
    Curve("IntCal20", "intcal20.14c");
    R_Date("LTL772A", 8467, 67);
    R_Date("LTL771A", 9332, 60);
    R_Date("OxA-534", 8600, 100);
    Curve("Marine20", "marine20.14c");
    Delta_R("OxA-18070", 88, 50);
    R_Date("OxA-18070", 8620, 45);
    Delta_R("OxA-18069", 88, 50);
    R_Date("OxA-18069", 8685, 40);
    Delta_R("OxA-18071", 88, 50);
    R_Date("OxA-18071", 8785, 45);
    Curve("IntCal20", "intcal20.14c");
    R_Date("UtC-1355", 8460, 70);
    R_Date("UtC-1424", 8700, 150);
  };
 Sigma_Boundary("SicilyAlt_mesolithic_end");
 };
};

Plot(){
Sequence("SicilyAlt_mesolithic"){
 Boundary("SicilyAlt_mesolithic_start");
  Phase("SicilyAlt_mesolithic") {
   Curve("IntCal20", "intcal20.14c");
    Curve("Marine20", "marine20.14c");
    Delta_R("OxA-14256", 88, 50);
    R_Date("OxA-14256", 8159, 37);
    Curve("IntCal20", "intcal20.14c");
    R_Date("LTL874A", 8608, 65);
    R_Date("LTL876A", 8619, 65);
    R_Date("LTL875A", 8699, 60);
```

```
Curve("Marine20", "marine20.14c");
Delta_R("?", 88, 50);
R_Date("?", 8960, 130);
Curve("IntCal20", "intcal20.14c");
R_Date("MAMS-40726", 7471, 26);
R_Date("MAMS-40709", 7713, 26);
Curve("Marine20", "marine20.14c");
Delta_R("KIA-36034", 88, 50);
R_Date("KIA-36034", 7730, 80);
Delta_R("OxA-13662", 88, 50);
R_Date("OxA-13662", 7744, 33);
Curve("IntCal20", "intcal20.14c");
R_Date("MAMS-40721", 7807, 26);
R_Date("MAMS-40719", 7809, 26);
R_Date("MAMS-40720", 7809, 26);
R_Date("MAMS-40722", 7809, 26);
R_Date("MAMS-40711", 7848, 26);
R_Date("OxA-V-2364-41", 7871, 30);
R_Date("P-2734", 7910, 70);
Curve("Marine20", "marine20.14c");
Delta_R("MAMS-16238", 88, 50);
R_Date("MAMS-16238", 7959, 25);
Delta_R("MAMS-16239", 88, 50);
R_Date("MAMS-16239", 8083, 26);
Curve("IntCal20", "intcal20.14c");
R_Date("P-2735", 8330, 80);
Curve("Marine20", "marine20.14c");
Delta_R("OxA-13419", 88, 50);
R_Date("OxA-13419", 8745, 55);
Curve("IntCal20", "intcal20.14c");
R_Date("P-2556", 9030, 100);
R_Date("P-2557", 9180, 100);
R_Date("P-2558", 9300, 100);
R_Date("MAMS-40708", 9436, 36);
R_Date("MAMS-40710", 9436, 29);
R_Date("LTL1514A", 7998, 80);
R_Date("LTL1515A", 8320, 85);
Curve("Marine20", "marine20.14c");
Delta_R("OxA-14257", 88, 50);
R_Date("OxA-14257", 8166, 37);
Curve("IntCal20", "intcal20.14c");
R_Date("LTL772A", 8467, 67);
R_Date("LTL771A", 9332, 60);
R_Date("OxA-534", 8600, 100);
Curve("Marine20", "marine20.14c");
Delta_R("OxA-18070", 88, 50);
R_Date("OxA-18070", 8620, 45);
Delta_R("OxA-18069", 88, 50);
R_Date("OxA-18069", 8685, 40);
Delta_R("OxA-18071", 88, 50);
```

```
   R_Date("OxA-18071", 8785, 45);
   Curve("IntCal20", "intcal20.14c");
   R_Date("UtC-1355", 8460, 70);
   R_Date("UtC-1424", 8700, 150);
  };
 Boundary("SicilyAlt_mesolithic_end");
 };
};
```

Southern Italy

```
Plot(){
Sequence("Southern_Italy_mesolithic"){
 Sigma_Boundary("Southern_Italy_mesolithic_start");
  Phase("Southern_Italy_mesolithic") {
   Curve("IntCal20", "intcal20.14c");
    R_Date("LTL13713A", 7463, 40);
    R_Date("LTL13714A", 8135, 45);
    R_Date("R-187", 8735, 80);
    R_Date("LTL13712A", 8782, 45);
    R_Date("R-187a", 8875, 85);
    R_Date("LTL13715A", 8878, 45);
    R_Date("LTL13716A", 8991, 45);
    R_Date("LTL13717A", 9076, 45);
    R_Date("UtC-780", 8240, 120);
    R_Date("UtC-1417", 8290, 50);
    R_Date("F-33", 7540, 135);
    R_Date("F-34", 9320, 180);
    R_Date("F-36", 9530, 170);
    Curve("Marine20", "marine20.14c");
    Delta_R("Pi-10", 131, 30);
    R_Date("Pi-10", 8619, 200);
    Curve("IntCal20", "intcal20.14c");
    R_Date("R-54", 9050, 100);
    R_Date("R-452", 6970, 90);
    R_Date("R-450", 7045, 90);
    R_Date("R-451", 7160, 90);
    R_Date("R-447", 7400, 90);
    R_Date("R-445", 7420, 90);
    R_Date("R-448", 7570, 90);
    R_Date("R-446", 7620, 90);
    R_Date("R-449", 7800, 90);
    R_Date("R-453", 8024, 100);
    R_Date("R-286", 9020, 125);
    R_Date("R-287", 9035, 100);
    R_Date("R-188", 9070, 80);
    R_Date("LTL3578A", 8963, 60);
  };
 Sigma_Boundary("Southern_Italy_mesolithic_end");
 };
```

```
};

Plot(){
Sequence("Southern_Italy_mesolithic"){
 Boundary("Southern_Italy_mesolithic_start");
  Phase("Southern_Italy_mesolithic") {
   Curve("IntCal20", "intcal20.14c");
    R_Date("LTL13713A", 7463, 40);
    R_Date("LTL13714A", 8135, 45);
    R_Date("R-187", 8735, 80);
    R_Date("LTL13712A", 8782, 45);
    R_Date("R-187a", 8875, 85);
    R_Date("LTL13715A", 8878, 45);
    R_Date("LTL13716A", 8991, 45);
    R_Date("LTL13717A", 9076, 45);
    R_Date("UtC-780", 8240, 120);
    R_Date("UtC-1417", 8290, 50);
    R_Date("F-33", 7540, 135);
    R_Date("F-34", 9320, 180);
    R_Date("F-36", 9530, 170);
    Curve("Marine20", "marine20.14c");
    Delta_R("Pi-10", 131, 30);
    R_Date("Pi-10", 8619, 200);
    Curve("IntCal20", "intcal20.14c");
    R_Date("R-54", 9050, 100);
    R_Date("R-452", 6970, 90);
    R_Date("R-450", 7045, 90);
    R_Date("R-451", 7160, 90);
    R_Date("R-447", 7400, 90);
    R_Date("R-445", 7420, 90);
    R_Date("R-448", 7570, 90);
    R_Date("R-446", 7620, 90);
    R_Date("R-449", 7800, 90);
    R_Date("R-453", 8024, 100);
    R_Date("R-286", 9020, 125);
    R_Date("R-287", 9035, 100);
    R_Date("R-188", 9070, 80);
    R_Date("LTL3578A", 8963, 60);
  };
 Boundary("Southern_Italy_mesolithic_end");
 };
};
```

Neolithic

Central Italy

```
Plot(){
Sequence("Central_Italy_neolithic"){
 Sigma_Boundary("Central_Italy_neolithic_start");
```

```
Phase("Central_Italy_neolithic") {
 Curve("IntCal20", "intcal20.14c");
  R_Date("GrA-13891", 6040, 50);
  R_Date("Beta-98081", 6270, 70);
  R_Date("Beta-95263", 6810, 130);
  R_Date("R-1102", 6000, 70);
  R_Date("R-1778", 6040, 70);
  R_Date("R-1101a", 6080, 70);
  R_Date("R-997", 6120, 70);
  R_Date("R-1420a", 6125, 70);
  R_Date("Beta-167164", 6130, 40);
  R_Date("Grn-24487", 6180, 120);
  R_Date("Beta-158321", 6290, 40);
  R_Date("Beta-149260", 6330, 40);
  R_Date("R-996a", 6330, 70);
  R_Date("Beta-149259", 6350, 70);
  R_Date("R-1146", 6410, 70);
  R_Date("R-1147", 6480, 70);
  R_Date("R-1148", 6585, 80);
  R_Date("?", 6375, 35);
  R_Date("?", 6410, 35);
  R_Date("?", 6460, 35);
  R_Date("LTL-5000A", 6149, 45);
  R_Date("LTL-5001A", 6158, 55);
  R_Date("R-468", 6575, 80);
  R_Date("LTL-57a", 6579, 60);
  R_Date("LTL-525a", 6651, 60);
  R_Date("LTL-061a", 6688, 110);
  R_Date("LTL-59a", 6718, 40);
  R_Date("LTL-58a", 6727, 75);
  R_Date("LTL-15953A", 6770, 45);
  R_Date("LTL-15952A", 6809, 45);
  R_Date("LTL-526a", 6823, 55);
  R_Date("LTL-60a", 6843, 40);
  R_Date("LTL15327a", 6225, 45);
  R_Date("LTL15328a", 6311, 45);
  R_Date("OxA-1958", 6540, 80);
  R_Date("?", 6372, 90);
  R_Date("Gif-4375", 6450, 90);
  R_Date("R-1410", 6170, 75);
  R_Date("R-2938", 6570, 63);
  R_Date("R-1411", 6590, 75);
  R_Date("PI-46", 6247, 130);
  R_Date("R-676", 6080, 50);
  R_Date("R-1167", 6860, 60);
  R_Date("LTL-6123A", 6000, 45);
  R_Date("?", 6152, 460);
  R_Date("Lyon-5202", 6275, 45);
  R_Date("LYON-3504", 6405, 35);
  R_Date("LTL-6124A", 6505, 50);
```

```
R_Date("Rome-2978", 6189, 43);
R_Date("R-2310", 6310, 75);
R_Date("R-2309", 6350, 75);
R_Date("R-2357", 6350, 60);
R_Date("R-2311", 6370, 95);
R_Date("Rome-3563", 6414, 40);
R_Date("Rome-2979", 6437, 60);
R_Date("Rome-3338", 6487, 64);
R_Date("Rome-3327", 6494, 49);
R_Date("Rome-2569", 6498, 57);
R_Date("R-2345", 6530, 65);
R_Date("R-2336", 6550, 70);
R_Date("R-", 6565, 64);
R_Date("R-2339", 6590, 65);
R_Date("Rome-3573", 6594, 61);
R_Date("R-2353", 6600, 45);
R_Date("Rome-3331", 6622, 64);
R_Date("Rome-3557", 6627, 34);
R_Date("Rome-3558", 6852, 28);
R_Date("R-2360", 6855, 65);
R_Date("Rome-3572", 6874, 37);
R_Date("LTL-16673A", 6492, 65);
R_Date("LTL-16671A", 6637, 45);
R_Date("LTL-16672A", 6769, 45);
R_Date("Ua-21097", 6440, 50);
R_Date("R-643a", 6580, 75);
R_Date("LTL078A", 6637, 83);
R_Date("LTL-079A2", 6638, 59);
R_Date("BM-2252R", 6000, 170);
R_Date("BM-2251", 6250, 90);
R_Date("BM-2250", 6290, 60);
R_Date("BM-2251R", 6570, 140);
R_Date("BM-2250R", 6590, 130);
R_Date("Beta-44155", 6100, 80);
R_Date("Beta-44114", 6180, 80);
R_Date("LTL-2656A", 6168, 50);
R_Date("LTL-2653A", 6624, 45);
R_Date("LTL15330a", 6263, 45);
R_Date("LTL15329a", 6397, 45);
R_Date("R-427", 6210, 60);
R_Date("X-45", 6580, 50);
R_Date("R-2702", 6447, 56);
R_Date("R-548", 6680, 80);
R_Date("LTL18596A", 6076, 45);
R_Date("LTL15989A", 6378, 45);
R_Date("LTL5191a", 6418, 50);
R_Date("?", 6500, 50);
R_Date("LTL15991A", 6500, 45);
R_Date("LTL5192A", 6500, 50);
R_Date("LTL12777A", 6555, 45);
```

```
    R_Date("LTL 15357A", 6647, 45);
    R_Date("LTL15990a", 6673, 45);
    R_Date("LTL 15323A", 6734, 45);
    R_Date("LTL15358a", 6916, 45);
    R_Date("AECV-2011c", 6790, 70);
    R_Date("AECV-2012c", 6860, 60);
    R_Date("R-598a", 6140, 70);
    R_Date("R-598", 6210, 75);
    R_Date("R-599", 6260, 85);
    R_Date("R-599a", 6260, 85);
    R_Date("OxA-1852", 6245, 90);
    R_Date("OxA-1851", 6270, 70);
    R_Date("OxA-1853", 6430, 80);
    R_Date("OxA-1854", 6120, 90);
    R_Date("LTL2407A", 6116, 55);
    R_Date("LTL2910A", 6486, 65);
    R_Date("LTL2404A", 6593, 45);
    R_Date("Pi-101", 6578, 135);
   };
  Sigma_Boundary("Central_Italy_neolithic_end");
 };
};

Plot(){
Sequence("Central_Italy_neolithic"){
 Boundary("Central_Italy_neolithic_start");
  Phase("Central_Italy_neolithic") {
   Curve("IntCal20", "intcal20.14c");
    R_Date("GrA-13891", 6040, 50);
    R_Date("Beta-98081", 6270, 70);
    R_Date("Beta-95263", 6810, 130);
    R_Date("R-1102", 6000, 70);
    R_Date("R-1778", 6040, 70);
    R_Date("R-1101a", 6080, 70);
    R_Date("R-997", 6120, 70);
    R_Date("R-1420a", 6125, 70);
    R_Date("Beta-167164", 6130, 40);
    R_Date("Grn-24487", 6180, 120);
    R_Date("Beta-158321", 6290, 40);
    R_Date("Beta-149260", 6330, 40);
    R_Date("R-996a", 6330, 70);
    R_Date("Beta-149259", 6350, 70);
    R_Date("R-1146", 6410, 70);
    R_Date("R-1147", 6480, 70);
    R_Date("R-1148", 6585, 80);
    R_Date("?", 6375, 35);
    R_Date("?", 6410, 35);
    R_Date("?", 6460, 35);
    R_Date("LTL-5000A", 6149, 45);
    R_Date("LTL-5001A", 6158, 55);
```

```
R_Date("R-468", 6575, 80);
R_Date("LTL-57a", 6579, 60);
R_Date("LTL-525a", 6651, 60);
R_Date("LTL-061a", 6688, 110);
R_Date("LTL-59a", 6718, 40);
R_Date("LTL-58a", 6727, 75);
R_Date("LTL-15953A", 6770, 45);
R_Date("LTL-15952A", 6809, 45);
R_Date("LTL-526a", 6823, 55);
R_Date("LTL-60a", 6843, 40);
R_Date("LTL15327a", 6225, 45);
R_Date("LTL15328a", 6311, 45);
R_Date("OxA-1958", 6540, 80);
R_Date("?", 6372, 90);
R_Date("Gif-4375", 6450, 90);
R_Date("R-1410", 6170, 75);
R_Date("R-2938", 6570, 63);
R_Date("R-1411", 6590, 75);
R_Date("PI-46", 6247, 130);
R_Date("R-676", 6080, 50);
R_Date("R-1167", 6860, 60);
R_Date("LTL-6123A", 6000, 45);
R_Date("?", 6152, 460);
R_Date("Lyon-5202", 6275, 45);
R_Date("LYON-3504", 6405, 35);
R_Date("LTL-6124A", 6505, 50);
R_Date("Rome-2978", 6189, 43);
R_Date("R-2310", 6310, 75);
R_Date("R-2309", 6350, 75);
R_Date("R-2357", 6350, 60);
R_Date("R-2311", 6370, 95);
R_Date("Rome-3563", 6414, 40);
R_Date("Rome-2979", 6437, 60);
R_Date("Rome-3338", 6487, 64);
R_Date("Rome-3327", 6494, 49);
R_Date("Rome-2569", 6498, 57);
R_Date("R-2345", 6530, 65);
R_Date("R-2336", 6550, 70);
R_Date("R-", 6565, 64);
R_Date("R-2339", 6590, 65);
R_Date("Rome-3573", 6594, 61);
R_Date("R-2353", 6600, 45);
R_Date("Rome-3331", 6622, 64);
R_Date("Rome-3557", 6627, 34);
R_Date("Rome-3558", 6852, 28);
R_Date("R-2360", 6855, 65);
R_Date("Rome-3572", 6874, 37);
R_Date("LTL-16673A", 6492, 65);
R_Date("LTL-16671A", 6637, 45);
R_Date("LTL-16672A", 6769, 45);
```

```
    R_Date("Ua-21097", 6440, 50);
    R_Date("R-643a", 6580, 75);
    R_Date("LTL078A", 6637, 83);
    R_Date("LTL-079A2", 6638, 59);
    R_Date("BM-2252R", 6000, 170);
    R_Date("BM-2251", 6250, 90);
    R_Date("BM-2250", 6290, 60);
    R_Date("BM-2251R", 6570, 140);
    R_Date("BM-2250R", 6590, 130);
    R_Date("Beta-44155", 6100, 80);
    R_Date("Beta-44114", 6180, 80);
    R_Date("LTL-2656A", 6168, 50);
    R_Date("LTL-2653A", 6624, 45);
    R_Date("LTL15330a", 6263, 45);
    R_Date("LTL15329a", 6397, 45);
    R_Date("R-427", 6210, 60);
    R_Date("X-45", 6580, 50);
    R_Date("R-2702", 6447, 56);
    R_Date("R-548", 6680, 80);
    R_Date("LTL18596A", 6076, 45);
    R_Date("LTL15989A", 6378, 45);
    R_Date("LTL5191a", 6418, 50);
    R_Date("?", 6500, 50);
    R_Date("LTL15991A", 6500, 45);
    R_Date("LTL5192A", 6500, 50);
    R_Date("LTL12777A", 6555, 45);
    R_Date("LTL 15357A", 6647, 45);
    R_Date("LTL15990a", 6673, 45);
    R_Date("LTL 15323A", 6734, 45);
    R_Date("LTL15358a", 6916, 45);
    R_Date("AECV-2011c", 6790, 70);
    R_Date("AECV-2012c", 6860, 60);
    R_Date("R-598a", 6140, 70);
    R_Date("R-598", 6210, 75);
    R_Date("R-599", 6260, 85);
    R_Date("R-599a", 6260, 85);
    R_Date("OxA-1852", 6245, 90);
    R_Date("OxA-1851", 6270, 70);
    R_Date("OxA-1853", 6430, 80);
    R_Date("OxA-1854", 6120, 90);
    R_Date("LTL2407A", 6116, 55);
    R_Date("LTL2910A", 6486, 65);
    R_Date("LTL2404A", 6593, 45);
    R_Date("Pi-101", 6578, 135);
   };
  Boundary("Central_Italy_neolithic_end");
  };
 };
```

Corsica

```
Plot(){
Sequence("Corsica_neolithic"){
 Sigma_Boundary("Corsica_neolithic_start");
  Phase("Corsica_neolithic") {
   Curve("IntCal20", "intcal20.14c");
    R_Date("LTL1154A", 6064, 55);
    R_Date("Ly-3008", 6255, 60);
    R_Date("Ly-3005", 6320, 70);
    R_Date("Ly-3007", 6370, 60);
    R_Date("Ly-3006", 6400, 70);
    R_Date("Ly-3009", 6420, 60);
    R_Date("?", 6430, 80);
    R_Date("GIF-7368", 6430, 130);
    R_Date("Ly-6085", 6280, 75);
    R_Date("GIF-2324", 6430, 140);
    R_Date("GIF-2325", 6650, 140);
    R_Date("Ly-842", 6840, 80);
    R_Date("LGQ-617", 6325, 140);
    R_Date("R-428", 6210, 80);
    R_Date("Ly-8328", 6095, 45);
    R_Date("Ly-11262", 6290, 40);
    R_Date("Ly-8327", 6525, 60);
    R_Date("Ly-9519", 6769, 41);
    R_Date("Ly-2835", 6420, 300);
    R_Date("Ly-2836", 6480, 480);
    R_Date("MC-2243", 6670, 130);
  };
 Sigma_Boundary("Corsica_neolithic_end");
 };
};

Plot(){
Sequence("Corsica_neolithic"){
 Boundary("Corsica_neolithic_start");
  Phase("Corsica_neolithic") {
   Curve("IntCal20", "intcal20.14c");
    R_Date("LTL1154A", 6064, 55);
    R_Date("Ly-3008", 6255, 60);
    R_Date("Ly-3005", 6320, 70);
    R_Date("Ly-3007", 6370, 60);
    R_Date("Ly-3006", 6400, 70);
    R_Date("Ly-3009", 6420, 60);
    R_Date("?", 6430, 80);
    R_Date("GIF-7368", 6430, 130);
    R_Date("Ly-6085", 6280, 75);
    R_Date("GIF-2324", 6430, 140);
    R_Date("GIF-2325", 6650, 140);
    R_Date("Ly-842", 6840, 80);
    R_Date("LGQ-617", 6325, 140);
```

```
    R_Date("R-428", 6210, 80);
    R_Date("Ly-8328", 6095, 45);
    R_Date("Ly-11262", 6290, 40);
    R_Date("Ly-8327", 6525, 60);
    R_Date("Ly-9519", 6769, 41);
    R_Date("Ly-2835", 6420, 300);
    R_Date("Ly-2836", 6480, 480);
    R_Date("MC-2243", 6670, 130);
  };
 Boundary("Corsica_neolithic_end");
 };
};
```

Malta:

```
Plot(){
Sequence("Malta_neolithic"){
 Sigma_Boundary("Malta_neolithic_start");
  Phase("Malta_neolithic") {
   Curve("IntCal20", "intcal20.14c");
    R_Date("UBA-31048", 6151, 33);
    R_Date("UBA-37689", 6171, 41);
    R_Date("UBA-31046", 6172, 39);
    R_Date("UBA-31039", 6175, 61);
    R_Date("UBA-31043", 6181, 40);
    R_Date("UBA-31037", 6193, 48);
    R_Date("UBA-31044", 6239, 37);
    R_Date("UBA-31047", 6267, 40);
    R_Date("UBA-37859", 6409, 38);
    R_Date("UBA-31042", 6412, 44);
    R_Date("UBA-33708", 6005, 51);
    R_Date("UBA-33709", 6035, 60);
    R_Date("BM-378", 6140, 160);
    R_Date("UBA-33710", 6158, 51);
  };
 Sigma_Boundary("Malta_neolithic_end");
 };
};

Plot(){
Sequence("Malta_neolithic"){
 Boundary("Malta_neolithic_start");
  Phase("Malta_neolithic") {
   Curve("IntCal20", "intcal20.14c");
    R_Date("UBA-31048", 6151, 33);
    R_Date("UBA-37689", 6171, 41);
    R_Date("UBA-31046", 6172, 39);
    R_Date("UBA-31039", 6175, 61);
    R_Date("UBA-31043", 6181, 40);
    R_Date("UBA-31037", 6193, 48);
```

```
    R_Date("UBA-31044", 6239, 37);
    R_Date("UBA-31047", 6267, 40);
    R_Date("UBA-37859", 6409, 38);
    R_Date("UBA-31042", 6412, 44);
    R_Date("UBA-33708", 6005, 51);
    R_Date("UBA-33709", 6035, 60);
    R_Date("BM-378", 6140, 160);
    R_Date("UBA-33710", 6158, 51);
  };
 Boundary("Malta_neolithic_end");
 };
};
```

Northern Italy

```
Plot(){
Sequence("Northern_Italy_neolithic"){
 Sigma_Boundary("Northern_Italy_neolithic_start");
  Phase("Northern_Italy_neolithic") {
   Curve("IntCal20", "intcal20.14c");
    R_Date("GX-20845", 6030, 80);
    R_Date("OxA-V-2365-34", 6029, 32);
    R_Date("Lyon-14596", 6095, 30);
    R_Date("Lyon-14591", 6105, 30);
    R_Date("OxA-V-2365-32", 6118, 33);
    R_Date("OxA-V-2365-35", 6155, 34);
    R_Date("Bln-3450", 6240, 90);
    R_Date("GrA-38257", 6315, 35);
    R_Date("OxA-V-2365-36", 6318, 33);
    R_Date("GrM-14531", 6447, 18);
    R_Date("GrM-15910", 6470, 30);
    R_Date("OxA-V-2365-50", 6669, 34);
    R_Date("Lyon-14595", 6670, 35);
    R_Date("Lyon-14594", 6675, 35);
    R_Date("OxA-V-2365-31", 6678, 33);
    R_Date("Lyon-14590", 6770, 30);
    R_Date("Lyon-14592", 6825, 35);
    R_Date("Lyon-14593", 6830, 35);
    R_Date("Beta-48691", 6000, 60);
    R_Date("MC-752", 6000, 120);
    R_Date("Beta-48692", 6010, 100);
    R_Date("Beta-60688", 6010, 110);
    R_Date("Beta-60689", 6050, 60);
    R_Date("LJ-4142", 6070, 90);
    R_Date("Beta-60707", 6090, 100);
    R_Date("LJ-4140", 6090, 90);
    R_Date("GrM-13679", 6135, 25);
    R_Date("Beta-60690", 6140, 160);
    R_Date("GrM-16975", 6145, 25);
    R_Date("Beta-66552", 6150, 70);
```

```
R_Date("UB-2420", 6205, 100);
R_Date("LJ-4141", 6220, 100);
R_Date("R-101", 6220, 55);
R_Date("LJ-4139", 6230, 90);
R_Date("Beta-60687", 6240, 110);
R_Date("LTL-16680A", 6271, 40);
R_Date("UB-2422", 6345, 180);
R_Date("Beta-66551", 6350, 60);
R_Date("Beta-109619", 6370, 50);
R_Date("LTL-6004A", 6446, 45);
R_Date("Pi-27bis", 6487, 175);
R_Date("LJ-4144", 6490, 100);
R_Date("LTL-16681A", 6623, 45);
R_Date("Beta-170555", 6700, 40);
R_Date("UB-2424", 6700, 145);
R_Date("LTL-15946A", 6750, 45);
R_Date("LTL-16678A", 6751, 45);
R_Date("OxA-23072", 6778, 39);
R_Date("Beta-110542", 6830, 40);
R_Date("LTL-15943A", 6834, 45);
R_Date("LTL-15944A", 6864, 45);
R_Date("LJ-4143", 6870, 100);
R_Date("Beta-66553", 6880, 60);
R_Date("LJ-4143bis", 6910, 110);
R_Date("UB-2423", 6980, 115);
R_Date("F-7", 6010, 130);
R_Date("R-316", 6015, 65);
R_Date("F-8", 6050, 130);
R_Date("R-263", 6140, 110);
R_Date("R-265", 6280, 120);
R_Date("R-315", 6280, 70);
R_Date("R-313A", 6400, 105);
R_Date("R-313", 6420, 65);
R_Date("R-267", 6470, 120);
R_Date("BAZZ 17", 6170, 40);
R_Date("BAZZ 13", 6280, 100);
R_Date("BAZZ 14", 6390, 40);
R_Date("GrM-16987", 6065, 80);
R_Date("LTL2427A", 6219, 50);
R_Date("GrM-13518", 6146, 25);
R_Date("GX29088", 6490, 90);
R_Date("GrM-12418", 6090, 18);
R_Date("OxA-X-2504-57", 6090, 55);
R_Date("OxA-21358", 6122, 38);
R_Date("OxA-27418", 6127, 35);
R_Date("I-11445", 6170, 110);
R_Date("GrM-15259", 6190, 40);
R_Date("I-11444", 6235, 470);
R_Date("LTL2435A", 6189, 43);
R_Date("LTL2436A", 6312, 50);
```

```
R_Date("Beta-71945", 6340, 60);
R_Date("I-12769", 6310, 105);
R_Date("I-12585", 6580, 150);
R_Date("GrM-14495", 6230, 25);
R_Date("OxA-19803", 6240, 30);
R_Date("R-458", 6000, 200);
R_Date("Bln-2795", 6090, 100);
R_Date("GrM-15257", 6130, 25);
R_Date("GrM-12416", 6184, 18);
R_Date("R-2313", 6100, 170);
R_Date("R-2314", 6280, 80);
R_Date("Bln-3372", 6320, 60);
R_Date("R-1544a", 6050, 90);
R_Date("R-2551", 6483, 74);
R_Date("R-2539", 6540, 70);
R_Date("R-2538", 6545, 70);
R_Date("R-2554", 6548, 85);
R_Date("R-2550", 6570, 75);
R_Date("GrN-19839", 6540, 60);
R_Date("GrN-19838", 6690, 180);
R_Date("AECV-1996C", 6380, 180);
R_Date("R-1038", 6290, 130);
R_Date("R-1040", 6300, 60);
R_Date("GrA-19820", 6390, 60);
R_Date("MC-2333", 6490, 110);
R_Date("MC-2332", 6510, 110);
R_Date("MC-758", 6000, 100);
R_Date("MC-759", 6050, 100);
R_Date("MC-757", 6580, 110);
R_Date("MC-756", 6950, 100);
R_Date("LTL-4120a", 6004, 45);
R_Date("Beta-244374", 6040, 50);
R_Date("Gx-20947", 6045, 95);
R_Date("Gx-20946", 6065, 80);
R_Date("LTL-4122a", 6247, 45);
R_Date("OxA-19737", 6183, 33);
R_Date("OxA-35333", 6290, 34);
R_Date("GX-23166", 6010, 270);
R_Date("GX-24967", 6000, 110);
R_Date("GrM-19458", 6130, 30);
R_Date("GrM-19457", 6165, 35);
R_Date("KIA30554", 6038, 31);
R_Date("GX-24940", 6650, 170);
R_Date("DSH-301", 6090, 41);
R_Date("DSA-728", 6134, 28);
R_Date("DSA-729", 6161, 22);
R_Date("DSH-302", 6210, 35);
R_Date("DSH-300", 6217, 26);
R_Date("DSA-737", 6223, 56);
R_Date("DSA-733", 6246, 24);
```

```
R_Date("DSA-734", 6272, 23);
R_Date("DSH-349", 6327, 27);
R_Date("DSH-299", 6356, 26);
R_Date("R-2745", 6524, 76);
R_Date("LTL-13440A", 6131, 50);
R_Date("LTL13440A", 6131, 50);
R_Date("R-2746", 6161, 39);
R_Date("Bln-3370", 6170, 50);
R_Date("OxA-19735", 6212, 32);
R_Date("OxA-19736", 6213, 33);
R_Date("OxA-23071", 6262, 37);
R_Date("LTL-13471A", 6335, 45);
R_Date("LTL13471A", 6335, 45);
R_Date("R-2748", 6585, 87);
R_Date("R-2747", 6626, 110);
R_Date("Birm-175", 6450, 110);
R_Date("Birm-177", 6125, 150);
R_Date("Birm-172", 6240, 100);
R_Date("Birm-173", 6290, 150);
R_Date("Birm-174", 6350, 140);
R_Date("Birm-176", 6470, 110);
R_Date("AA-66517", 6212, 71);
R_Date("AA-66515", 6222, 44);
R_Date("AA-62121", 6110, 48);
R_Date("AA-62122", 6146, 49);
R_Date("AA-62124", 6231, 48);
R_Date("GX-24945", 6440, 90);
R_Date("LTL-17274a", 6097, 45);
R_Date("LTL-950a", 6069, 55);
R_Date("LTL-4253A", 6077, 40);
R_Date("Hd-23489", 6106, 32);
R_Date("LTL-4250A", 6106, 40);
R_Date("LTL-4249A", 6112, 45);
R_Date("LTL-4247A", 6127, 40);
R_Date("OZC-210", 6060, 60);
R_Date("LTL-1543a", 6091, 55);
R_Date("OZB-950", 6240, 80);
R_Date("OZB-949", 6280, 80);
R_Date("OZC-211", 6330, 50);
R_Date("OZB-94B", 6410, 92);
R_Date("R-2705", 6751, 108);
R_Date("Lyon-14605(SacA-51548)", 6015, 30);
R_Date("Lyon-14606(SacA-51549)", 6040, 30);
R_Date("GrM-13355", 6092, 30);
R_Date("GrM-15883", 6095, 35);
R_Date("B-5088", 6060, 50);
R_Date("B-5090", 6320, 80);
R_Date("LTL-4562A", 6422, 50);
R_Date("LTL-13068A", 6020, 45);
R_Date("LTL-13066A", 6051, 45);
```

```
   R_Date("R-781a", 6060, 50);
   R_Date("R-1136", 6480, 50);
   R_Date("Bln-1777", 6030, 45);
   R_Date("Gif-3766", 6550, 150);
   R_Date("X-27", 6060, 50);
   R_Date("I-12519", 6070, 110);
   R_Date("OxA-19734", 6675, 29);
   R_Date("GrA-25715", 6760, 45);
   R_Date("OxA-21359", 6767, 39);
   R_Date("LTL-944a", 6000, 40);
   R_Date("LtL-959a", 6027, 50);
   R_Date("R-2735", 6028, 53);
   R_Date("LtL-262a", 6059, 50);
   R_Date("LTL-967a", 6060, 50);
   R_Date("LTL-955a", 6063, 50);
   R_Date("LTL-1504a", 6110, 65);
   R_Date("BLN-3373", 6120, 60);
   R_Date("Hd-23544", 6128, 21);
   R_Date("HD-23536", 6129, 20);
   R_Date("R-2743", 6170, 57);
   R_Date("LTL-956a", 6231, 60);
   R_Date("R-2548", 6513, 70);
   R_Date("R-2549", 6525, 55);
   R_Date("R-2545", 6535, 75);
   R_Date("R-2547", 6570, 74);
   R_Date("I- 1289", 6310, 210);
   R_Date("LTL5251A", 6316, 45);
   R_Date("LTL5250A", 6327, 45);
   R_Date("Lyon-14608(SacA-51551)", 6075, 30);
   R_Date("Hd-23010", 6011, 26);
   R_Date("I-13798", 6130, 160);
   R_Date("Bln-4432", 6400, 200);
   R_Date("Bln-4431", 6504, 59);
   R_Date("Bln-4436", 6557, 71);
   R_Date("I-12518", 6050, 110);
   R_Date("LTL-13656A", 6168, 45);
   R_Date("LTL-13654a", 6258, 45);
   R_Date("OxA-19732", 6016, 31);
   R_Date("OxA-19733", 6038, 32);
   R_Date("ETH-30058", 6050, 60);
   R_Date("ETH-30059", 6105, 60);
   R_Date("ETH-30057", 6920, 60);
  };
 Sigma_Boundary("Northern_Italy_neolithic_end");
 };
};

Plot(){
Sequence("Northern_Italy_neolithic"){
 Boundary("Northern_Italy_neolithic_start");
```

```
Phase("Northern_Italy_neolithic") {
 Curve("IntCal20", "intcal20.14c");
  R_Date("GX-20845", 6030, 80);
  R_Date("OxA-V-2365-34", 6029, 32);
  R_Date("Lyon-14596", 6095, 30);
  R_Date("Lyon-14591", 6105, 30);
  R_Date("OxA-V-2365-32", 6118, 33);
  R_Date("OxA-V-2365-35", 6155, 34);
  R_Date("Bln-3450", 6240, 90);
  R_Date("GrA-38257", 6315, 35);
  R_Date("OxA-V-2365-36", 6318, 33);
  R_Date("GrM-14531", 6447, 18);
  R_Date("GrM-15910", 6470, 30);
  R_Date("OxA-V-2365-50", 6669, 34);
  R_Date("Lyon-14595", 6670, 35);
  R_Date("Lyon-14594", 6675, 35);
  R_Date("OxA-V-2365-31", 6678, 33);
  R_Date("Lyon-14590", 6770, 30);
  R_Date("Lyon-14592", 6825, 35);
  R_Date("Lyon-14593", 6830, 35);
  R_Date("Beta-48691", 6000, 60);
  R_Date("MC-752", 6000, 120);
  R_Date("Beta-48692", 6010, 100);
  R_Date("Beta-60688", 6010, 110);
  R_Date("Beta-60689", 6050, 60);
  R_Date("LJ-4142", 6070, 90);
  R_Date("Beta-60707", 6090, 100);
  R_Date("LJ-4140", 6090, 90);
  R_Date("GrM-13679", 6135, 25);
  R_Date("Beta-60690", 6140, 160);
  R_Date("GrM-16975", 6145, 25);
  R_Date("Beta-66552", 6150, 70);
  R_Date("UB-2420", 6205, 100);
  R_Date("LJ-4141", 6220, 100);
  R_Date("R-101", 6220, 55);
  R_Date("LJ-4139", 6230, 90);
  R_Date("Beta-60687", 6240, 110);
  R_Date("LTL-16680A", 6271, 40);
  R_Date("UB-2422", 6345, 180);
  R_Date("Beta-66551", 6350, 60);
  R_Date("Beta-109619", 6370, 50);
  R_Date("LTL-6004A", 6446, 45);
  R_Date("Pi-27bis", 6487, 175);
  R_Date("LJ-4144", 6490, 100);
  R_Date("LTL-16681A", 6623, 45);
  R_Date("Beta-170555", 6700, 40);
  R_Date("UB-2424", 6700, 145);
  R_Date("LTL-15946A", 6750, 45);
  R_Date("LTL-16678A", 6751, 45);
  R_Date("OxA-23072", 6778, 39);
```

```
R_Date("Beta-110542", 6830, 40);
R_Date("LTL-15943A", 6834, 45);
R_Date("LTL-15944A", 6864, 45);
R_Date("LJ-4143", 6870, 100);
R_Date("Beta-66553", 6880, 60);
R_Date("LJ-4143bis", 6910, 110);
R_Date("UB-2423", 6980, 115);
R_Date("F-7", 6010, 130);
R_Date("R-316", 6015, 65);
R_Date("F-8", 6050, 130);
R_Date("R-263", 6140, 110);
R_Date("R-265", 6280, 120);
R_Date("R-315", 6280, 70);
R_Date("R-313A", 6400, 105);
R_Date("R-313", 6420, 65);
R_Date("R-267", 6470, 120);
R_Date("BAZZ 17", 6170, 40);
R_Date("BAZZ 13", 6280, 100);
R_Date("BAZZ 14", 6390, 40);
R_Date("GrM-16987", 6065, 80);
R_Date("LTL2427A", 6219, 50);
R_Date("GrM-13518", 6146, 25);
R_Date("GX29088", 6490, 90);
R_Date("GrM-12418", 6090, 18);
R_Date("OxA-X-2504-57", 6090, 55);
R_Date("OxA-21358", 6122, 38);
R_Date("OxA-27418", 6127, 35);
R_Date("I-11445", 6170, 110);
R_Date("GrM-15259", 6190, 40);
R_Date("I-11444", 6235, 470);
R_Date("LTL2435A", 6189, 43);
R_Date("LTL2436A", 6312, 50);
R_Date("Beta-71945", 6340, 60);
R_Date("I-12769", 6310, 105);
R_Date("I-12585", 6580, 150);
R_Date("GrM-14495", 6230, 25);
R_Date("OxA-19803", 6240, 30);
R_Date("R-458", 6000, 200);
R_Date("Bln-2795", 6090, 100);
R_Date("GrM-15257", 6130, 25);
R_Date("GrM-12416", 6184, 18);
R_Date("R-2313", 6100, 170);
R_Date("R-2314", 6280, 80);
R_Date("Bln-3372", 6320, 60);
R_Date("R-1544a", 6050, 90);
R_Date("R-2551", 6483, 74);
R_Date("R-2539", 6540, 70);
R_Date("R-2538", 6545, 70);
R_Date("R-2554", 6548, 85);
R_Date("R-2550", 6570, 75);
```

```
R_Date("GrN-19839", 6540, 60);
R_Date("GrN-19838", 6690, 180);
R_Date("AECV-1996C", 6380, 180);
R_Date("R-1038", 6290, 130);
R_Date("R-1040", 6300, 60);
R_Date("GrA-19820", 6390, 60);
R_Date("MC-2333", 6490, 110);
R_Date("MC-2332", 6510, 110);
R_Date("MC-758", 6000, 100);
R_Date("MC-759", 6050, 100);
R_Date("MC-757", 6580, 110);
R_Date("MC-756", 6950, 100);
R_Date("LTL-4120a", 6004, 45);
R_Date("Beta-244374", 6040, 50);
R_Date("Gx-20947", 6045, 95);
R_Date("Gx-20946", 6065, 80);
R_Date("LTL-4122a", 6247, 45);
R_Date("OxA-19737", 6183, 33);
R_Date("OxA-35333", 6290, 34);
R_Date("GX-23166", 6010, 270);
R_Date("GX-24967", 6000, 110);
R_Date("GrM-19458", 6130, 30);
R_Date("GrM-19457", 6165, 35);
R_Date("KIA30554", 6038, 31);
R_Date("GX-24940", 6650, 170);
R_Date("DSH-301", 6090, 41);
R_Date("DSA-728", 6134, 28);
R_Date("DSA-729", 6161, 22);
R_Date("DSH-302", 6210, 35);
R_Date("DSH-300", 6217, 26);
R_Date("DSA-737", 6223, 56);
R_Date("DSA-733", 6246, 24);
R_Date("DSA-734", 6272, 23);
R_Date("DSH-349", 6327, 27);
R_Date("DSH-299", 6356, 26);
R_Date("R-2745", 6524, 76);
R_Date("LTL-13440A", 6131, 50);
R_Date("LTL13440A", 6131, 50);
R_Date("R-2746", 6161, 39);
R_Date("Bln-3370", 6170, 50);
R_Date("OxA-19735", 6212, 32);
R_Date("OxA-19736", 6213, 33);
R_Date("OxA-23071", 6262, 37);
R_Date("LTL-13471A", 6335, 45);
R_Date("LTL13471A", 6335, 45);
R_Date("R-2748", 6585, 87);
R_Date("R-2747", 6626, 110);
R_Date("Birm-175", 6450, 110);
R_Date("Birm-177", 6125, 150);
R_Date("Birm-172", 6240, 100);
```

```
R_Date("Birm-173", 6290, 150);
R_Date("Birm-174", 6350, 140);
R_Date("Birm-176", 6470, 110);
R_Date("AA-66517", 6212, 71);
R_Date("AA-66515", 6222, 44);
R_Date("AA-62121", 6110, 48);
R_Date("AA-62122", 6146, 49);
R_Date("AA-62124", 6231, 48);
R_Date("GX-24945", 6440, 90);
R_Date("LTL-17274a", 6097, 45);
R_Date("LTL-950a", 6069, 55);
R_Date("LTL-4253A", 6077, 40);
R_Date("Hd-23489", 6106, 32);
R_Date("LTL-4250A", 6106, 40);
R_Date("LTL-4249A", 6112, 45);
R_Date("LTL-4247A", 6127, 40);
R_Date("OZC-210", 6060, 60);
R_Date("LTL-1543a", 6091, 55);
R_Date("OZB-950", 6240, 80);
R_Date("OZB-949", 6280, 80);
R_Date("OZC-211", 6330, 50);
R_Date("OZB-94B", 6410, 92);
R_Date("R-2705", 6751, 108);
R_Date("Lyon-14605(SacA-51548)", 6015, 30);
R_Date("Lyon-14606(SacA-51549)", 6040, 30);
R_Date("GrM-13355", 6092, 30);
R_Date("GrM-15883", 6095, 35);
R_Date("B-5088", 6060, 50);
R_Date("B-5090", 6320, 80);
R_Date("LTL-4562A", 6422, 50);
R_Date("LTL-13068A", 6020, 45);
R_Date("LTL-13066A", 6051, 45);
R_Date("R-781a", 6060, 50);
R_Date("R-1136", 6480, 50);
R_Date("Bln-1777", 6030, 45);
R_Date("Gif-3766", 6550, 150);
R_Date("X-27", 6060, 50);
R_Date("I-12519", 6070, 110);
R_Date("OxA-19734", 6675, 29);
R_Date("GrA-25715", 6760, 45);
R_Date("OxA-21359", 6767, 39);
R_Date("LTL-944a", 6000, 40);
R_Date("LtL-959a", 6027, 50);
R_Date("R-2735", 6028, 53);
R_Date("LtL-262a", 6059, 50);
R_Date("LTL-967a", 6060, 50);
R_Date("LTL-955a", 6063, 50);
R_Date("LTL-1504a", 6110, 65);
R_Date("BLN-3373", 6120, 60);
R_Date("Hd-23544", 6128, 21);
```

```
   R_Date("HD-23536", 6129, 20);
   R_Date("R-2743", 6170, 57);
   R_Date("LTL-956a", 6231, 60);
   R_Date("R-2548", 6513, 70);
   R_Date("R-2549", 6525, 55);
   R_Date("R-2545", 6535, 75);
   R_Date("R-2547", 6570, 74);
   R_Date("I- 1289", 6310, 210);
   R_Date("LTL5251A", 6316, 45);
   R_Date("LTL5250A", 6327, 45);
   R_Date("Lyon-14608(SacA-51551)", 6075, 30);
   R_Date("Hd-23010", 6011, 26);
   R_Date("I-13798", 6130, 160);
   R_Date("Bln-4432", 6400, 200);
   R_Date("Bln-4431", 6504, 59);
   R_Date("Bln-4436", 6557, 71);
   R_Date("I-12518", 6050, 110);
   R_Date("LTL-13656A", 6168, 45);
   R_Date("LTL-13654a", 6258, 45);
   R_Date("OxA-19732", 6016, 31);
   R_Date("OxA-19733", 6038, 32);
   R_Date("ETH-30058", 6050, 60);
   R_Date("ETH-30059", 6105, 60);
   R_Date("ETH-30057", 6920, 60);
  };
 Boundary("Northern_Italy_neolithic_end");
 };
};
```

Sardinia

```
Plot(){
Sequence("Sardinia_neolithic"){
 Sigma_Boundary("Sardinia_neolithic_start");
  Phase("Sardinia_neolithic") {
  Curve("IntCal20", "intcal20.14c");
   R_Date("AA-80543", 6040, 80);
   R_Date("Q-3024", 6120, 55);
   R_Date("Q-3023", 6470, 65);
   R_Date("Q-3022", 6515, 65);
   R_Date("Q-3021", 6615, 75);
   R_Date("GrN-11433", 6260, 180);
   R_Date("UtC-15233", 6490, 90);
   R_Date("UtC-1251", 6690, 80);
   R_Date("Q-3020", 6710, 75);
   R_Date("Beta-334478", 6200, 40);
   R_Date("Beta-334480", 6250, 40);
   R_Date("Beta-334479", 6430, 40);
   R_Date("Beta-167929", 6400, 40);
   R_Date("Beta-167930", 6680, 160);
```

```
    R_Date("Beta-167931", 6830, 80);
    R_Date("Ly-3010", 6230, 60);
    R_Date("AA-58899", 6266, 48);
    R_Date("AA-65493", 6325, 86);
    R_Date("AA-65497", 6652, 55);
    R_Date("AA-6597", 6652, 55);
    R_Date("Ly-3011", 6370, 60);
    R_Date("Ly-3012", 6440, 60);
    R_Date("AA-75653", 6327, 41);
    R_Date("AA-75654", 6341, 46);
    R_Date("AA-80547", 6359, 43);
  };
 Sigma_Boundary("Sardinia_neolithic_end");
 };
};

Plot(){
Sequence("Sardinia_neolithic"){
 Boundary("Sardinia_neolithic_start");
  Phase("Sardinia_neolithic") {
   Curve("IntCal20", "intcal20.14c");
    R_Date("AA-80543", 6040, 80);
    R_Date("Q-3024", 6120, 55);
    R_Date("Q-3023", 6470, 65);
    R_Date("Q-3022", 6515, 65);
    R_Date("Q-3021", 6615, 75);
    R_Date("GrN-11433", 6260, 180);
    R_Date("UtC-15233", 6490, 90);
    R_Date("UtC-1251", 6690, 80);
    R_Date("Q-3020", 6710, 75);
    R_Date("Beta-334478", 6200, 40);
    R_Date("Beta-334480", 6250, 40);
    R_Date("Beta-334479", 6430, 40);
    R_Date("Beta-167929", 6400, 40);
    R_Date("Beta-167930", 6680, 160);
    R_Date("Beta-167931", 6830, 80);
    R_Date("Ly-3010", 6230, 60);
    R_Date("AA-58899", 6266, 48);
    R_Date("AA-65493", 6325, 86);
    R_Date("AA-65497", 6652, 55);
    R_Date("AA-6597", 6652, 55);
    R_Date("Ly-3011", 6370, 60);
    R_Date("Ly-3012", 6440, 60);
    R_Date("AA-75653", 6327, 41);
    R_Date("AA-75654", 6341, 46);
    R_Date("AA-80547", 6359, 43);
  };
 Boundary("Sardinia_neolithic_end");
 };
};
```

Sicily (alternative labels)

```
Plot(){
Sequence("SicilyAlt_neolithic"){
 Sigma_Boundary("SicilyAlt_neolithic_start");
  Phase("SicilyAlt_neolithic") {
   Curve("IntCal20", "intcal20.14c");
    R_Date("LTL15581A", 6476, 45);
    R_Date("R-432", 6200, 80);
    R_Date("MAMS-40723", 6286, 24);
    R_Date("MAMS-40716", 6310, 23);
    R_Date("MAMS-40717", 6310, 23);
    R_Date("MAMS-40725", 6351, 24);
    R_Date("MAMS-48213", 6446, 25);
    R_Date("UD-165", 6720, 80);
    R_Date("P-2733", 6750, 70);
    Curve("Marine20", "marine20.14c");
    Delta_R("KIA-36035", 88, 50);
    R_Date("KIA-36035", 6985, 40);
    Curve("IntCal20", "intcal20.14c");
    R_Date("OxA-V-2364-40", 7006, 34);
    Curve("Marine20", "marine20.14c");
    Delta_R("KIA-36033", 88, 50);
    R_Date("KIA-36033", 7020, 45);
    Curve("IntCal20", "intcal20.14c");
    R_Date("MAMS-40712", 7036, 25);
    R_Date("MAMS-48212", 7051, 27);
    Curve("Marine20", "marine20.14c");
    Delta_R("OxA-13808", 88, 50);
    R_Date("OxA-13808", 7173, 37);
    Delta_R("KIA-36032", 88, 50);
    R_Date("KIA-36032", 7175, 45);
    Delta_R("OxA-13661", 88, 50);
    R_Date("OxA-13661", 7410, 32);
    Delta_R("OxA-X-2071-31", 88, 50);
    R_Date("OxA-X-2071-31", 7413, 39);
    Curve("IntCal20", "intcal20.14c");
    R_Date("LTL-211A", 6991, 60);
    R_Date("A-4474", 6130, 90);
    R_Date("?", 6335, 192);
    R_Date("CAN-2396", 6000, 45);
    R_Date("CAN-2398", 6065, 25);
    R_Date("R-292", 6260, 110);
    R_Date("R-291", 6630, 120);
    R_Date("Beta-408858", 6340, 30);
  };
 Sigma_Boundary("SicilyAlt_neolithic_end");
 };
};
```

```
Plot(){
Sequence("SicilyAlt_neolithic"){
 Boundary("SicilyAlt_neolithic_start");
  Phase("SicilyAlt_neolithic") {
   Curve("IntCal20", "intcal20.14c");
    R_Date("LTL15581A", 6476, 45);
    R_Date("R-432", 6200, 80);
    R_Date("MAMS-40723", 6286, 24);
    R_Date("MAMS-40716", 6310, 23);
    R_Date("MAMS-40717", 6310, 23);
    R_Date("MAMS-40725", 6351, 24);
    R_Date("MAMS-48213", 6446, 25);
    R_Date("UD-165", 6720, 80);
    R_Date("P-2733", 6750, 70);
    Curve("Marine20", "marine20.14c");
    Delta_R("KIA-36035", 88, 50);
    R_Date("KIA-36035", 6985, 40);
    Curve("IntCal20", "intcal20.14c");
    R_Date("OxA-V-2364-40", 7006, 34);
    Curve("Marine20", "marine20.14c");
    Delta_R("KIA-36033", 88, 50);
    R_Date("KIA-36033", 7020, 45);
    Curve("IntCal20", "intcal20.14c");
    R_Date("MAMS-40712", 7036, 25);
    R_Date("MAMS-48212", 7051, 27);
    Curve("Marine20", "marine20.14c");
    Delta_R("OxA-13808", 88, 50);
    R_Date("OxA-13808", 7173, 37);
    Delta_R("KIA-36032", 88, 50);
    R_Date("KIA-36032", 7175, 45);
    Delta_R("OxA-13661", 88, 50);
    R_Date("OxA-13661", 7410, 32);
    Delta_R("OxA-X-2071-31", 88, 50);
    R_Date("OxA-X-2071-31", 7413, 39);
    Curve("IntCal20", "intcal20.14c");
    R_Date("LTL-211A", 6991, 60);
    R_Date("A-4474", 6130, 90);
    R_Date("?", 6335, 192);
    R_Date("CAN-2396", 6000, 45);
    R_Date("CAN-2398", 6065, 25);
    R_Date("R-292", 6260, 110);
    R_Date("R-291", 6630, 120);
    R_Date("Beta-408858", 6340, 30);
  };
 Boundary("SicilyAlt_neolithic_end");
 };
};
```

Southern Italy

```
Plot(){
Sequence("Southern_Italy_neolithic"){
 Sigma_Boundary("Southern_Italy_neolithic_start");
  Phase("Southern_Italy_neolithic") {
   Curve("IntCal20", "intcal20.14c");
    R_Date("P-2949", 6710, 80);
    R_Date("LTL-139A", 6148, 45);
    R_Date("LTL-140A", 6523, 45);
    R_Date("KIA*", 6565, 29);
    R_Date("KIA*", 6602, 30);
    R_Date("P-3047", 6430, 90);
    R_Date("Eth-21724", 6080, 65);
    R_Date("SUERC-4535", 6180, 35);
    R_Date("SUERC-4536", 6415, 35);
    R_Date("OxA-1474", 6850, 80);
    R_Date("OxA-1475", 6880, 90);
    R_Date("LTL-2718A", 6035, 60);
    R_Date("LTL-2720A", 6178, 55);
    R_Date("LTL-4577A", 6290, 45);
    R_Date("LTL-437A", 6334, 50);
    R_Date("LTL-2716A", 6341, 55);
    R_Date("LTL-438A", 6417, 55);
    R_Date("Beta-80603", 6540, 60);
    R_Date("LTL-2717A", 6551, 40);
    R_Date("Beta-80604", 6570, 70);
    R_Date("Beta 80604", 6630, 70);
    R_Date("UtC-1411", 6630, 40);
    R_Date("Beta-161933", 6650, 50);
    R_Date("Beta-71144", 6650, 70);
    R_Date("Beta 71144", 6670, 70);
    R_Date("Beta-71143", 6740, 80);
    R_Date("LTL-4500A", 6741, 50);
    R_Date("Utc-1342", 6990, 80);
    R_Date("LTL-204A", 6793, 40);
    R_Date("Beta-7163", 6860, 60);
    R_Date("LTL-203A", 6890, 50);
    R_Date("Beta-71633", 6910, 60);
    R_Date("Beta-165482", 6940, 40);
    R_Date("LTL-202A", 6956, 65);
    R_Date("LTL-778A", 7003, 55);
    R_Date("BM-2415", 6490, 150);
    R_Date("SUERC- 77407", 6570, 23);
    R_Date("SUERC- 77406", 6594, 23);
    R_Date("OxA-7596", 6555, 50);
    R_Date("AAR25755", 6072, 36);
    R_Date("LTL-201a", 6261, 40);
    R_Date("LTL-2155A", 6808, 50);
    R_Date("LJ-4983", 6120, 80);
    R_Date("LJ-5096", 6290, 80);
```

```
R_Date("LJ-5097", 6290, 90);
R_Date("OxA-21200", 6303, 33);
R_Date("OxA-21207", 6324, 32);
R_Date("OxA-21208", 6329, 31);
R_Date("LJ-4651", 6330, 90);
R_Date("OxA-21203", 6339, 33);
R_Date("OxA-21084", 6347, 32);
R_Date("OxA-21201", 6347, 33);
R_Date("OxA-21083", 6348, 31);
R_Date("OxA-21209", 6368, 31);
R_Date("OxA-21211", 6371, 33);
R_Date("OxA-21205", 6381, 33);
R_Date("OxA-21202", 6384, 33);
R_Date("OxA-21206", 6384, 32);
R_Date("OxA-X-2336-13", 6398, 34);
R_Date("LJ 5095", 6400, 80);
R_Date("LJ-5095", 6400, 80);
R_Date("LJ-4980", 6410, 150);
R_Date("OxA-21204", 6411, 32);
R_Date("OxA-21210", 6448, 31);
R_Date("LJ-4650", 6490, 140);
R_Date("LJ-4981", 6530, 260);
R_Date("LJ-4649", 6720, 100);
R_Date("OxA-7594", 6345, 45);
R_Date("OxA-7595", 6375, 50);
R_Date("BM-2258R", 6020, 160);
R_Date("BM-2256R", 6120, 170);
R_Date("LTL-2659A", 6320, 50);
R_Date("LTL-2658A", 6385, 50);
R_Date("LTL-2657A", 6562, 50);
R_Date("UCLA-2148", 6700, 100);
R_Date("UB-2271", 6790, 255);
R_Date("LTL-1212A", 6542, 45);
R_Date("OxA-3683", 6200, 95);
R_Date("OxA-10013", 6450, 50);
R_Date("OxA-12064", 6501, 37);
R_Date("OxA-3685", 6510, 95);
R_Date("OxA-9989", 6510, 45);
R_Date("OxA-9990", 6555, 45);
R_Date("OxA-12063", 6601, 37);
R_Date("OxA-9988", 6605, 45);
R_Date("OxA-12062", 6638, 34);
R_Date("OxA-3684", 6640, 95);
R_Date("?", 6800, 80);
R_Date("MC-2292", 7124, 200);
R_Date("OxA-653", 6210, 70);
R_Date("OxA-652", 6280, 70);
R_Date("OxA-651", 6540, 80);
R_Date("LTL-1060A", 6352, 60);
R_Date("LTL-1062A", 6442, 50);
```

```
R_Date("-LTL 1061,00", 6600, 60);
R_Date("R-322", 6780, 90);
R_Date("LTL-5188A", 6561, 50);
R_Date("LTL-5187A", 6576, 45);
R_Date("R-846", 6140, 120);
R_Date("Beta 181785", 6340, 40);
R_Date("Beta-169498", 6340, 40);
R_Date("Beta-181784", 6410, 40);
R_Date("P-2946", 6930, 60);
R_Date("F-97", 6465, 185);
R_Date("LTL-427A", 6739, 60);
R_Date("LTL-141A", 6651, 50);
R_Date("LTL-3810A", 6764, 60);
R_Date("LTL-4536A", 6983, 50);
R_Date("LTL-142A", 7134, 60);
R_Date("LJ-150", 6530, 150);
R_Date("LJ-4550", 6530, 150);
R_Date("LJ-4549", 6760, 100);
R_Date("LJ-4551", 6900, 150);
R_Date("LJ-4548", 7110, 140);
R_Date("CAMS-2681", 6890, 60);
R_Date("LTL-16676A", 6910, 40);
R_Date("LTL-16677A", 6988, 45);
R_Date("LTL-1727A", 6277, 6277);
R_Date("AAR25751", 6072, 34);
R_Date("Gif-6722", 6530, 70);
R_Date("Gif-6724", 6980, 70);
R_Date("BM-2414", 6520, 70);
R_Date("LTL2154A", 6229, 55);
R_Date("LTL2156A", 6432, 60);
R_Date("Gif-7057", 6040, 70);
R_Date("Gif-7346", 6320, 80);
R_Date("Gif-7345", 6600, 120);
R_Date("Gif-7055", 6810, 80);
R_Date("R-351", 6540, 65);
R_Date("R-350", 7000, 100);
R_Date("LTL147A", 6410, 80);
R_Date("KIA10827", 6679, 54);
R_Date("LTL026A", 6762, 55);
R_Date("LTL221A", 6785, 40);
R_Date("Beta-59933", 6870, 70);
R_Date("Gif-6725", 6900, 80);
R_Date("Ly-4002", 6590, 140);
R_Date("LJ-1448", 6860, 45);
R_Date("TAN-88247", 6890, 130);
R_Date("TAN-88066", 6960, 130);
R_Date("X-53", 6960, 130);
R_Date("UGAMS-8552", 6060, 30);
R_Date("LTL-12139A", 6301, 45);
R_Date("LTL-12145A", 6612, 45);
```

```
   R_Date("LTL-12146A", 6614, 45);
   R_Date("LTL-12144A", 6632, 45);
   R_Date("LTL-12142A", 6644, 45);
   R_Date("LTL-12143A", 6683, 45);
   R_Date("TAN-88068", 6660, 150);
   R_Date("Ly-3948", 6710, 35);
   R_Date("Lyon-4408", 6710, 180);
   R_Date("Ly-3949", 6730, 40);
   R_Date("Ly-3953", 6730, 40);
   R_Date("Ly-3952", 6760, 40);
   R_Date("TAN-88313", 6790, 120);
   R_Date("Ly-3950", 6810, 35);
   R_Date("Lyon-3896", 6810, 150);
   R_Date("Lyon-4410", 6830, 190);
   R_Date("Ly-3954", 6835, 40);
   R_Date("Ly-3951", 6840, 35);
   R_Date("Ly-3955", 6935, 40);
   R_Date("Lyon-5300", 6940, 270);
   R_Date("Lyon-4409", 6950, 190);
   R_Date("Lyon-5296", 6950, 150);
   R_Date("TAN-88056", 6950, 140);
   R_Date("TAN-88067", 6950, 130);
   R_Date("TAN-88248", 6980, 130);
   R_Date("Lyon-5297", 7030, 160);
   R_Date("Beta-169499", 6190, 40);
   R_Date("OxA-23122", 6432, 33);
   R_Date("OxA-23121", 6448, 30);
   R_Date("OxA-23119", 6452, 35);
   R_Date("OxA-23117", 6469, 32);
   R_Date("OxA-23118", 6484, 33);
   R_Date("OxA-23120", 6526, 34);
   R_Date("Beta-135147", 6620, 60);
   R_Date("Beta-122939", 6750, 50);
   R_Date("MC-2291", 6750, 220);
   R_Date("MC-2290", 6850, 130);
  };
 Sigma_Boundary("Southern_Italy_neolithic_end");
 };
};

Plot(){
Sequence("Southern_Italy_neolithic"){
 Boundary("Southern_Italy_neolithic_start");
  Phase("Southern_Italy_neolithic") {
   Curve("IntCal20", "intcal20.14c");
   R_Date("P-2949", 6710, 80);
   R_Date("LTL-139A", 6148, 45);
   R_Date("LTL-140A", 6523, 45);
   R_Date("KIA*", 6565, 29);
   R_Date("KIA*", 6602, 30);
```

```
R_Date("P-3047", 6430, 90);
R_Date("Eth-21724", 6080, 65);
R_Date("SUERC-4535", 6180, 35);
R_Date("SUERC-4536", 6415, 35);
R_Date("OxA-1474", 6850, 80);
R_Date("OxA-1475", 6880, 90);
R_Date("LTL-2718A", 6035, 60);
R_Date("LTL-2720A", 6178, 55);
R_Date("LTL-4577A", 6290, 45);
R_Date("LTL-437A", 6334, 50);
R_Date("LTL-2716A", 6341, 55);
R_Date("LTL-438A", 6417, 55);
R_Date("Beta-80603", 6540, 60);
R_Date("LTL-2717A", 6551, 40);
R_Date("Beta-80604", 6570, 70);
R_Date("Beta 80604", 6630, 70);
R_Date("UtC-1411", 6630, 40);
R_Date("Beta-161933", 6650, 50);
R_Date("Beta-71144", 6650, 70);
R_Date("Beta 71144", 6670, 70);
R_Date("Beta-71143", 6740, 80);
R_Date("LTL-4500A", 6741, 50);
R_Date("Utc-1342", 6990, 80);
R_Date("LTL-204A", 6793, 40);
R_Date("Beta-7163", 6860, 60);
R_Date("LTL-203A", 6890, 50);
R_Date("Beta-71633", 6910, 60);
R_Date("Beta-165482", 6940, 40);
R_Date("LTL-202A", 6956, 65);
R_Date("LTL-778A", 7003, 55);
R_Date("BM-2415", 6490, 150);
R_Date("SUERC- 77407", 6570, 23);
R_Date("SUERC- 77406", 6594, 23);
R_Date("OxA-7596", 6555, 50);
R_Date("AAR25755", 6072, 36);
R_Date("LTL-201a", 6261, 40);
R_Date("LTL-2155A", 6808, 50);
R_Date("LJ-4983", 6120, 80);
R_Date("LJ-5096", 6290, 80);
R_Date("LJ-5097", 6290, 90);
R_Date("OxA-21200", 6303, 33);
R_Date("OxA-21207", 6324, 32);
R_Date("OxA-21208", 6329, 31);
R_Date("LJ-4651", 6330, 90);
R_Date("OxA-21203", 6339, 33);
R_Date("OxA-21084", 6347, 32);
R_Date("OxA-21201", 6347, 33);
R_Date("OxA-21083", 6348, 31);
R_Date("OxA-21209", 6368, 31);
R_Date("OxA-21211", 6371, 33);
```

```
R_Date("OxA-21205", 6381, 33);
R_Date("OxA-21202", 6384, 33);
R_Date("OxA-21206", 6384, 32);
R_Date("OxA-X-2336-13", 6398, 34);
R_Date("LJ 5095", 6400, 80);
R_Date("LJ-5095", 6400, 80);
R_Date("LJ-4980", 6410, 150);
R_Date("OxA-21204", 6411, 32);
R_Date("OxA-21210", 6448, 31);
R_Date("LJ-4650", 6490, 140);
R_Date("LJ-4981", 6530, 260);
R_Date("LJ-4649", 6720, 100);
R_Date("OxA-7594", 6345, 45);
R_Date("OxA-7595", 6375, 50);
R_Date("BM-2258R", 6020, 160);
R_Date("BM-2256R", 6120, 170);
R_Date("LTL-2659A", 6320, 50);
R_Date("LTL-2658A", 6385, 50);
R_Date("LTL-2657A", 6562, 50);
R_Date("UCLA-2148", 6700, 100);
R_Date("UB-2271", 6790, 255);
R_Date("LTL-1212A", 6542, 45);
R_Date("OxA-3683", 6200, 95);
R_Date("OxA-10013", 6450, 50);
R_Date("OxA-12064", 6501, 37);
R_Date("OxA-3685", 6510, 95);
R_Date("OxA-9989", 6510, 45);
R_Date("OxA-9990", 6555, 45);
R_Date("OxA-12063", 6601, 37);
R_Date("OxA-9988", 6605, 45);
R_Date("OxA-12062", 6638, 34);
R_Date("OxA-3684", 6640, 95);
R_Date("?", 6800, 80);
R_Date("MC-2292", 7124, 200);
R_Date("OxA-653", 6210, 70);
R_Date("OxA-652", 6280, 70);
R_Date("OxA-651", 6540, 80);
R_Date("LTL-1060A", 6352, 60);
R_Date("LTL-1062A", 6442, 50);
R_Date("-LTL 1061,00", 6600, 60);
R_Date("R-322", 6780, 90);
R_Date("LTL-5188A", 6561, 50);
R_Date("LTL-5187A", 6576, 45);
R_Date("R-846", 6140, 120);
R_Date("Beta 181785", 6340, 40);
R_Date("Beta-169498", 6340, 40);
R_Date("Beta-181784", 6410, 40);
R_Date("P-2946", 6930, 60);
R_Date("F-97", 6465, 185);
R_Date("LTL-427A", 6739, 60);
```

```
R_Date("LTL-141A", 6651, 50);
R_Date("LTL-3810A", 6764, 60);
R_Date("LTL-4536A", 6983, 50);
R_Date("LTL-142A", 7134, 60);
R_Date("LJ-150", 6530, 150);
R_Date("LJ-4550", 6530, 150);
R_Date("LJ-4549", 6760, 100);
R_Date("LJ-4551", 6900, 150);
R_Date("LJ-4548", 7110, 140);
R_Date("CAMS-2681", 6890, 60);
R_Date("LTL-16676A", 6910, 40);
R_Date("LTL-16677A", 6988, 45);
R_Date("LTL-1727A", 6277, 6277);
R_Date("AAR25751", 6072, 34);
R_Date("Gif-6722", 6530, 70);
R_Date("Gif-6724", 6980, 70);
R_Date("BM-2414", 6520, 70);
R_Date("LTL2154A", 6229, 55);
R_Date("LTL2156A", 6432, 60);
R_Date("Gif-7057", 6040, 70);
R_Date("Gif-7346", 6320, 80);
R_Date("Gif-7345", 6600, 120);
R_Date("Gif-7055", 6810, 80);
R_Date("R-351", 6540, 65);
R_Date("R-350", 7000, 100);
R_Date("LTL147A", 6410, 80);
R_Date("KIA10827", 6679, 54);
R_Date("LTL026A", 6762, 55);
R_Date("LTL221A", 6785, 40);
R_Date("Beta-59933", 6870, 70);
R_Date("Gif-6725", 6900, 80);
R_Date("Ly-4002", 6590, 140);
R_Date("LJ-1448", 6860, 45);
R_Date("TAN-88247", 6890, 130);
R_Date("TAN-88066", 6960, 130);
R_Date("X-53", 6960, 130);
R_Date("UGAMS-8552", 6060, 30);
R_Date("LTL-12139A", 6301, 45);
R_Date("LTL-12145A", 6612, 45);
R_Date("LTL-12146A", 6614, 45);
R_Date("LTL-12144A", 6632, 45);
R_Date("LTL-12142A", 6644, 45);
R_Date("LTL-12143A", 6683, 45);
R_Date("TAN-88068", 6660, 150);
R_Date("Ly-3948", 6710, 35);
R_Date("Lyon-4408", 6710, 180);
R_Date("Ly-3949", 6730, 40);
R_Date("Ly-3953", 6730, 40);
R_Date("Ly-3952", 6760, 40);
R_Date("TAN-88313", 6790, 120);
```

```
  R_Date("Ly-3950", 6810, 35);
  R_Date("Lyon-3896", 6810, 150);
  R_Date("Lyon-4410", 6830, 190);
  R_Date("Ly-3954", 6835, 40);
  R_Date("Ly-3951", 6840, 35);
  R_Date("Ly-3955", 6935, 40);
  R_Date("Lyon-5300", 6940, 270);
  R_Date("Lyon-4409", 6950, 190);
  R_Date("Lyon-5296", 6950, 150);
  R_Date("TAN-88056", 6950, 140);
  R_Date("TAN-88067", 6950, 130);
  R_Date("TAN-88248", 6980, 130);
  R_Date("Lyon-5297", 7030, 160);
  R_Date("Beta-169499", 6190, 40);
  R_Date("OxA-23122", 6432, 33);
  R_Date("OxA-23121", 6448, 30);
  R_Date("OxA-23119", 6452, 35);
  R_Date("OxA-23117", 6469, 32);
  R_Date("OxA-23118", 6484, 33);
  R_Date("OxA-23120", 6526, 34);
  R_Date("Beta-135147", 6620, 60);
  R_Date("Beta-122939", 6750, 50);
  R_Date("MC-2291", 6750, 220);
  R_Date("MC-2290", 6850, 130);
 };
 Boundary("Southern_Italy_neolithic_end");
 };
};
```

9. Supplementary Information – References

1. Broodbank, C. The origins and early development of Mediterranean maritime activity. *J. Mediterr. Archaeol.* **19**, 199–230 (2007).
2. Broodbank, C. *The Making of the Middle Sea: A History of the Mediterranean from the Beginning to the Emergence of the Classical World* (Thames & Hudson, 2013).
3. Leppard, T. P. Process and dynamics of Mediterranean neolithization (7000–5500 BC). *J. Archaeol. Res.* **30**, 231–283 (2022).
4. Dawson, H. *Mediterranean Voyages: The Archaeology of Island Colonisation and Abandonment* (Routledge, 2014).
5. Cherry, J. F. & Leppard, T. P. Patterning and its causation in the Pre-Neolithic colonization of the Mediterranean islands (Late Pleistocene to Early Holocene). *J. Isl. Coast. Archaeol.* **13**, 191–205 (2018).
6. Stiner, M. C. & Munro, N. D. On the evolution of diet and landscape during the Upper Paleolithic through Mesolithic at Franchthi Cave (Peloponnese, Greece). *J. Hum. Evol.* **60**, 618–636 (2011).
7. Starkovich, B. M., Munro, N. D. & Stiner, M. C. Terminal Pleistocene subsistence strategies and aquatic resource use in southern Greece. *Quat. Int.* **465**, 162–176 (2018).
8. Kelly, R. L. *The Lifeways of Hunter-Gatherers: The Foraging Spectrum* (Cambridge University Press, 2013).
9. Costa, L.-J. *et al.* Early settlement on Tyrrhenian islands (8th millennium cal. BC): Mesolithic adaptation to local resources in Corsica and Northern Sardinia in *Mesolithic on the Move* (eds. Larsson, L., Kindgren, H., Loeffler, D. & Åkerlund, A.) 3–10 (Oxbow, 2003).
10. Lo Vetro, D. & Martini, F. Il Paleolitico e il Mesolitico in Sicilia in *Atti XLI Riunione Scientifica IIPP, 'Dai Ciclopi agli Ecisti: Società e Territorio nella Sicilia Preistorica e Protostorica', San Cipirello (PA), 16-19 novembre 2006,* 19–48 (Istituto Italiano di Preistoria e Protostoria, 2012).
11. Lo Vetro, D. & Martini, F. Mesolithic in Central–Southern Italy: Overview of lithic productions. *Quat. Int.* **423**, 279–302 (2016).
12. Zazzo, A., Lebon, M., Quiles, A., Reiche, I. & Vigne, J.-D. Direct dating and physico-chemical analyses cast doubts on the coexistence of humans and dwarf hippos in Cyprus. *PLoS One* **10**, e0134429 (2015).
13. Tiné, V. & Tusa, S. Il Neolitico in Sicilia. in *Atti della XLI Riunione scientifica: 'Dai Ciclopi agli Ecisti: Società e Territorio nella Sicilia Preistorica e Protostorica', San Cipirello (PA), 16-19 Novembre 2006,* 49–80 (Istituto Italiano di Preistoria e Protostoria, 2012).
14. Parkinson, E. W., McLaughlin, T. R., Esposito, C., Stoddart, S. & Malone, C. Radiocarbon dated trends and Central Mediterranean Prehistory. *J. World Prehist.* **34**, 317–379 (2021).
15. Bradshaw, C. J. A. *et al.* Demographic models predict end-Pleistocene arrival and rapid expansion of pre-agropastoralist humans in Cyprus. *Proc. Natl. Acad. Sci. U.S.A.* **121**, (2024).
16. Heraclides, A. *et al.* Palaeogenomic insights into the origins of early settlers on the island of Cyprus. *Sci. Rep.* **14**, 9632 (2024).
17. Di Maida, G. The earliest human occupation of Sicily: A review. *J. Isl. Coast. Archaeol.* **17**, 402–419 (2022).
18. Antonioli, F. *et al.* Timing of the emergence of the Europe–Sicily bridge (40–17 cal ka BP) and its implications for the spread of modern humans in *Geology and Archaeology: Submerged Landscapes of the Continental Shelf* (eds. J. Harff; G. Bailey; F. Lüth) 111–144 (Geological Society of London, 2016).

19. Strasser, T. F. *et al.* Stone Age seafaring in the Mediterranean: Evidence from the Plakias Region for Lower Palaeolithic and Mesolithic habitation of Crete. *Hesperia* **79**, 145–190 (2010).
20. Runnels, C. *et al.* Lower Palaeolithic artifacts from Plakias, Crete: Implications for hominin dispersals. *Eurasian Prehist.* **11**, 129–152 (2014).
21. Sakellariou, D. & Galanidou, N. Agean Pleistocene landscapes above and below sea-level: Palaeogeographic reconstruction and hominin dispersals in *Under the Sea: Archaeology and Palaeolandscapes of the Continental Shelf* (eds. Bailey, G., Harff, J. & Sakellariou, D.) 335–259 (Springer, 2017).
22. Simmons, A. H. *Faunal Extinction in an Island Society: Pygmy Hippopotamus Hunters of Cyprus* (Plenum, 1999).
23. Caruso Fermé, L., Mineo, M., Remolins, G., Mazzucco, N. & Gibaja, J. F. Navigation during the early Neolithic in the Mediterranean area: study of wooden artifacts associated with dugout canoes at La marmotta (Lago di Bracciano, Anguillara Sabazia, Lazio, Italy). *Quat. Sci. Rev.* **311**, 108129 (2023).
24. Gibaja, J. F. *et al.* The first Neolithic boats in the Mediterranean: The settlement of La Marmotta (Anguillara Sabazia, Lazio, Italy). *PLoS One* **19**, e0299765 (2024).
25. Broodbank, C. *An Island Archaeology of the Early Cyclades* (Cambridge University Press, 2000).
26. Knapp, A. B. *Seafaring and Seafarer in the Bronze Age Eastern Mediterranean* (Sidestone Press, 2018).
27. Cherry, J. F. & Leppard, T. P. The Balearic paradox: Why were the islands colonized so late? *Pyrenae* **49**, 49–70 (2018).
28. Zilhão, J. The «African Mirage» is a delusion indeed. The distribution of the obsidian from Pantelleria rejects a Maghreb route for the neolithization of Iberia in *5.º Congresso do Neolítico Peninsular* (eds. Gonçalves, V. S., Diniz, M. & Sousa, A. C.) 623–630 (Centro de Arqueologia da Universidade de Lisboa, 2015).
29. Rotolo, S. G. *et al.* Obsidians of Pantelleria (Strait of Sicily): A petrographic, geochemical and magnetic study of known and new geological sources. *Open Archaeol.* **6**, 434–453 (2020).
30. Mulazzani, S. *et al.* Obsidian from the Epipalaeolithic and Neolithic eastern Maghreb. A view from the Hergla context (Tunisia). *J. Archaeol. Sci.* **37**, 2529–2537 (2010).
31. Zilhão, J. Early prehistoric navigation in the Western Mediterranean: Implications for the Neolithic transition in Iberia and the Maghreb. *Eurasian Prehist.* **11**, 185–200 (2014).
32. Broodbank, C. & Lucarini, G. Dynamics of Mediterranean Africa, ca. 9600–1000 bc. *J. Mediterr. Archaeol.* **32**, 195–267 (2019).
33. Lambeck, K., Rouby, H., Purcell, A., Sun, Y. & Sambridge, M. Sea level and global ice volumes from the Last Glacial Maximum to the Holocene. *Proc. Natl. Acad. Sci. U.S.A.* **111**, 15296–15303 (2014).
34. Perrin, T. The time of the last hunters: Chronocultural aspects of Early Holocene societies in the Western Mediterranean. *Open Archaeol.* **9**, 20220275 (2023).
35. O'Brien, M. J., Buchanan, B. & Eren, M. I. *Convergent Evolution in Stone-Tool Technology* (Massachusetts Institute of Technology Press, 2018).
36. Groucutt, H. S. *Culture History and Convergent Evolution: Can We Detect Populations in Prehistory?* (Springer, 2020).
37. Evans, J. *Prehistoric Antiquities of The Maltese Islands: A Survey* (Althone Press, 1971).
38. Trump, D. H. *Malta: Prehistory and Temples* (Midsea, 2002).
39. Malone, C. *et al. Temple Places: Excavating Cultural Sustainability in Prehistoric Malta* (McDonald Institute for Archaeological Research, 2020).
40. Zammit, T. *Prehistoric Malta: The Tarxien Temples* (Oxford University Press, 1930).

41. Sagona, C. *The Archaeology of Malta: From the Neolithic through the Roman Period* (Cambridge University Press, 2015).
42. Tanasi, D. & Vella, N. C. *The Late Prehistory of Malta: Essays on Borġ in-Nadur and Other Sites* (Archaeopress, 2015).
43. Keith, A. Discovery of Neanderthal man in Malta. *Nature* **101**, 404–405 (1918).
44. Keith, A. Neanderthal man in Malta. *J. R. Anthropol. Inst. Great Britain Ireland* **54**, 251–260 (1924).
45. Despott, G. Excavations at Għar Dalam (Dalam Cave), Malta. *J. R. Anthropol. Inst. Great Britain Ireland* **53**, 18–35 (1923).
46. Anati, E. Arte parietale a Malta: Relazione preliminare. *Bolletino del Centro Camuno di Study Prehistorici* **25**, 166–172 (1990).
47. Guagnin, M. *et al.* Evaluating possible prehistoric cave art in the central Mediterranean: Analyses of pigment traces and identification of taphonomic processes at Għar Ħasan, Malta. *J. Archaeol. Sci. Rep.* **47**, 103815 (2023).
48. McLaughlin, R., Parkinson, E. W., Reimer, P. J. & Malone, C. Dating Maltese prehistory in *Temple Places: Excavating Cultural Sustainability in Prehistoric Malta* (eds. Malone, C. et al.) 27–38 (McDonald Institute for Archaeological Research, 2020).
49. Marriner, N. *et al.* Fire as a motor of rapid environmental degradation during the earliest peopling of Malta 7500 years ago. *Quat. Sci. Rev.* **212**, 199–205 (2019).
50. Hunt, C. O. *et al.* Chronology and stratigraphy of the valley systems in *Temple Landscapes: Fragility, Change and Resilience of Holocene Environments in the Maltese Islands* (eds. French, C. et al.) 35–72 (McDonald Institute for Archaeological Research, 2020).
51. Farrell, M., Hunt, C. O. & McClung, L. C. The Holocene vegetation history of the Maltese Islands in *Temple Landscapes: Fragility, Change and Resilience of Holocene Environments in the Maltese Islands* (eds. French, C. et al.) 73–114 (McDonald Institute for Archaeological Research, 2020).
52. Parnell, A. C., Haslett, J., Allen, J. R. M., Buck, C. E. & Huntley, B. A flexible approach to assessing synchroneity of past events using Bayesian reconstructions of sedimentation history. *Quat. Sci. Rev.* **27**, 1872–1885 (2008).
53. Carroll, F. A., Hunt, C. O., Schembri, P. J. & Bonanno, A. Holocene climate change, vegetation history and human impact in the Central Mediterranean: Evidence from the Maltese Islands. *Quat. Sci. Rev.* **52**, 24–40 (2012).
54. Fenech, K., Hunt, C. O., Vella, N. C. & Schembri, P. J. Molluscan remains from the valley cores in *Temple Landscapes: Fragility, Change and Resilience of Holocene Environments in the Maltese Islands* (eds. French, C. et al.) 115–160 (McDonald Institute for Archaeological Research, 2020).
55. Blaauw, M. Methods and code for 'classical' age-modelling of radiocarbon sequences. *Quat. Geochronol.* **5**, 512–518 (2010).
56. Trachsel, M. & Telford, R. J. All age-depth models are wrong, but are getting better. *Holocene* **27**, 860–869 (2017).
57. Bronk Ramsey, C. Bayesian analysis of radiocarbon dates. *Radiocarbon* **51**, 337–360 (2009).
58. Reimer, P. J. *et al.* The IntCal20 northern hemisphere radiocarbon age calibration curve (0–55 cal kBP). *Radiocarbon* **62**, 725–757 (2020).
59. Lugliè, C. *Your path led trough the sea …* The emergence of Neolithic in Sardinia and Corsica. *Quat. Int.* **470**, 285–300 (2018).
60. Skeates, R. *An Archaeology of the Senses: Prehistoric Malta* (Oxford University Press, 2010).

61. Dawson, H. Understanding colonisation: Adaptation strategies in the central Mediterranean Islands. *Accordia Res. Papers* **10**, 35–60 (2006).
62. Mussi, M. & Melis, R. T. Santa Maria is Acquas e le problematiche del Paleolitico Superiore in Sardegna in *World Islands in Prehistory: International Insular Investigations* (eds. Waldren, W. H. & Ensenyat, J. A.) 67–94 (Archaeopress, 2002).
63. Sondaar, P. *et al.* The human colonization of Sardinia: A Late-Pleistocene human fossil from Corbeddu Cave. *C. R. Acad. Sci.* **320**, 145–150 (1995).
64. Dawson, H. Island colonisation and abandonment in Mediterranean Prehistory (University College London, 2005).
65. Vigne, J. D. The origins of mammals on the Mediterranean islands as indicator of early voyaging. *Eurasian Prehist.* **10**, 45–56 (2014).
66. Palombo, M. R. *et al.* The late Pleistocene to Holocene palaeogeographic evolution of the Porto Conte area: Clues for a better understanding of human colonization of Sardinia and faunal dynamics during the last 30 ka. *Quat. Int.* **439**, 117–140 (2017).
67. Cristiani, E., Melis, R. T. & Mussi, M. Marine shells as grave goods at S'Omu e S'Orku (Sardinia, Italy) in *Foraging Assemblages* (eds. Borić, D., Antonović, D. & Mihailović, B.) 558–566 (Serbian Archaeological Society, 2021).
68. Costa, L. J. Nouvelles données sur le Mésolithique des îles Tyrrhéniennes (Corse et Sardaigne) [Peut-on parler d'un « Mésolithique insulaire » ?]. *Gallia Préhistoire* **46**, 211–230 (2004).
69. Bianchini, G. Risultati delle ricerche sul Paleolitico inferiore in Sicilia e la scoperta di industrie del gruppo "Pebble Culture" nei terrazzi quaternari di Capo Rossello in territorio di Realmonte in *Atti della XIII riunione scientifica dell'Istituto Italiano di Preistoria e Protostoria, Siracusa–Malta 1968,* 89–109 (Istituto Italiano di Preistoria e Protostoria, 1971).
70. Leighton, R. *Sicily Before History: An Archaeological Survey from the Palaeolithic to the Iron Age* (Cornell University Press, 1999).
71. Chilardi, S., Frayer, D. W., Gioia, P., Macchiarelli, R. & Mussi, M. Fontana Nuova di Ragusa (Sicily, Italy): Southernmost Aurignacian site in Europe. *Antiquity* **70**, 553–563 (1996).
72. Di Maida, G., Mannino, M. A., Krause-Kyora, B., Jensen, T. Z. T. & Talamo, S. Radiocarbon dating and isotope analysis on the purported Aurignacian skeletal remains from Fontana Nuova (Ragusa, Italy). *PLoS One* **14**, e0213173 (2019).
73. Martini, F. *et al.* L'Epigravettiano finale in Sicilia in *L' Italia tra 15.000 e 10.000 Anni fa. Cosmopolitismo e Regionalità nel Tardoglaciale* (ed. Martini, F.) 209–254 (Museo Fiorentino di Preistoria «Paolo Graziosi», 2007).
74. Mannino, M. A. *et al.* Upper Palaeolithic hunter-gatherer subsistence in Mediterranean coastal environments: An isotopic study of the diets of the earliest directly-dated humans from Sicily. *J. Archaeol. Sci.* **38**, 3094–3100 (2011).
75. Nicoletti, F. & Tusa, S. Nuove acquisizioni scientifiche sul Riparo del Castello di Termini Imerese (Palermo) nel quadro della preistoria siciliana tra la fine del Pleistocene e gli inizi dell'Olocene in *Atti della XLI Riunione scientifica, 'Dai Ciclopi agli Ecisti. Società e Territorio nella Sicilia Preistorica e Protostorica, San Cipirello (PA), 16-19 Novembre 2006,* 303–318 (Istituto Italiano di Preistoria e Protostoria, 2012).
76. Garilli, V. *et al.* First evidence of Pleistocene ochre production from bacteriogenic iron oxides. A case study of the Upper Palaeolithic site at the San Teodoro Cave (Sicily, Italy). *J. Archaeol. Sci.* **123**, 105221 (2020).
77. Mannino, M. A. *et al.* Climate-driven environmental changes around 8,200 years ago favoured increases in cetacean strandings and Mediterranean hunter-gatherers exploited them. *Sci. Rep.* **5**, 16288 (2015).

78. Tagliacozzo, A. Economic changes between the Mesolithic and Neolithic in the Grotta dell'Uzzo (Sicily, Italy). *Accordia Res. Papers* **5**, 7–37 (1993).
79. Compagnoni, B. La malcofauna del sito meso-neolitico della Grotta dell'Uzzo (Trapani). *Rivista di Scienze Preistoriche* **43**, 49–72 (1993).
80. Mannino, M. *et al.* Marine resources in the Mesolithic and Neolithic at the Grotta dell'Uzzo (Sicily): Evidence from isotope analyses of marine shells. *Archaeometry* **49**, 117–133 (2007).
81. Colonese, A. C. *et al.* Late Pleistocene-Holocene coastal adaptation in central Mediterranean: Snapshots from Grotta d'Oriente (NW Sicily). *Quat. Int.* **493**, 114–126 (2018).
82. Mannino, M. A. & Thomas, K. D. Current research on prehistoric human coastal ecology: Late Pleistocene and Early Holocene hunter-gatherer transitions in north-west Sicily in *Mesolithic Horizons* (eds. McCartan, S., Woodman, P., Schulting, R. & Warren, G.) 140–145 (Oxbow, Oxford, 2009).
83. Mannino, M. A. *et al.* Origin and diet of the prehistoric hunter-gatherers on the Mediterranean island of Favignana (Ègadi Islands, Sicily). *PLoS One* **7**, e49802 (2012).
84. Yu, H. *et al.* Genomic and dietary discontinuities during the Mesolithic and Neolithic in Sicily. *iScience* **25**, 104244 (2022).
85. Ammerman, A. & Cavalli-Sforza, L. The wave of advance model for the spread of agriculture in Europe in *Transformations. Mathematical Approaches to Culture Change* (eds. Renfrew, C., Cooke, K. L.) 275–293 (Elsevier, 1979).
86. Ammerman, A. J. & Cavalli-sforza, L. L. *The Neolithic Transition and the Genetics of Populations in Europe* (Princeton University Press, 1984).
87. Bogucki, P. Neolithic dispersals in riverine interior Central Europe in *The Widening Harvest. The Neolithic Transition in Europe: Looking Back, Looking Forward* (eds. Ammerman, A. J. & Biagi, P.) 249–272 (Archaeological Institute of America, 2003).
88. Bernabeu Aubán, J., García-Puchol, O. & Orozco-Köhler, T. New insigths relating to the beginning of the Neolithic in the eastern Spain: Evaluating empirical data and modelled predictions. *Quat. Int.* **470**, 439–450 (2018).
89. Isern, N., Zilhão, J., Fort, J. & Ammerman, A. J. Modeling the role of voyaging in the coastal spread of the Early Neolithic in the West Mediterranean. *Proc. Natl. Acad. Sci. U.S.A.* **114**, 897–902 (2017).
90. Bogaard, A. *Neolithic Farming in Central Europe. An Archaebotanical Study of Crop Husbandry Practices* (Routledge, 2004).
91. Robb, J. & Miracle, P. Beyond 'migration' versus 'acculturation': New models for the spread of agriculture. *Proc. Br. Acad.* **144**, 99–115 (2007).
92. Zeder, M. A. Domestication and early agriculture in the Mediterranean Basin: Origins, diffusion, and impact. *Proc. Natl. Acad. Sci. U.S.A.* **105**, 11597–11604 (2008).
93. Robb, J. Material culture, landscapes of action, and emergent causation. *Curr. Anthropol.* **54**, 657–683 (2013).
94. García-Puchol, O. & Salazar-García, D. C. *Times of Neolithic Transition along the Western Mediterranean* (Springer, 2017).
95. Shennan, S. *The First Farmers of Europe: An Evolutionary Perspective* (Cambridge University Press, 2018).
96. Catapoti, M. & Relakki, D. Why the Neolithic is (r)evolutionary. *J. Material Cult.* **25**, 289–308 (2020).
97. Barrett, J. *Archaeology and Its Discontents: Why Archaeology Matters?* (Routledge, 2021).
98. Graeber, D. & Wengrow, D. *The Dawn of Everything. A New History of Humanity* (Penguin, 2021).

99. Mazzucco, N. *et al.* Migration, adaptation, innovation: The spread of Neolithic harvesting technologies in the Mediterranean. *PLoS One* **15**, e0232455 (2020).
100. Zvelebil, M. & Rowley-Conwy, P. Transition to farming in Northern Europe: A hunter-gatherer perspective. *Nor. Archaeol. Rev.* **17**, 104–128 (1984).
101. Rowley-Conwy, P. How the west was lost. *Curr. Anthropol.* **45**, S83–S113 (2004).
102. Bramanti, B. *et al.* Genetic discontinuity between local hunter-gatherers and central Europe's first farmers. *Science* **326**, 137–140 (2009).
103. Lipson, M. *et al.* Parallel palaeogenomic transects reveal complex genetic history of early European farmers. *Nature* **551**, 368–372 (2017).
104. Mathieson, I. *et al.* The genomic history of southeastern Europe. *Nature* **555**, 197–203 (2018).
105. Brami, M. *et al.* Was the fishing village of Lepenski Vir built by Europe's first farmers? *J. World Prehist.* **35**, 109–133 (2022).
106. Betti, L. *et al.* Climate shaped how Neolithic farmers and European hunter-gatherers interacted after a major slowdown from 6,100 BCE to 4,500 BCE. *Nat. Hum. Behav.* **4**, 1004–1010 (2020).
107. Fort, J. Dispersal distances and cultural effects in the spread of the Neolithic along the northern Mediterranean coast. *Archaeol. Anthropol. Sci.* **14**, 153 (2022).
108. Palmisano, A., Bevan, A., Kabelindde, A., Roberts, N. & Shennan, S. Long-term demographic trends in prehistoric Italy: Climate impacts and regionalised socio-ecological trajectories. *J. World Prehist.* **34**, 381–432 (2021).
109. Dyson, S. L. & Rowland, R. J. *Archaeology and History in Sardinia from the Stone Age to the Middle Ages: Shepherds, Sailors, and Conquerors* (University of Pennsylvania Press, 2007).
110. Marcus, J. H. *et al.* Genetic history from the Middle Neolithic to present on the Mediterranean island of Sardinia. *Nat. Commun.* **11**, 939 (2020).
111. Trump, D. H. La grotta di Filiestru a Bonu Ighinu, Mara (SS). *Quaderni della Soprintendenza ai Beni Archeologici per le Provincie di Sassari e Nuoro* **13**, 1–108 (1983).
112. Webster, G. *The Sardinian Neolithic: An Archaeology of the 6th and 5th Millennia BCE* (British Archaeological Reports Publishing, 2019).
113. Tiné, V. The stratigraphic sequence of the Grotta del Kronio and the neolithization of Sicily and Calabria A brief chrono-cultural summary in *From Cave to Dolmen: Ritual and Symbolic Aspects in the Prehistory between Sciacca, Sicily and the Central Mediterranean* (ed. Gullì, D.) 43–50 (Archaeopress, 2014).
114. Zilhão, J. Radiocarbon evidence for maritime pioneer colonization at the origins of farming in west Mediterranean Europe. *Proc. Natl. Acad. Sci. U.S.A.* **98**, 14180–14185 (2001).
115. Juan-Cabanilles, J. & Martí Oliver, B. New approaches to the Neolithic transition: The last hunters and first farmers of the western Mediterranean in *Times of Neolithic Transition along the Western Mediterranean* (eds. García-Puchol, O. & Salazar-García, D.) 33–65 (Springer, 2017).
116. Bailey, G. & Spikins, P. *Mesolithic Europe* (Cambridge University Press, 2008).
117. Alessio, M., Bella, F., Bachechi, F. & Cortesi, C. University of Rome Carbon-14 Dates V. *Radiocarbon* **9**, 346–367 (1967).
118. Tagliacozzo, A., Fiore, I., Lo Vetro, D., Calcagnile, C. & Tiné, V. The Mesolithic levels of Grotta del Santuario della Madonna at Praia a Mare (Cosenza, Italy): New excavations, chronological data and techno-typological features of the lithic assemblages. *Preistoria Alpina* **48**, 183–197 (2016).

119. Rolfo, M. F., Achino, K. F., Fusco, I., Salari, L. & Silvestri, L. Reassessing human occupation patterns in the inner central Apennines in prehistory: The case-study of Grotta Mora Cavorso. *J. Archaeol. Sci. Rep.* **7**, 358–367 (2016).
120. Whitehouse, R. D. The chronology of the Neolithic ditched settlements of the Tavoliere and the Ofanto valley. *Accordia Res. Papers* **13**, 57–78 (2013).
121. Skeates, R. A radiocarbon date-list for prehistoric Italy (c. 46,400 BP – 2450 BP/400 cal. BC in *Radiocarbon dating and Italian Prehistory* (eds. Skeates, R. & Whitehouse, R.) 147–288 (British School at Rome, 1994).
122. Binder, D. *et al.* Modelling the earliest north-western dispersal of Mediterranean impressed wares: New dates and Bayesian chronological model. *Documenta Praehistorica* **44**, 54–77 (2017).
123. Natali, E. & Forgia, V. The beginning of the Neolithic in southern Italy and Sicily. *Quat. Int.* **470**, 253–269 (2018).
124. Speciale, C. Sicily and the process of Neolithisation: A review of the archaeobotanical data. *Veg. Hist. Archaeobot.* **33**, 185–194 (2024).
125. Collina, C. Sistemi tecnici e chaînes opératoires alla grotta dell'Uzzo (Trapani): analisi tecnologica delle industrie litiche dai livelli mesolitici e neolitici in *Atti della XLI Riunione scientifica, 'Dai Ciclopi agli Ecisti : Società e Territorio nella Sicilia Preistorica e Protostorica, San Cipirello (PA), 16-19 Novembre 2006,* 447–459 (Istituto Italiano di Preistoria e Protostoria, 2012).
126. Balco, W. M. Neolithic cultural hybridity: Social entanglements and the development of hybrid culture in the western Mediterranean. *Field Notes: J. Collegiate Anthropol.* **1**, 1 (2009).
127. Fernandes, D. M. *et al.* The spread of steppe and Iranian-related ancestry in the islands of the western Mediterranean. *Nat. Ecol. Evol.* **4**, 334–345 (2020).
128. Pearce, M. *Rethinking the North Italian Early Neolithic* (Accordia Research Institute, 2013).
129. Gazzoni, V. *et al.* Investigating the diet of Mesolithic groups in the Southern Alps: An attempt using stable carbon and nitrogen isotope analyses. *Bull. Mem. Soc. Anthropol. Paris* **33**, (2021).
130. van der Geer. A., Lyras, G., de Vos, J. & Dermitzakis, M. *Evolution of Island Mammals. Adaptation and Extinction of Placental Mammals on Islands* (Wiley-Blackwell, 2010).
131. Muhs, D. R., Budahn, J., Avila, A., Skipp, G., Freeman, J. & Patterson, D. The role of African dust in the formation of Quaternary soils on Mallorca, Spain and implications for the genesis of Red Mediterranean soils. *Quat. Sci. Rev.* **29**, 2518–2543 (2010).
132. Mentzer, S. M. Microarchaeological approaches to the identification and interpretation of combustion features in prehistoric archaeological sites. *J. Archaeol. Method Theory* **21**, 616–668 (2014).
133. Canti, M. G. & Linford, N. The effects of fire on archaeological soils and sediments: Temperature and colour relationships. *Proc. Prehist. Soc.* **66**, 385–395 (2000).
134. Karkanas, P. *et al.* Evidence for habitual use of fire at the end of the Lower Paleolithic: Site-formation processes at Qesem Cave, Israel. *J. Hum. Evol.* **53**, 197–212 (2007).
135. Aldeias, V. Experimental approaches to archaeological fire features and their behavioral relevance. *Curr. Anthropol.* **58**, S191–S205 (2017).
136. Canti, M. G. Burnt carbonates in *Archaeological Soil and Sediment Micromorphology* (eds. Nicosia, C. & Stoops, G.) 181–188 (Wiley-Blackwell, 2017).
137. Duarte, C., Iriarte, E., Diniz, M. & Arias, P. The microstratigraphic record of human activities and formation processes at the Mesolithic shell midden of Poças de São Bento (Sado Valley, Portugal). *Archaeol. Anthropol. Sci.* **11**, 483–509 (2019).

138. Villagran, X. S. The shell midden conundrum: Comparative micromorphology of shell-matrix sites from South America. *J. Archaeol. Method Theory* **26**, 344–395 (2019).
139. McAdams, C. *et al.* Late Pleistocene shell midden microstratigraphy indicates a complex history of human–environment interactions in the uplands of northern Vietnam. *Phil. Trans. R. Soc. B* **377**, 20200493 (2022).
140. Yaalon, D. H. Soils in the Mediterranean region: What makes them different? *Catena* **28**, 157–169 (1997).
141. Bunting, M. J. & Tipping, R. Sorting dross from data: Possible indicators of post-depositional assemblage biasing in archaeological palynology in *Human Ecodynamics* (eds. Bailey, G., Winder, N. & Charles, R.) 63–69 (Oxbow, 2000).
142. Djamali, M. *et al.* Vegetation dynamics during the early to mid-Holocene transition in NW Malta, human impact versus climatic forcing. *Veg. Hist. Archaeobot.* **22**, 367–380 (2013).
143. Médail, F. Plant biogeography and vegetation patterns of the Mediterranean islands. *Bot. Rev.* **88**, 63–129 (2022).
144. Brullo, S., Brullo, C., Cambria, S. & del Galdo, G. G. *The Vegetation of the Maltese Islands* (Springer, 2020).
145. Hunt, C. O. & Fiacconi, M. Pollen taphonomy of cave sediments: What does the pollen record in caves tell us about external environments and how do we assess its reliability? *Quat. Int.* **485**, 68–75 (2018).
146. Dimbleby, G. W. *The Palynology of Archaeological Sites* (Academic Press, 1985).
147. Hunt, C. O. & Rushworth, G. Pollen taphonomy and airfall sedimentation in a tropical cave: The West Mouth of The Great Cave of Niah in Sarawak, Malaysian Borneo. *J. Archaeol. Sci.* **32**, 465–473 (2005).
148. Navarro-Camacho, C., Carrión, J. S., Navarro, J., Munuera, M. & Prieto, A. R. An experimental approach to the palynology of cave deposits. *J. Quat. Sci.* **15**, 603–619 (2000).
149. Carrión, J. S. *et al.* Quaternary pollen analysis in the Iberian Peninsula: The value of negative results. *Internet Archaeol.* **25** (2009).
150. Florenzano, A., Marignani, M., Rosati, L., Fascetti, S. & Mercuri, A. M. Are Cichorieae an indicator of open habitats and pastoralism in current and past vegetation studies? *Plant Biosyst.* **149**, 154–165 (2015).
151. Fiacconi, M. & Hunt, C. O. Palynology of surface sediments from caves in the Zagros Mountains (Kurdish Iraq): Patterns and processes. *Rev. Palaeobot. Palynol.* **239**, 66–76 (2017).
152. Gambin, B. *et al.* 7300 years of vegetation history and climate for NW Malta: A Holocene perspective. *Clim. Past* **12**, 273–297 (2016).
153. Caló, C. *et al.* Spatio-temporal patterns of Holocene environmental change in southern Sicily. *Palaeogeogr. Palaeoclimatol. Palaeoecol.* **323–325**, 110–122 (2012).
154. Noti, R. *et al.* Mid- and late-Holocene vegetation and fire history at Biviere di Gela, a coastal lake in southern Sicily, Italy. *Veg. Hist. Archaeobot.* **18**, 371–387 (2009).
155. Tinner, W. *et al.* Holocene environmental and climatic changes at Gorgo Basso, a coastal lake in southern Sicily, Italy. *Quat. Sci. Rev.* **28**, 1498–1510 (2009).
156. Desprat, S. *et al.* Deglacial and Holocene vegetation and climatic changes in the southern Central Mediterranean from a direct land–sea correlation. *Clim. Past* **9**, 767–787 (2013).
157. Carrión Marco, Y. *et al.* Late Glacial landscape dynamics based on macrobotanical data: Evidence from Ifri El Baroud (NE Morocco). *Environ. Archaeol.* **26**, 131–145 (2021).
158. Carrión Marco, Y., Pérez Jordà, G., Kherbouche, F. & Peña-Chocarro, L. Plant use and vegetation trends in Algeria from Late Glacial to Middle Holocene: Charcoal and seeds from Gueldaman GLD 1 cave (Babors d'Akbou). *Rev. Palaeobot. Palynol.* **297**, 104562 (2022).

159. Picornell-Gelabert, L. & Carrión Marco, Y. Landscape and firewood procurement at the prehistoric and protohistoric site of Ses Païsses (island of Mallorca, Western Mediterranean). *Quat. Int.* **458**, 56–74 (2017).
160. Rodríguez-Ariza, M. O. *et al.* Long-term human impact and forest management in the Phoenician and Roman city of Utica (Tunisia) (900 BC–500 AD). *Holocene* **31**, 943–953 (2021).
161. Mallol, C., Marlowe, F. W., Wood, B. M. & Porter, C. C. Earth, wind, and fire: Ethnoarchaeological signals of Hadza fires. *J. Archaeol. Sci.* **34**, 2035–2052 (2007).
162. Berna, F. & Goldberg, P. Assessing Paleolithic pyrotechnology and associated hominin behavior in Israel. *Isr. J. Earth Sci.* **56**, 107–121 (2007).
163. Goldberg, P. *et al.* New evidence on Neandertal use of fire: Examples from Roc de Marsal and Pech de l'Azé IV. *Quat. Int.* **247**, 325–340 (2012).
164. Gabucio, M. J. *et al.* Unraveling a Neanderthal palimpsest from a zooarcheological and taphonomic perspective. *Archaeol. Anthropol. Sci.* **10**, 197–222 (2018).
165. Henry, D. The palimpsest problem, hearth pattern analysis, and Middle Paleolithic site structure. *Quat. Int.* **247**, 246–266 (2012).
166. Mora Torcal, R., Roy Sunyer, M., Martínez-Moreno, J., Benito-Calvo, A. & Samper Carro, S. Inside the palimpsest: Identifying short occupations in the 497D Level of Cova Gran (Iberia) in *Short-Term Occupations in Paleolithic Archaeology* (eds. Cascalheira, J. & Picin, A.) 39–69 (Springer, 2020).
167. Vallverdú, J. *et al.* Combustion structures of archaeological level O and Mousterian activity areas with use of fire at the Abric Romaní rockshelter (NE Iberian Peninsula). *Quat. Int.* **247**, 313–324 (2012).
168. Barkai, R., Rosell, J., Blasco, R. & Gopher, A. Fire for a Reason: Barbecue at Middle Pleistocene Qesem Cave, Israel. *Curr. Anthropol.* **58**, S314–S328 (2017).
169. Carmody, R. N. & Wrangham, R. W. The energetic significance of cooking. *J. Hum. Evol.* **57**, 379–391 (2009).
170. Rosell, J. & Blasco, R. The early use of fire among Neanderthals from a zooarchaeological perspective. *Quat. Sci. Rev.* **217**, 268–283 (2019).
171. Aranguren, B. *et al.* Wooden tools and fire technology in the early Neanderthal site of Poggetti Vecchi (Italy). *Proc. Natl. Acad. Sci. U.S.A.* **115**, 2054–2059 (2018).
172. Leder, D. *et al.* The wooden artifacts from Schöningen's Spear Horizon and their place in human evolution. *Proc. Natl. Acad. Sci. U.S.A.* **121**, (2024).
173. Niekus, M. J. L. Th. *et al.* Middle Paleolithic complex technology and a Neandertal tar-backed tool from the Dutch North Sea. *Proc. Natl. Acad. Sci. U.S.A.* **116**, 22081–22087 (2019).
174. Attwell, L., Kovarovic, K. & Kendal, J. Fire in the Plio-Pleistocene: The functions of hominin fire use, and the mechanistic, developmental and evolutionary consequences. *J. Anthropol. Sci.* **93**, 1–20 (2015).
175. Jaubert, J. *et al.* Early Neanderthal constructions deep in Bruniquel Cave in southwestern France. *Nature* **534**, 111–114 (2016).
176. Wiessner, P. W. Embers of society: Firelight talk among the Ju/'hoansi Bushmen. *Proc. Natl. Acad. Sci. U.S.A.* **111**, 14027–14035 (2014).
177. Weiner, S. *Microarchaeology: Beyond the Visible Archaeological Record* (Cambridge University Press, 2010).
178. Allué, E. *et al.* Fire among Neanderthals in *Updating Neanderthals. Understanding Behavioural Complexity in the Late Middle Palaeolithic* (eds. Romagnoli, F., Rivals, F. & Bennazzi, S.) 227–249 (Elsevier, 2022).
179. Murphree, W. C. & Aldeias, V. The evolution of pyrotechnology in the Upper Palaeolithic of Europe. *Archaeol. Anthropol. Sci.* **14**, 202 (2022).

180. Berna, F. *et al.* Sediments exposed to high temperatures: Reconstructing pyrotechnological processes in Late Bronze and Iron Age Strata at Tel Dor (Israel). *J. Archaeol. Sci.* **34**, 358–373 (2007).
181. Hlubik, S., Berna, F., Feibel, C., Braun, D. & Harris, J. W. K. Researching the nature of fire at 1.5 Mya on the site of FxJj20 AB, Koobi Fora, Kenya, using high-resolution spatial analysis and FTIR spectrometry. *Curr. Anthropol.* **58**, S243–S257 (2017).
182. Karkanas, P. *et al.* The earliest evidence for clay hearths: Aurignacian features in Klisoura Cave 1, southern Greece. *Antiquity* **78**, 513–525 (2004).
183. Allué, E., Solé, A. & Burguet-Coca, A. Fuel exploitation among Neanderthals based on the anthracological record from Abric Romaní (Capellades, NE Spain). *Quat. Int.* **431**, 6–15 (2017).
184. Madella, M., Jones, M. K., Goldberg, P., Goren, Y. & Hovers, E. The exploitation of plant resources by Neanderthals in Amud Cave (Israel): The evidence from phytolith studies. *J. Archaeol. Sci.* **29**, 703–719 (2002).
185. Marquer, L., Otto, T., Nespoulet, R. & Chiotti, L. A new approach to study the fuel used in hearths by hunter-gatherers at the Upper Palaeolithic site of Abri Pataud (Dordogne, France). *J. Archaeol. Sci.* **37**, 2735–2746 (2010).
186. Wroth, K. *et al.* Neanderthal plant use and pyrotechnology: Phytolith analysis from Roc de Marsal, France. *Archaeol. Anthropol. Sci.* **11**, 4325–4346 (2019).
187. Brittingham, A. *et al.* Geochemical evidence for the control of fire by Middle Palaeolithic hominins. *Sci. Rep.* **9**, 15368 (2019).
188. Jambrina-Enríquez, M. *et al.* n-Alkyl nitriles and compound-specific carbon isotope analysis of lipid combustion residues from Neanderthal and experimental hearths: Identifying sources of organic compounds and combustion temperatures. *Quat. Sci. Rev.* **222**, 105899 (2019).
189. Carrancho, Á., Herrejón Lagunilla, Á. & Vergès, J. M. Three archaeomagnetic applications of archaeological interest to the study of burnt anthropogenic cave sediments. *Quat. Int.* **414**, 244–257 (2016).
190. Carrancho, Á., Villalaín, J. J., Vallverdú, J. & Carbonell, E. Is it possible to identify temporal differences among combustion features in Middle Palaeolithic palimpsests? The archaeomagnetic evidence: A case study from level O at the Abric Romaní rock-shelter (Capellades, Spain). *Quat. Int.* **417**, 39–50 (2016).
191. Albert, R. M. *et al.* Mode of occupation of Tabun Cave, Mt Carmel, Israel during the Mousterian period: A study of the sediments and phytoliths. *J. Archaeol. Sci.* **26**, 1249–1260 (1999).
192. Albert, R. M., Weiner, S., Bar-Yosef, O. & Meignen, L. Phytoliths in the Middle Palaeolithic deposits of Kebara Cave, Mt Carmel, Israel: Study of the plant materials used for fuel and other purposes. *J. Archaeol. Sci.* **27**, 931–947 (2000).
193. Albert, R. M. & Marean, C. W. The exploitation of plant resources by early *Homo sapiens*: The phytolith record from Pinnacle Point 13B Cave, South Africa. *Geoarchaeology* **27**, 363–384 (2012).
194. Esteban, I., Albert, R. M., Eixea, A., Zilhão, J. & Villaverde, V. Neanderthal use of plants and past vegetation reconstruction at the Middle Paleolithic site of Abrigo de la Quebrada (Chelva, Valencia, Spain). *Archaeol. Anthropol. Sci.* **9**, 265–278 (2017).
195. Esteban, I. *et al.* Plants, people and fire: Phytolith and FTIR analyses of the post-Howiesons Poort occupations at Border Cave (KwaZulu-Natal, South Africa). *Quat. Sci. Rev.* **300**, 107898 (2023).
196. Karkanas, P., Rigaud, J.-P., Simek, J. F., Albert, R. M. & Weiner, S. Ash, bones and Guano: A study of the minerals and phytoliths in the sediments of Grotte XVI, Dordogne, France. *J. Archaeol. Sci.* **29**, 721–732 (2002).

197. Rodríguez-Cintas, Á. & Cabanes, D. Phytolith and FTIR studies applied to combustion structures: The case of the Middle Paleolithic site of El Salt (Alcoy, Alicante). *Quat. Int.* **431**, 16–26 (2017).
198. Shahack-Gross, R. *et al.* Evidence for the repeated use of a central hearth at Middle Pleistocene (300 ky ago) Qesem Cave, Israel. *J. Archaeol. Sci.* **44**, 12–21 (2014).
199. Neumann, K. *et al.* International code for phytolith nomenclature (ICPN) 2.0. *Ann. Bot.* **124**, 189–199 (2019).
200. García-Granero, J. J. *et al.* A long-term assessment of the use of *Phoenix theophrasti* Greuter (Cretan date palm): The ethnobotany and archaeobotany of a neglected palm. *J. Ethnobiol.* **40**, 101 (2020).
201. Madella, M., Alexandre, A. & Ball, T. International code for phytolith nomenclature 1.0. *Ann. Bot.* **96**, 253–260 (2005).
202. Piperno, D. R. *Phytoliths: A Comprehensive Guide for Archaeologists and Paleoecologists*. (AltaMira Press, 2006).
203. Albert, R. M., Bamford, M. K. & Cabanes, D. Palaeoecological significance of palms at Olduvai Gorge, Tanzania, based on phytolith remains. *Quat. Int.* **193**, 41–48 (2009).
204. Benvenuto, M. L., Fernández Honaine, M., Osterrieth, M. L. & Morel, E. Differentiation of globular phytoliths in Arecaceae and other monocotyledons: Morphological description for paleobotanical application. *Turk. J. Bot.* **39**, 341–353 (2015).
205. Giovino, A. *et al.* Genetic variability of *Chamaerops humilis* (Arecaceae) throughout its native range highlights two species movement pathways from its area of origin. *Bot. J. Linn. Soc.* **201**, 361–376 (2023).
206. Mifsud, S. Palms on the Maltese lslands. *Principes* **39**, 190–196 (1995).
207. Stevens, D. T., Lanfranco, E., Mallia, A. & Schembri, P. J. Biodiversity conservation and utilisation in the Maltese Islands in *Identifying and Monitoring Biodiversity and its Utilization in Commonwealth Small Island Developing States,* 1–33 (Commonwealth Science Council, 1995).
208. Mifsud, S. Online Flora of the Maltese Islands, https://www.maltawildplants.com/wildplants_index.php (2024).
209. Hansen, J. & Renfrew, J. M. Palaeolithic–Neolithic seed remains at Franchthi Cave, Greece. *Nature* **271**, 349–352 (1978).
210. Zohary, D. The wild progenitor and the place of origin of the cultivated lentil: *Lens culinaris*. *Econ. Bot.* **26**, 326–332 (1972).
211. Zvelebil, M. Plant use in the Mesolithic and its role in the transition to farming. *Proc. Prehist. Soc.* **60**, 35–74 (1994).
212. Siani, G. *et al.* Radiocarbon reservoir ages in the Mediterranean Sea and Black Sea. *Radiocarbon* **42**, 271–280 (2000).
213. Lindauer, S., Milano, S., Steinhof, A. & Hinderer, M. Heating mollusc shells - A radiocarbon and microstructure perspective from archaeological shells recovered from Kalba, Sharjah Emirate, UAE. *J. Archaeol. Sci. Rep.* **21**, 528–537 (2018).
214. Groucutt, H. S. Maltese chert: An archaeological perspective on raw material and lithic technology in the central Mediterranean. *Malta Archaeol. Rev.* 1–20 (2022).
215. D'Souza, L. C. An endemic radiation of deer in the Late Pleistocene of Malta (University of Bristol, 2019).
216. Forgia, V. *et al.* New Data on Sicilian prehistoric and historic evolution in a mountain context, Vallone Inferno (scillato, Italy). *C. R. Palevol* **12**, 115–126 (2013).
217. Baker, K. H. *et al.* The 10,000-year biocultural history of fallow deer and its implications for conservation policy. *Proc. Natl. Acad. Sci. U.S.A.* **121**, e2310051121 (2024).
218. Savona-Ventura, C. & Mifsud, A. Għar Dalam Cave: A review of the sediments on the cave floor stratigraphy. *Xjenza* **3**, 5–12 (1998).

219. Vigne, J.-D. *et al.* Pre-Neolithic wild boar management and introduction to Cyprus more than 11,400 years ago. *Proc. Natl. Acad. Sci. U.S.A.* **106**, 16135–16138 (2009).
220. Heinsohn, T. Animal translocation: Long-term human influences of the vertebrate zoogeography of Australasia (natural dispersal versus ethnophoresy). *Aust. Zool.* **32**, 351–376 (2003).
221. Anderson, A. The rat and the octopus: Initial human colonization and the prehistoric introduction of domestic animals to Remote Oceania. *Biol. Invasions* **11**, 1503–1519 (2009).
222. Lister, A. M. The morphological distinction between bones and teeth of fallow deer (*Dama dama*) and red deer (*Cervus elaphus*). *Int. J. Osteoarchaeol.* **6**, 119–143 (1996)
223. McLaughlin, R. *et al.* An isotopic study of palaeodiet at the Circle and the Xemxija tombs in *Temple people: Bioarchaeology, resilience and culture in prehistoric Malta* (eds. Stoddart, S. et al.) 295–302 (McDonald Institute for Archaeological Research, 2022).
224. Richards, M. P., Hedges, R. E. M., Walton, I., Stoddart, S. & Malone, C. Neolithic diet at the Brochtorff Circle, Malta. *Eur. J. Archaeol.* **4**, 253–262 (2001).
225. Roberts, P. *et al.* Late Pleistocene to Holocene human palaeoecology in the tropical environments of coastal eastern Africa. *Palaeogeogr. Palaeoclimatol. Palaeoecol.* **537**, 109438 (2020).
226. Iminjili, V. *et al.* Late Pleistocene to late Holocene palaeoecology and human foraging at Kuumbi Cave, Zanzibar Island. *Front. Environ. Archaeol.* **2** (2023).
227. Craig, H. The geochemistry of the stable carbon isotopes. *Geochim. Cosmochim. Acta* **3**, 53–92 (1953).
228. Smith, B. N. & Epstein, S. Two categories of $^{13}C/^{12}C$ ratios for higher plants. *Plant Physiol.* **47**, 380–384 (1971).
229. Farquhar, G. D., Ehleringer, J. R. & Hubick, K. T. Carbon isotope discrimination and photosynthesis. *Annu. Rev. Plant Physiol. Plant Mol. Biol.* **40**, 503–537 (1989).
230. Groucutt, H. S. *et al.* The 4.2 ka Event and the End of the Maltese "Temple Period". *Front. Earth Sci.* **9** (2022).
231. Kohn, M. J. Carbon isotope compositions of terrestrial C3 plants as indicators of (paleo)ecology and (paleo)climate. *Proc. Natl. Acad. Sci. U.S.A.* **107**, 19691–19695 (2010).
232. Hartman, G. & Danin, A. Isotopic values of plants in relation to water availability in the eastern Mediterranean region. *Oecologia* **162**, 837–852 (2010).
233. Lee-Thorp, J. A. & van der Merwe, N. J. Carbon isotope analysis of fossil bone apatite. *S. Afr. J. Sci.* **83**, 712–715 (1987).
234. Cerling, T. E., Harris, J. M. & Passey, B. H. Diets of East African Bovidae based on stable isotope analysis. *J. Mammal.* **84**, 456–470 (2003).
235. Cerling, T. E., Harris, J. M., Leakey, M. G. & Mudida, N. Stable isotope ecology of northern Kenya with emphasis on the Turkana Basin in *Lothagam. The Dawn of Humanity in Eastern Africa* (eds. Leakey, M. G. & Harris, J. M.) 583–594 (Columbia University Press, 2003).
236. Dansgaard, W. Stable isotopes in precipitation. *Tellus* **16**, 436–468 (1964).
237. Rozanski, K., Araguás-Araguás, L. & Gonfiantini, R. Isotopic patterns in modern global precipitation in *Climate Change in Continental Isotopic Records* (eds. Swart, P. K., Lohmann, K. C., Mckenzie, J. & Savin, S.) 1–36 (American Geophysical Union, 1993).
238. Buchmann, N., Guehl, J.-M., Barigah, T. S. & Ehleringer, J. R. Interseasonal comparison of CO_2 concentrations, isotopic composition, and carbon dynamics in an Amazonian rainforest (French Guiana). *Oecologia* **110**, 120–131 (1997).
239. Buchmann, N. & Ehleringer, J. R. CO_2 concentration profiles, and carbon and oxygen isotopes in C_3 and C_4 crop canopies. *Agric. For. Meteorol.* **89**, 45–58 (1998).
240. McCarroll, D. & Loader, N. J. Stable isotopes in tree rings. *Quat. Sci. Rev.* **23**, 771–801 (2004).

241. Kohn, M. J., Schoeninger, M. J. & Valley, J. W. Herbivore tooth oxygen isotope compositions: Effects of diet and physiology. *Geochim. Cosmochim. Acta* **60**, 3889–3896 (1996).
242. Levin, N. E., Cerling, T. E., Passey, B. H., Harris, J. M. & Ehleringer, J. R. A stable isotope aridity index for terrestrial environments. *Proc. Natl. Acad. Sci. U.S.A.* **103**, 11201–11205 (2006).
243. Carter, M. L. & Bradbury, M. W. Oxygen isotope ratios in primate bone carbonate reflect amount of leaves and vertical stratification in the diet. *Am. J. Primatol.* **78**, 1086–1097 (2016).
244. Casula, P. & Murgia, A. Selectivity and context dependence of Corsican red deer browsing in a Mediterranean coppice system. *Hystrix* **28**, 157–164 (2017).
245. Khammes, N. & Aulagnier, S. Diet of the wood mouse, *Apodemus sylvaticus* in three biotopes of Kabylie of Djurdjura (Algeria). *Folia Zool.* **56**, 243–252 (2007).
