# Supplementary material for: Hunter-gatherer sea voyages extended to remotest Mediterranean islands
Source: Nature. 2025 Apr 9;641(8061):137–43. doi: 10.1038/s41586-025-08780-y (PMC12043505; doi:10.1038/s41586-025-08780-y)
Supplement: Supplementary file 2 — Reporting Summary [file 41586_2025_8780_MOESM2_ESM.pdf]

nature portfolio

Corresponding author(s): Eleanor Scerri
James Blinkhorn
Nicholas Vella

Last updated by author(s): 10/02/2025

Reporting Summary

Nature Portfolio wishes to improve the reproducibility of the work that we publish. This form provides structure for consistency and transparency in reporting. For further information on Nature Portfolio policies, see our Editorial Policies and the Editorial Policy Checklist.

Statistics

For all statistical analyses, confirm that the following items are present in the figure legend, table legend, main text, or Methods section.

n/a	Confirmed	
☐	☒	The exact sample size (*n*) for each experimental group/condition, given as a discrete number and unit of measurement
☐	☒	A statement on whether measurements were taken from distinct samples or whether the same sample was measured repeatedly
☐	☒	The statistical test(s) used AND whether they are one- or two-sided<br>*Only common tests should be described solely by name; describe more complex techniques in the Methods section.*
☐	☒	A description of all covariates tested
☐	☒	A description of any assumptions or corrections, such as tests of normality and adjustment for multiple comparisons
☐	☒	A full description of the statistical parameters including central tendency (e.g. means) or other basic estimates (e.g. regression coefficient) AND variation (e.g. standard deviation) or associated estimates of uncertainty (e.g. confidence intervals)
☒	☐	For null hypothesis testing, the test statistic (e.g. *F*, *t*, *r*) with confidence intervals, effect sizes, degrees of freedom and *P* value noted<br>*Give P values as exact values whenever suitable.*
☐	☒	For Bayesian analysis, information on the choice of priors and Markov chain Monte Carlo settings
☒	☐	For hierarchical and complex designs, identification of the appropriate level for tests and full reporting of outcomes
☒	☐	Estimates of effect sizes (e.g. Cohen's *d*, Pearson's *r*), indicating how they were calculated

Our web collection on statistics for biologists contains articles on many of the points above.

Software and code

Policy information about availability of computer code

Data collection	n/a
Data analysis	Oxcal 4.4. was used for chronological modelling, with the latest datasets of Intcal20 for the charcoal samples and Marine20 for the shells. Code details are shown in https://github.com/wccarleton/mesoneomalta

For manuscripts utilizing custom algorithms or software that are central to the research but not yet described in published literature, software must be made available to editors and reviewers. We strongly encourage code deposition in a community repository (e.g. GitHub). See the Nature Portfolio guidelines for submitting code & software for further information.

Data

Policy information about availability of data

All manuscripts must include a data availability statement. This statement should provide the following information, where applicable:

- Accession codes, unique identifiers, or web links for publicly available datasets
- A description of any restrictions on data availability
- For clinical datasets or third party data, please ensure that the statement adheres to our policy

Code and Data are available: https://github.com/wccarleton/mesoneomalta , and archived https://doi.org/10.5281/zenodo.14192393

Research involving human participants, their data, or biological material

Policy information about studies with human participants or human data. See also policy information about sex, gender (identity/presentation), and sexual orientation and race, ethnicity and racism.

Reporting on sex and gender	There were no human participants or human biological material used in this paper.
Reporting on race, ethnicity, or other socially relevant groupings	n/a
Population characteristics	n/a
Recruitment	n/a
Ethics oversight	n/a

Note that full information on the approval of the study protocol must also be provided in the manuscript.

Field-specific reporting

Please select the one below that is the best fit for your research. If you are not sure, read the appropriate sections before making your selection.

☐ Life sciences ☐ Behavioural & social sciences ☒ Ecological, evolutionary & environmental sciences

For a reference copy of the document with all sections, see nature.com/documents/nr-reporting-summary-flat.pdf

Ecological, evolutionary & environmental sciences study design

All studies must disclose on these points even when the disclosure is negative.

Study description	The study involved archaeological investigations of the material culture traces of past humans.
Research sample	100% sampling of sediments was undertaken to recover archaeobotanical remains (phytoliths, anthracology, pollen). All material culture and faunal remains were collected and recorded using a Total Station where they measured over 2mm, with the exception of microfaunal remains. Charcoal was recovered from the sediments and large pieces collected in situ and recorded using a total station for chronometric dating.
Sampling strategy	Archaeobotanical analyses involved 100% sampling and all sediments were floated and analysed. Charcoal for chronomtric dating was selected from key contexts using the Harris Matrix and features such as hearths to make decisions on Phase boundaries and the remains of human activity.
Data collection	Data was collected during excavation. Context sheets, photographs, photogrammetry and total station recording as well as plan drawings were used to ensure detailed and correct documentation. All samples, artefacts and bones were also recorded using a total station in order to reconstruct their location in three dimensions.
Timing and spatial scale	Data was collected during three field seasons. Field seasons in 2021 (4 weeks), 2022 (4 weeks) and 2023 (4 weeks) provided the data reported in the paper. The Data was recovered from a 5 x 5 m trench.
Data exclusions	No data was excluded.
Reproducibility	We provide tables of raw data and analytical code, as well as abundant details on the location and character of scientific samples that ensure reproducibility.
Randomization	This is not relevant to an archaeological study reporting the results of an excavation.
Blinding	For the reasons described above, this is not relevant.

Did the study involve field work? ☒ Yes ☐ No

Field work, collection and transport

Field conditions	The fieldwork was conducted in the summer months of August and September which was mostly warm and dry with the exception of some storm events. Thanks to preparation involving sand bags and rainproof covers, these storms did not impact the excavations.
Location	Mellieha, Malta 35.977703617820254, 14.328625741239756

Access & import/export	All excavation work was conducted under permits provided by the Maltese Superintendence of Cultural Heritage and the Environmental Resource Agency. Individual samples exported for analyses were given individual permissions for export and analysis by the Superintendence of Cultural Heritage.
Disturbance	The area of the trench has been disturbed by excavation, but the trench is backfilled every year and there is no impact on the broader location of the trench.

Reporting for specific materials, systems and methods

We require information from authors about some types of materials, experimental systems and methods used in many studies. Here, indicate whether each material, system or method listed is relevant to your study. If you are not sure if a list item applies to your research, read the appropriate section before selecting a response.

Materials & experimental systems

n/a	Involved in the study	
☒	☐	Antibodies
☒	☐	Eukaryotic cell lines
☐	☒	Palaeontology and archaeology
☒	☐	Animals and other organisms
☒	☐	Clinical data
☒	☐	Dual use research of concern
☐	☒	Plants

Methods

n/a	Involved in the study	
☒	☐	ChIP-seq
☒	☐	Flow cytometry
☒	☐	MRI-based neuroimaging

Palaeontology and Archaeology

Specimen provenance	Faunal remains were recovered from the site. Most of the analyses on the faunal remains have taken place in Malta under the excavation permit. All faunal remains that have been exported for specific analyses have the permission of the Superintendence of Cultural Heritage on an individual basis.
Specimen deposition	Currently the specimens are being held at the University of Malta. They will be given to Heritage Malta for accessioning and long term storage once the project is completed.
Dating methods	Radiocarbon dating was used on charcoal. One date was obtained on bone. Permission for the expert of both bone and charcoal was sought and received from the Superintendence of Cultural Heritage. ABA Pre-treatment was used at the CEZA institute in Mannheim Germany, for the charcoal remains. The bone date was obtained from the Center for Applied Isotope Studies (CAIS) in the U.S. Details of treatments are given in the methods section of the main text.

☒ Tick this box to confirm that the raw and calibrated dates are available in the paper or in Supplementary Information.

Ethics oversight	No ethical approval or guidance was required for the dating of charcoal and faunal remains.

Note that full information on the approval of the study protocol must also be provided in the manuscript.

Dual use research of concern

Policy information about dual use research of concern

Hazards

Could the accidental, deliberate or reckless misuse of agents or technologies generated in the work, or the application of information presented in the manuscript, pose a threat to:

No	Yes	
☒	☐	Public health
☒	☐	National security
☒	☐	Crops and/or livestock
☒	☐	Ecosystems
☒	☐	Any other significant area

Experiments of concern

Does the work involve any of these experiments of concern:

No	Yes	
☒	☐	Demonstrate how to render a vaccine ineffective
☒	☐	Confer resistance to therapeutically useful antibiotics or antiviral agents
☒	☐	Enhance the virulence of a pathogen or render a nonpathogen virulent
☒	☐	Increase transmissibility of a pathogen
☒	☐	Alter the host range of a pathogen
☒	☐	Enable evasion of diagnostic/detection modalities
☒	☐	Enable the weaponization of a biological agent or toxin
☒	☐	Any other potentially harmful combination of experiments and agents

Plants

Seed stocks	No seed stocks were used. We looked at burned and mineralized plant remains as well as pollen.
Novel plant genotypes	N/a
Authentication	N/a
