# Supplementary material for: Author Correction: Hunter-gatherer sea voyages extended to remotest Mediterranean islands
Source: Nature. 2026 Jan 29;650(8102):E10. doi: 10.1038/s41586-025-10024-y (PMC12916309; doi:10.1038/s41586-025-10024-y)
Supplement: Supplementary file 1 — Supplementary Fig. 1 and Supplementary Tables 1 and 2 [file 41586_2025_10024_MOESM1_ESM.pdf]

nature portfolio

https://doi.org/10.1038/s41586-025-10024-y

Supplementary information

Author Correction: Hunter-gatherer sea voyages extended to remotest Mediterranean islands

In the format provided by the authors and unedited

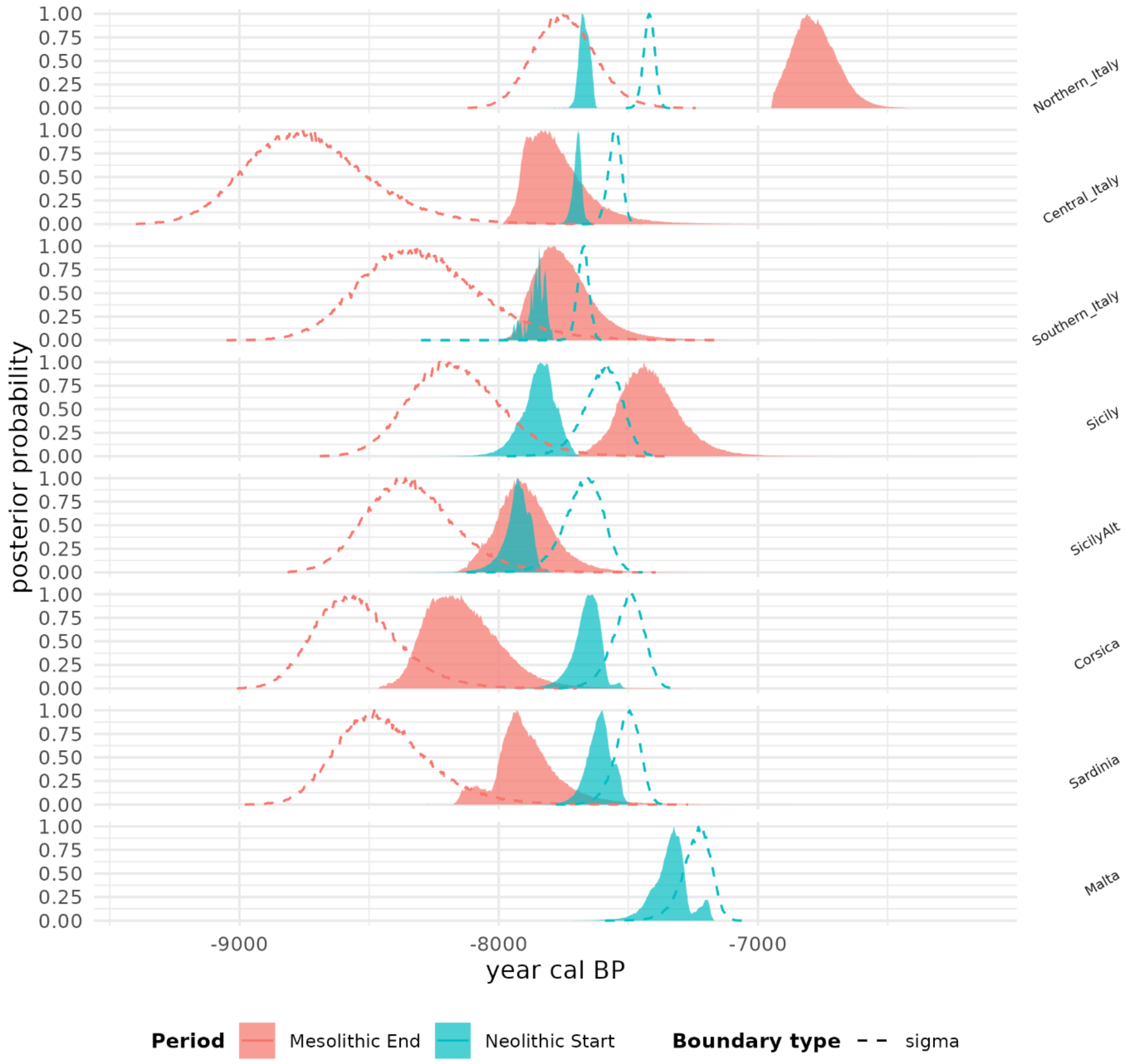

Supplementary Figure 1: Corrected Regional Boundary Posteriors. As the legend indicates, dashed lines represent the ‘sigma’ type boundary whereas filled areas represent the ‘uniform’ type.

region	boundary	expected_age	lower_95_hdr	upper_95_hdr	model_variant
Central_Italy	mesolithic_end	-6776.9	-7224.5	-6299.5	corrected_sigma
Central_Italy	mesolithic_end	-5816.6	-6004.5	-5559.5	corrected_uniform
Central_Italy	mesolithic_end	-5833.3	-6009.5	-5599.5	original
Central_Italy	neolithic_start	-5602.2	-5649.5	-5559.5	corrected_sigma
Central_Italy	neolithic_start	-5747.6	-5784.5	-5719.5	corrected_uniform
Central_Italy	neolithic_start	-5715	-5754.5	-5674.5	original
Corsica_mesolithic	mesolithic_end	-6590.6	-6899.5	-6269.5	corrected_sigma
Corsica_mesolithic	mesolithic_end	-6188	-6424.5	-5929.5	corrected_uniform
Corsica_mesolithic	mesolithic_end	-6108.7	-6344.5	-5869.5	original
Corsica_neolithic	neolithic_start	-5555.2	-5674.5	-5439.5	corrected_sigma
Corsica_neolithic	neolithic_start	-5711.8	-5829.5	-5629.5	corrected_uniform
Corsica_neolithic	neolithic_start	-5673	-5804.5	-5544.5	original
Malta_neolithic	neolithic_start	-5289.8	-5404.5	-5189.5	corrected_sigma
Malta_neolithic	neolithic_start	-5398.3	-5509.5	-5289.5	corrected_uniform
Malta_neolithic	neolithic_start	-5391.5	-5509.5	-5254.5	original
Northern_Italy	mesolithic_end	-5793.4	-6014.5	-5559.5	corrected_sigma
Northern_Italy	mesolithic_end	-4825.2	-4969.5	-4684.5	corrected_uniform
Northern_Italy	mesolithic_end	-5797.3	-6019.5	-5569.5	original
Northern_Italy	neolithic_start	-5469.9	-5509.5	-5434.5	corrected_sigma
Northern_Italy	neolithic_start	-5714.5	-5744.5	-5679.5	corrected_uniform
Northern_Italy	neolithic_start	-5709.8	-5744.5	-5674.5	original
Sardinia_mesolithic	mesolithic_end	-6489.5	-6824.5	-6124.5	corrected_sigma
Sardinia_mesolithic	mesolithic_end	-5938.5	-6199.5	-5694.5	corrected_uniform
Sardinia_mesolithic	mesolithic_end	-5973.4	-6219.5	-5629.5	original
Sardinia_neolithic	neolithic_start	-5557.4	-5654.5	-5469.5	corrected_sigma
Sardinia_neolithic	neolithic_start	-5661.1	-5754.5	-5574.5	corrected_uniform
Sardinia_neolithic	neolithic_start	-5661.1	-5759.5	-5569.5	original
SicilyAlt_mesolithic	mesolithic_end	-6386.1	-6709.5	-6049.5	corrected_sigma
SicilyAlt_mesolithic	mesolithic_end	-5948.3	-6164.5	-5749.5	corrected_uniform
SicilyAlt_neolithic	neolithic_start	-5726.1	-5879.5	-5589.5	corrected_sigma
SicilyAlt_neolithic	neolithic_start	-5979.5	-6084.5	-5894.5	corrected_uniform
Sicily_mesolithic	mesolithic_end	-6210.4	-6544.5	-5864.5	corrected_sigma
Sicily_mesolithic	mesolithic_end	-5461.3	-5679.5	-5224.5	corrected_uniform
Sicily_mesolithic	mesolithic_end	-5525.9	-5809.5	-5249.5	original
Sicily_neolithic	neolithic_start	-5652.6	-5799.5	-5514.5	corrected_sigma
Sicily_neolithic	neolithic_start	-5897.9	-6024.5	-5774.5	corrected_uniform
Sicily_neolithic	neolithic_start	-5837.6	-5999.5	-5719.5	original
Southern_Italy	mesolithic_end	-6344.8	-6779.5	-5874.5	corrected_sigma
Southern_Italy	mesolithic_end	-5791	-5984.5	-5549.5	corrected_uniform
Southern_Italy	mesolithic_end	-5779.2	-5979.5	-5524.5	original
Southern_Italy	neolithic_start	-5725.3	-5764.5	-5689.5	corrected_sigma
Southern_Italy	neolithic_start	-5915.3	-5984.5	-5854.5	corrected_uniform
Southern_Italy	neolithic_start	-5860.5	-5904.5	-5819.5	original

Supplementary Table 1: Original compared to corrected posterior boundaries (upper and lower limits of the 95% credible region). Corrected boundary types are indicated in the 'model_variant' column).

	95% sigma	95% uniform	Model version
**Start of Neolithic in Sicily**	/	7949.5-7669.5 cal BP	As initially published.
	7749.5-7464.5 cal BP	7974.5-7724.5 cal BP	With updated uncertainties (i.e. corrected version).
	7829.5-7539.5 cal BP	8034.5-7844.5 cal BP	SicilyAlt-Neolithic: With alternative interpretation of samples MAMS-40712, MAMS-48212, KIA-36032.
**End of Mesolithic in Sicily**	/	7759-7199.5 cal BP	As initially Published.
	8484.5-7815.5 cal BP	7629.5-7174.5 cal BP	With updated uncertainties (i.e. corrected version).
	8659.5-7999.5 cal BP	8114.5-7699.5 cal BP	SicilyAlt-Mesolithic: With alternative interpretation of samples MAMS-40712, MAMS-48212, KIA-36032.

Supplementary Table 2: Summary of different chronological modelling results for the Mesolithic to Neolithic transition in Sicily.
